# Mechanistic Insights into the Chaperoning of Human Lysosomal-Galactosidase Activity: Highly Functionalized Aminocyclopentanes and *C*-5a-Substituted Derivatives of 4-*epi*-Isofagomine

**DOI:** 10.3390/molecules25174025

**Published:** 2020-09-03

**Authors:** Patrick Weber, Martin Thonhofer, Summer Averill, Gideon J. Davies, Andres Gonzalez Santana, Philipp Müller, Seyed A. Nasseri, Wendy A. Offen, Bettina M. Pabst, Eduard Paschke, Michael Schalli, Ana Torvisco, Marion Tschernutter, Christina Tysoe, Werner Windischhofer, Stephen G. Withers, Andreas Wolfsgruber, Tanja M. Wrodnigg, Arnold E. Stütz

**Affiliations:** 1 Glycogroup, Institute of Chemistry and Technology of Biobased Systems, Graz University of Technology, Stremayrgasse 9, A-8010 Graz, Austria; patrick.weber@tugraz.at (P.W.); thonhofer@tugraz.at (M.T.); seaveril@uvm.edu (S.A.); michael.schalli@medunigraz.at (M.S.); andreas.wolfsgruber@tugraz.at (A.W.); t.wrodnigg@tugraz.at (T.M.W.); 2 Department of Chemistry, University of York, Heslington, York YO10 5DD, North Yorkshire, UK; gideon.davies@york.ac.uk (G.J.D.); wendy.offen@york.ac.uk (W.A.O.); 3 Chemistry Department, University of British Columbia, 2036 Main Mall, Vancouver, BC V6T 1Z1, Canada; aglez@chem.ubc.ca (A.G.S.); snasseri@chem.ubc.ca (S.A.N.); christina.tysoe@gmail.com (C.T.); withers@chem.ubc.ca (S.G.W.); 4 Institute of Inorganic Chemistry, Graz University of Technology, Stremayrgasse 9, A-8010 Graz, Austria; philipp.mueller@tugraz.at (P.M.); ana.torviscogomez@tugraz.at (A.T.); 5 Laboratory of Metabolic Diseases, Department of Pediatrics, MedUni Graz, Auenbruggerplatz 30, A-8036 Graz, Austria; bettina.pabst@medunigraz.at (B.M.P.); eduard.paschke@inode.at (E.P.); marion.tschernutter@medunigraz.at (M.T.); werner.windischhofer@medunigraz.at (W.W.)

**Keywords:** iminoalditol, 4-*epi*-isofagomine, carbasugar, aminocyclopentane, galactosidase inhibitor, pharmacological chaperone, G_M1_-gangliosidosis

## Abstract

Glycosidase inhibitors have shown great potential as pharmacological chaperones for lysosomal storage diseases. In light of this, a series of new cyclopentanoid β-galactosidase inhibitors were prepared and their inhibitory and pharmacological chaperoning activities determined and compared with those of lipophilic analogs of the potent β-d-galactosidase inhibitor 4-*epi*-isofagomine. Structure-activity relationships were investigated by X-ray crystallography as well as by alterations in the cyclopentane moiety such as deoxygenation and replacement by fluorine of a “strategic” hydroxyl group. New compounds have revealed highly promising activities with a range of β-galactosidase-compromised human cell lines and may serve as leads towards new pharmacological chaperones for G_M1_-gangliosidosis and Morquio B disease.

## 1. Introduction

Many glycoside hydrolases play important roles in various pathological processes, including certain hereditary lysosomal disorders [1,2,3]. A group of about fifty of these metabolic diseases arises from mutations in specific genes that lead to deficiencies in enzymes involved in the lysosomal degradation of glycolipids and glycans. Considerable efforts have been made to develop novel therapeutics that may relieve the symptoms arising from the cellular accumulation of the substrates of these deficient enzymes. These toxic accumulations, in turn, lead to irreversible damage of nerve tissue and bones as well as various organs. For some of these lysosomal disorders, enzyme replacement therapy [4,5], hematopoietic stem cell transplant [6,7], gene therapy [8,9], as well as combination therapies [10] involving the administration of small molecules show considerable promise. These small molecules may function by one of the following two mechanisms: Either by substrate reduction therapy [11,12,13]—inhibiting upstream enzymes to reduce the initial production of those metabolites whose degradation is impaired or as enzyme-stabilizing pharmacological chaperones by assisting the folding and transport of mutant enzymes to the lysosome using sub-inhibitory concentrations of active site-specific molecules (chaperone mediated therapy, CMT) [14,15,16,17,18,19,20,21,22].

Efforts of many groups have shown that imino sugar- as well as carba sugar-based glycomimetics may be suitable as such therapeutic agents. Mutant proteins that cannot obtain/retain their functional conformation are recognized as misfolded by the quality control machinery in the endoplasmic reticulum and are eventually targeted for degradation. In CMT, the carba or imino sugars bind to and stabilize mutant forms of enzymes (such as lysosomal β-glucosidase, β-galactosidase or β-*N*-acetylhexosaminidase) in their functional folded conformations and thus facilitate their exit from the endoplasmic reticulum and their subsequent transport to the lysosome.

**Figure 1:**
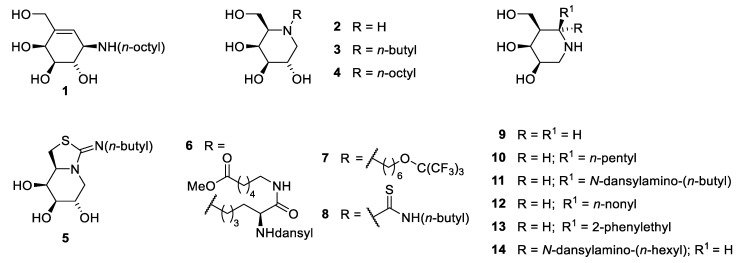
Galactosidase inhibitors as potential therapeutic chaperones for mutation induced deficiencies of lysosomal β-galactosidase.

Guiding contributions by various leaders in the field and recent reviews [23,24,25,26] have shown that non-polar *N*-substituted iminoalditols and similarly functionalized structures bind better than their more polar parent compounds. This is achieved through stronger interactions with the aglycon binding site or with lipophilic pockets around the active site. In this context, *N*-butyl-1-deoxynojirimycin is one of the best studied iminosugars, thus far [27]. Other researchers [28,29,30,31] have observed that large, lipophilic substituents, for example, adamantyl capped spacer arms, greatly improve the interaction between iminoalditol derivatives and the lysosomal β-glucosidase (Figure 1).

G_M1_-Gangliosidosis and Morquio B disease are two manifestations of mutations on the *GLB1* gene that encodes the vital lysosomal β-galactosidase. This enzyme is responsible for removal of a β-galactosyl residue from the non-reducing end of its substrate, converting gangliosides G_M1_ and G_A1_ into G_M2_ and G_A2_, respectively. Depending on the location of the mutation, the resulting lysosomal storage disorder is neurodegenerative (G_M1_-gangliosidosis) or bone-destructive (Morquio B) [32,33].

Three distinct structural types of β-galactosidase inhibitors have been investigated as potential pharmacological chaperones for these two disorders. The first class is that of the *epi*-valienamines, of which NOEV (*N*-octyl-*epi*-valienamine, **1**, Figure 1) is a powerful β-galactosidase inhibitor [34] and reportedly functions as a highly potent pharmacological chaperone for a wide range of mutants of lysosomal β-galactosidase, even at sub-micromolar concentrations [35]. More recently, selected un-branched analogs lacking the hydroxymethyl group have been presented and their activities with patients’ skin fibroblasts carrying the R201C mutation have been evaluated [36]. The second class of potential chaperones are the hydroxylated piperidines exemplified by the deoxygalactonojirimycins, **2–4**. Through a variety of structural modifications, this scaffold has been transformed into a relatively small number of derivative classes. Compounds **3**–**8** show several of the most active *N*-substituent-variants in the literature, which have interesting properties as experimental pharmacological chaperones. [22,37,38,39,40,41,42,43,44]. *C*-5a-substituted derivatives of the known [45] isoiminosugar 4-*epi*-isofagomine (**9**), for example, compounds **10**–**14** have recently been reported and shown to be potent β-galactosidase inhibitors and superior pharmacological chaperones for selected G_M1_-gangliosidosis and Morquio B disease cell lines [46,47,48,49,50].

**Figure 2:**
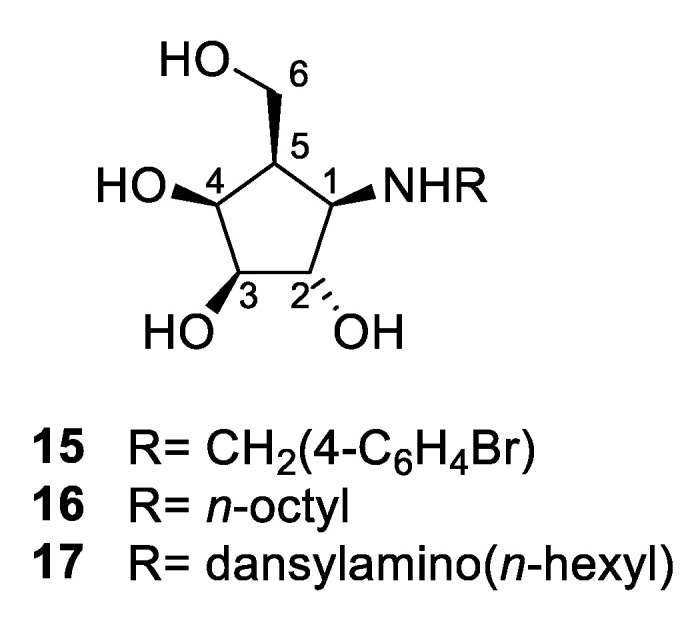
Inhibitors **15**–**17**.

The third class are the hydroxymethyl branched di- and trihydroxycyclopentylamines, which were recently introduced as experimental pharmacological chaperones [51]. These compounds may be regarded as products of ring contraction by formal homolytic extraction of the sugar ring oxygen and bond formation between C-1 and C-5. The resulting carbacyclic scaffold maintains the stereochemical information of the corresponding parent sugar or glycosylamine albeit with the characteristic conformational features of five-membered rings. There are only three leading references presenting such sugar analogues as proven d-galactosidase inhibitors [52,53,54]. Even more surprisingly, this family of compounds reportedly harbours some of the most powerful inhibitors of β-galactosidases known to date with inhibition constants in the sub-nanomolar range [52,53,54]. In particular, the β-d-*galacto* configured cyclopentylamine bearing a 4-bromobenzyl moiety at the nitrogen (**15**, Figure 2), has been found to excel in terms of inhibitory activity [52,53,54] and recently prepared [55] compounds **16** and **17** are powerful inhibitors of human lysosomal β-galactosidase and potent pharmacological chaperones for the R201C mutant of this enzyme.

In this study, which aims to extend the selection of chaperone structures useful for treating G_M1_-gangliosidosis and Morquio B, we have investigated the effect of 4-*epi*-isofagomine derivatives featuring dansylamino capped medium chain length aliphatic substituents. We have furthermore explored the biochemical role of the OH group at C-2, which is a key functional group in natural β-galactosidase substrates but is not present in the 4-*epi*-isofagomine inhibitors. This has consequences both on the specific binding interactions formed and indirectly, on the basicity of the ring nitrogen compared to the β-hydroxy substituted imino (**2–8**) or carba sugars such as **15**, **16** and **17**.

## 2. Results

### 2.1. Synthesis of 4-epi-Isofagomines

**Scheme 1:**
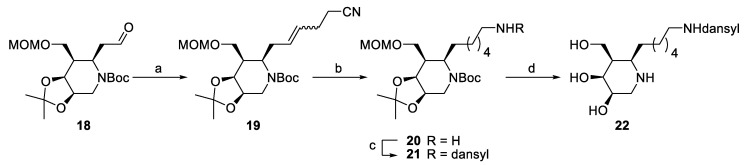
Synthesis of inhibitor **22**. **a**: Ph_3_P(CH_2_)_3_CN Br, LDA, THF_(abs)_, N_2_, −78 °C to r.t., 66%; **b**: Raney-Ni, H_2_, MeOH, 84%; **c**: dansyl chloride, NaHCO_3_, MeOH, 61%; d: HCl/MeOH, 74%.

By analogy to the synthesis of (5a*S*)-dansylaminohexyl derivative **14** [46,47], (Figure 1) its (5a*R*)-epimer **22** was prepared from known intermediate aldehyde **18** [56], by Wittig chain extension employing triphenyl-(3-cyano)propyl phosphonium bromide to obtain nitrile **19**. Subsequent terminal modification, via primary amine **20** and protected 4-*epi*-isofagomine derivative **21** provided free compound **22** featuring a dansyl capped aminohexyl chain at *R*-configured C-5a (Scheme 1). 

### 2.2. Synthesis of Cyclopentylamines

**Scheme 2:**
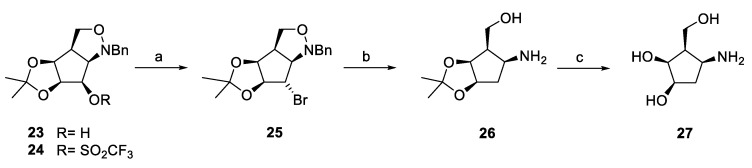
Synthesis of 2-deoxy intermediate **26**. **a**: (1) Tf_2_O, pyridine, CH_2_Cl_2_, 0 °C; (2) NaBr, DMF, 53% (2 steps); **b**: Raney-Ni, H_2,_ MeOH, 66%; **c**: HCl/MeOH, 88%.

Dansylaminohexyl substituted cyclopentane **17** was synthesized as described [55]. The corresponding deoxy derivative **31** was prepared from the recently reported [57] all-*cis* epimer **23** (Scheme 2). Activation of the free alcohol as a triflate (**24**) and treatment of the latter with sodium bromide in DMF gave bromodeoxy derivative **25** with clean inversion of configuration. Simultaneous removal of the bromo substituent and reductive isoxazolidine ring opening were achieved by use of Raney-Ni/H_2_ in MeOH, providing aminoalcohol **26** which, upon acid treatment, furnished free aminotriol **27** ready for planned chemoselective *N*-alkylation [48,49,51], but, as a consequence of solubility problems, this reaction was subsequently conducted with partially protected intermediate **26** (Scheme 2).

**Scheme 3:**
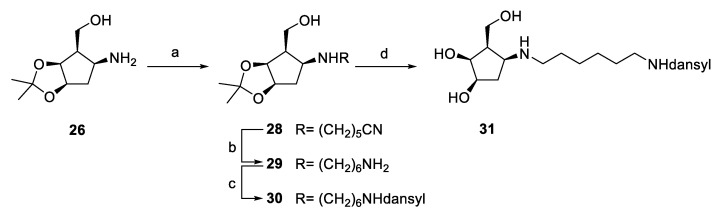
Synthesis of compound **31**. **a**: Br(CH_2_)_5_CN, NaHCO_3_, DMF, 70 °C, 62%; **b**: Raney-Ni, H_2_, MeOH, 78%; **c**: (1) dansyl chloride, Et_3_N, CH_2_Cl_2_-DMF (3:1); **d**: HCl/MeOH, 84% (2 steps).

Isopropylidene-protected amine **26** was chemoselectively alkylated to provide nitrile **28** (Scheme 3) which, in turn, was cleanly converted into primary amine **29** by catalytic hydrogenation over Raney-Ni. Subsequent *N*-dansylation (**30**) and removal of the isopropylidene moiety gave deprotected deoxy derivative **31** of parent compound **17** (Scheme 3).

**Scheme 4:**
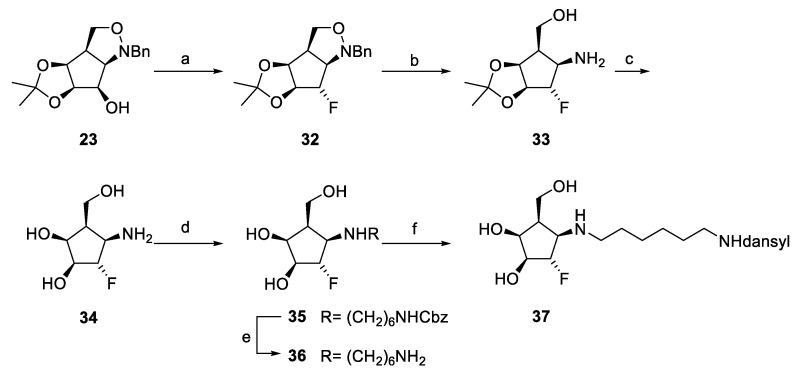
Synthesis of 2-deoxy-2-fluoro compound **37**. **a**: DAST, pyridine, CH_2_Cl_2_, 0 °C, 82%; **b**: Pd/C, H_2_, MeOH, 98%; **c**: HCl/MeOH, 92%; **d**: HCO(CH_2_)_6_NHCbz, NaCNBH_3_, AcOH, MeOH, 60%; **e**: Pd/C, H_2_, MeOH; **f**: dansyl chloride, Et_3_N, CH_3_CN–H_2_O (5:1), 53%.

In order to evaluate the influence of “polar deoxygenation” at C-2, the novel deoxyfluoro analogue **37** was synthesized by reaction of precursor **23** with DAST to furnish compound **32** and subsequent structural elaboration via intermediates **33**–**36** by analogous methods to those described for derivative **31** (Scheme 4).

**Figure 3:**
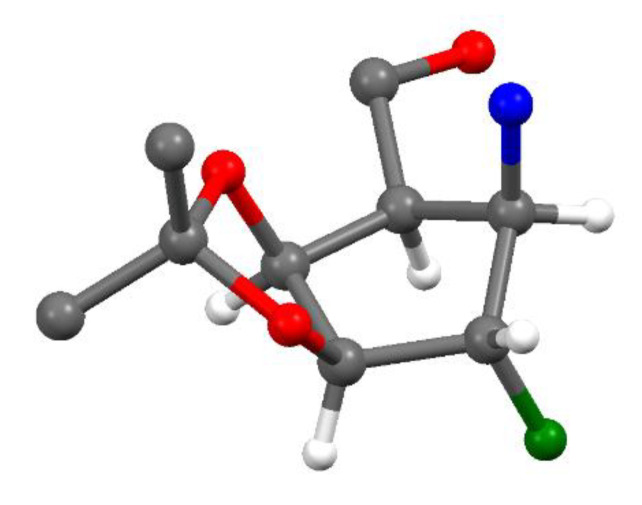
Crystal structure of compound **33** (CCDC 2018297).

The structure of intermediate **33** was confirmed by X-ray analysis (Figure 3).

### 2.3. Inhibitory Activities

Results of screening of new isofagomine derivative **22** as well as cyclopentanes **31** and **37** in comparison with their previously reported respective relatives **11**, **16** and **17** as well as pyranoid carbasugar derivative **1** employing a panel of representative d-galactosidases and d-glucosidases are collected in Table 1. Inhibition constants across the series tend to favor the binding of the piperidine structure **22**, with all β-glycosidases tested being inhibited in the low to sub-nanomolar range. Close behind this in affinity comes the new dansylated cyclopentanoid inhibitor **17**. Most of the β-glycosidases are inhibited in the low, but not sub-, nanomolar range, with the exceptions being the human lysosomal β-galactosidase (from CAZY [58,59] family GH35) and human lysosomal β-glucocerebrosidase.

Inspection of the structures of complexes with a bacterial GH35 β-galactosidase (see below) reveals an exquisite set of interactions between the active site residues and the polar groups of **22**, including a potentially electrostatic interaction between the catalytic nucleophile, Glu349, and the ring nitrogen. Most of these interactions are essentially replicated in the complex with **17**. Importantly, in the absence of an endocyclic nitrogen partner, Glu 349 now forms a strong interaction with the hydroxyl group at C-2, the secondary carbon adjacent to the alkylamine substituent on the ring. The importance of this interaction is clearly seen in comparing the *K*_i_ value of the GH35 bovine β-galactosidase with **17** and **31**. These inhibitors differ only in the presence of the hydroxyl group at that position, and indeed **31** binds some 20 fold less tightly, corresponding to an approximate 2 kcal/mol difference. An even bigger 200 fold affinity difference is seen for the human lysosomal β-galactosidase (GH35), implying crucial interactions of this particular hydroxyl group with GH35 enzymes. The substitution of a fluorine at that position rather than a hydroxyl group has similarly negative consequences. In the cases of both the mammalian β-galactosidases, the fluorine analogue binds in similar ranges as the parent with a hydrogen at that position but more than 12 times (bovine liver) to approx. 90 times (human lysosomal) worse than the hydroxylated version, implying highly reduced stabilizing interaction at that position. Binding to the GH1 and GH2 bacterial glycosidases is also considerably worse, by 64 and approx. 500 fold respectively, when compared to inhibitor **17**. Since the (minimal) size difference of F vs. H is unlikely to result in such significant changes, a destabilizing dipolar interaction of the C-F-system with a dipole in the active site is the most probable root cause. Indeed the crystal structure reveals that this could arise from unfavorable interactions of the fluorine atom with the carboxylate group of Glu349, since no hydrogen bond like that seen with the hydroxyl of **17**, can be formed. The strongly electron-withdrawing effect of the fluorine atom may also cause a significant reduction of the amine’s basicity (when compared to parent compound **17** and the more basic deoxy derivative **31**), which in turn could affect binding.

### 2.4. Inhibitor Binding in Crystal Structures of a Model β-Galactosidase from Cellvibrio Japonicus

**Figure 4:**
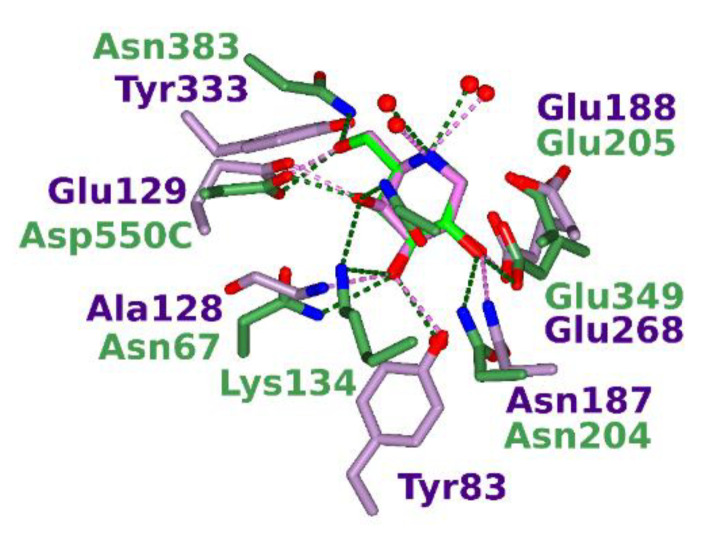
Overlay of DGJ in structures with human β-gal and *Cj*GH35. Side-chains, and main-chain for Ala128 in human β-gal, forming hydrogen bonds (shown as dashed lines) with DGJ in 3THD.pdb and 4D1J.pdb respectively are shown, as well as the catalytic acid/base residues (Glu188 and 205 respectively). NB Active sites are from molecules A, but Asp550 is from molecule C in *Cj*GH35. C atoms are colored lilac for human β-gal and lawn green for *Cj*GH35 (with ligand C atoms in orchid and green respectively). The label for Asn135 for *Cj*GH35 is omitted for clarity. Figure was made using *CCP*4*mg* [60].

Based on X-ray crystallographic studies of compounds **1**, **14**, **16**, **17** as well as **22** and **31** in complex with *Cellvibrio japonicus* β-galactosidase (*Cj*GH35), a tractable bacterial model which shows around 27% sequence identity with the human enzyme, notably conserved active centers, intensities of inhibition and chaperoning properties, may be partially rationalized (Figure 4).

**Figure 5:**
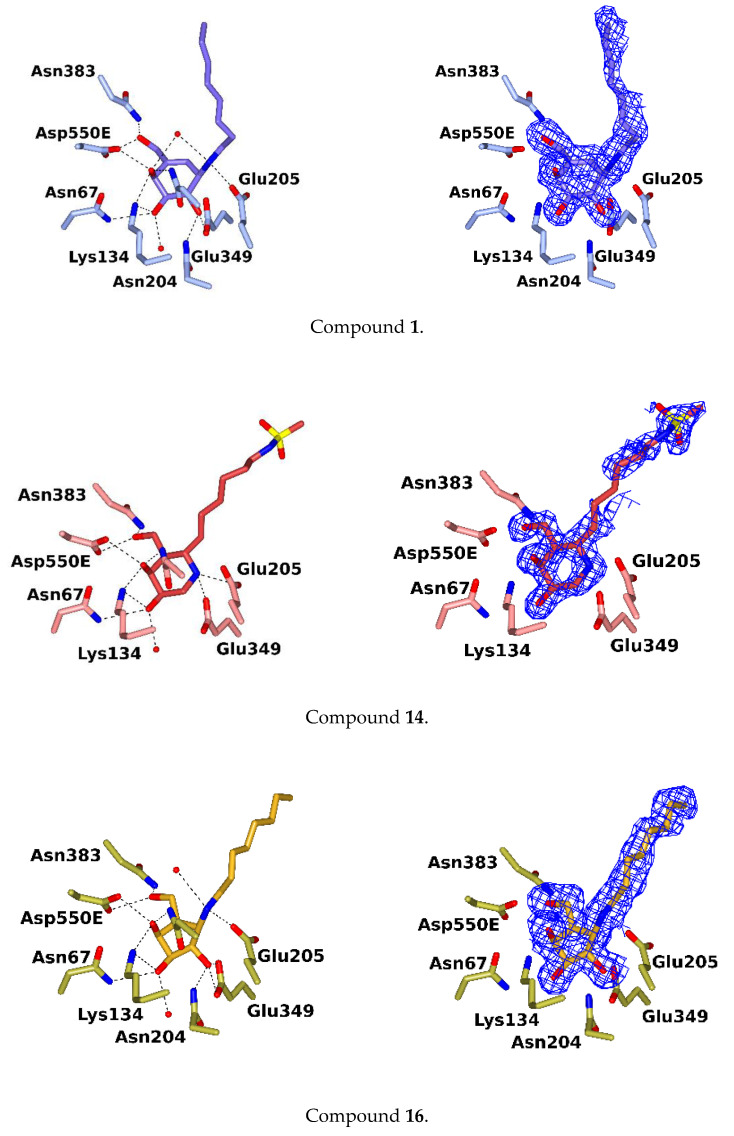

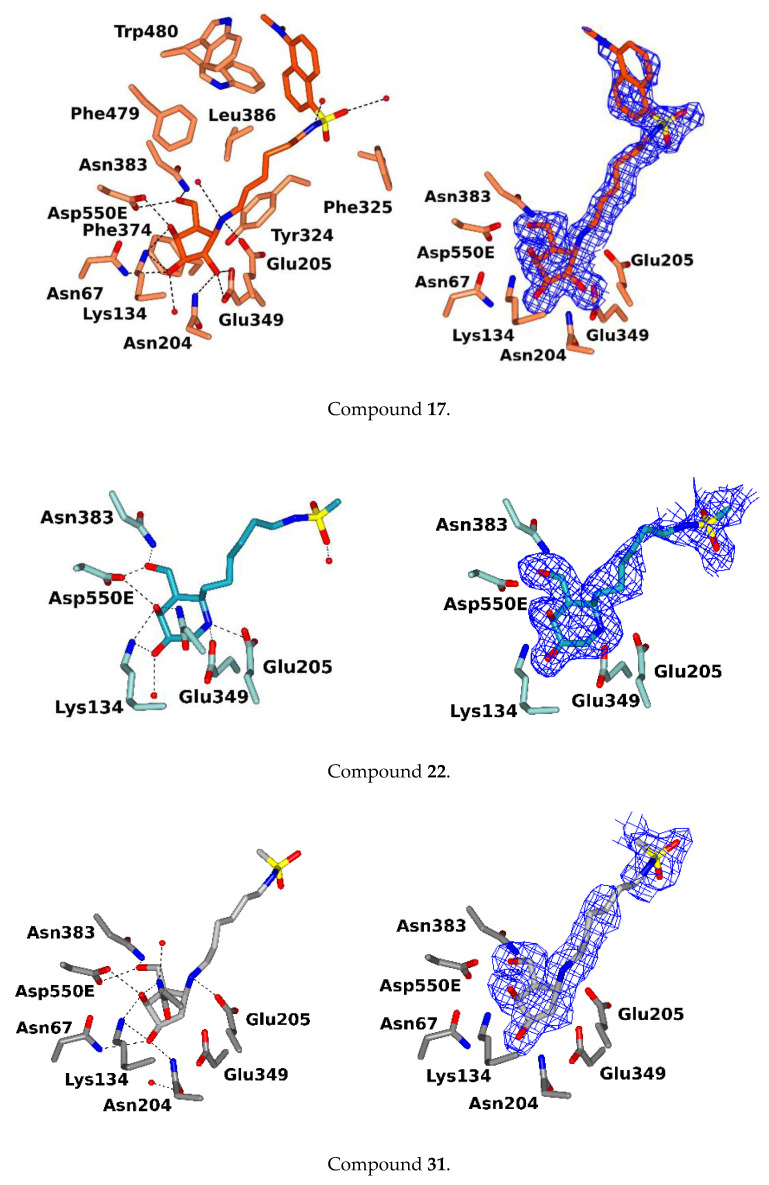
Illustration of ligand complexes with *Cj*GH35, showing hydrogen-bonding interactions (as dashed lines) between the modelled ligand atoms and protein side-chains, and ligand electron density (calculated as omit difference density maps contoured at 2 rmsd). For **17**, side-chains involved in aromatic interactions within 3.8 Å of the NH-dansyl group are also shown. The side-chain of Asn135 is omitted from the interactions figure for **17**, and the electron density pictures to aid clarity.

Figure 4 shows an overlay of the ligand molecules in the X-ray structures for human lysosomal β-d-galactosidase (human β-gal) and *Cj*GH35 complexed with 1-deoxygalactonojirimycin (3THD.pdb and 4D1J.pdb respectively). The catalytic residues lie in a similar position in the two structures, with the nucleophiles (Glu268 and Glu349) accepting a hydrogen bond from the ligand 2-OH group. Both enzymes also feature an asparagine adjacent to the acid/base (at 187 and 204 respectively), which interacts with the 2-OH via its ND2 group, and a carboxyl group which tethers the 4-OH and 6-OH groups (in human β-gal this is from Glu129, which is on a loop in the TIM barrel domain, whereas in the *Cj*GH35 crystal this is provided by Asp550 in the C-terminal domain of an adjacent protein chain). For the inhibitors studied here, the ligand sugar moieties have clear density in all the active sites and overlay with ring C atoms 2 to 5 in similar positions (Figure 5). Figure 5 displays hydrogen bonding interactions between amino acid residues in the active site of *Cj*GH35 and ligands **1**, **14**, **16**, **17**, **22** as well as **31** respectively. The side-chains of Asn135 (which forms hydrogen bonds of 3.0 Å from ND2 to C4-OH) and of Asp550E have been omitted for clarity.

For all the C-1-substituents in **1**, **16**, **17** and **31**, (an NH-octyl group in **1** and **16**, and NH-C_6_-NH-dansyl groups in **17** and **31**), and the C-5a-substituted NH-C_6_-NH-dansyl groups in **14** and **22**, there are very few interactions between the protein and substituted derivative moieties. The NH-C_6_-NH-dansyl group was only able to be modelled for **17**, and only at an occupancy of 0.5. For molecules **14**, **22** and **31** there is no electron density for the 5-(dimethylamino)naphthalene moiety. The active site residues are similarly oriented in all of the structures, with slight differences for **31**, as mentioned below.

The most salient variation in hydrogen bonding occurs at the 2-OH group, which is present in **1**, **16** and **17**. The group is tethered by interactions with ND2 Asn204 (all 2.8–3.0 Å) and to both OE atoms of Glu349 (2.6–2.7 Å) Hydrogen bonding patterns for **16** and **17** are very similar, despite the different groups bound at C-1, which are probably flexible and show few polar interactions with the protein, and not surprisingly, the *K*_i_ values determined for these inhibitors with recombinant human lysosomal β-Gal are very similar. Ligand **31** is identical to **17** apart from lacking a hydroxyl group at C-2, the importance of which is reflected in a much poorer *K*_i_ value (200-fold worse for human lysosomal β-galactosidase). There are two other differences in the ligand interactions of **31** (not only from those seen for **17**, but also from the other complexes studied here). Firstly, there is a hydrogen bond from the C-3 hydroxyl to ND2 Asn204, in addition to the three hydrogen bonds to Asn67, Lys134 and a water molecule (which is hydrogen bonded to OD1 Asn204 and OE1 Gln64). Secondly, there is a shift in the position of Gln64, with the NE2 atom forming a hydrogen bond to O Ala348, instead of the OE1 interacting with a water which is hydrogen bonded between O Thr65 and ND2 Asn67 (as seen in the other structures). These probably play a far lesser contribution to the weaker *K*_i_ values than the absence of three potential hydrogen bonds from a C-2-OH group.

Another key ligand-protein interaction is displayed for compounds **1**, **16**, **17** and **31**, which feature hydrogen bonding interactions from their amine groups at ring position 1 to Glu205 (2.6–2.8 Å) and with a water which is also hydrogen bonded to ND2 Asn135, and in the case of **1**, to the C-4 hydroxyl group. While there is no equivalent group for **14** and **22**, the Glu205 side-chain is oriented similarly in all of the structures despite the lack of hydrogen bonds with a C-2 hydroxyl group for **14** as well as **22** and **31**. The position of Glu205 may be maintained by an additional hydrogen bond from its OE1 atom to a water which is also hydrogen-bonded to OH Tyr209. 

In contrast to the carbacyclic compounds, **14** and **22** have a nitrogen atom in position 1 of the ring. The sugar conformation is close to “^4^C_1_” (=^4^C_N_) in both, and the N of the isofagomine moiety is hydrogen bonded to Glu205 and Glu349, which may compensate for the lack of interactions from an NH group at the start of the hydrocarbon chain seen for compounds **1**, **16**, **17** and **31**. The absence of a C-2-position hydroxyl group will increase the basicity of the N atom, allowing it to also form electrostatic interactions with Glu349. In the structure with **22** the N atom points more steeply downwards away from the plane of the ring forming a closer hydrogen bond with OE1 Glu349 than for **14** (2.5–2.6 Å for all of the molecules in the asymmetric unit, as opposed to 2.6–2.8 Å for **14**). This may contribute to the better inhibition observed for **22** than for **14** with all the galactosidases tested. 

In **1**, which is known to be a powerful β-galactosidase inhibitor, the sugar conformation is closer to ^4^H_3_, probably because of the double bond between C-5 and C-5a, and reflecting that the C in ring position 2 is not attracted downwards towards the catalytic Glu residues in the same way as for the isofagomine N atom in **14** and **22**.

**Figure 6:**
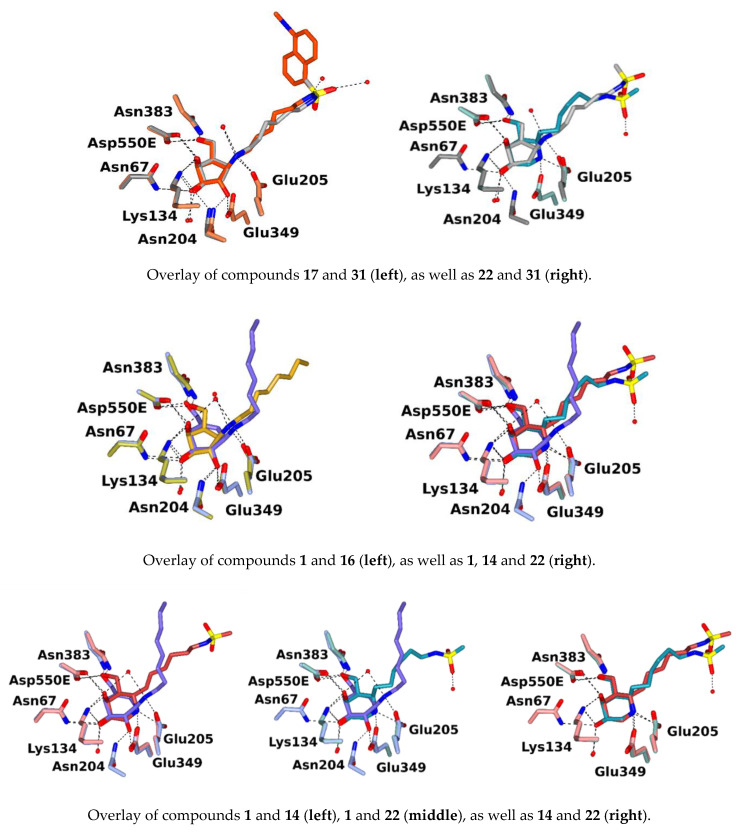
Overlay of inhibitors.

Overlays of the compared ligand structures and active sites are shown in Figure 6.

### 2.5. Chaperoning Activities

**Figure 7:**
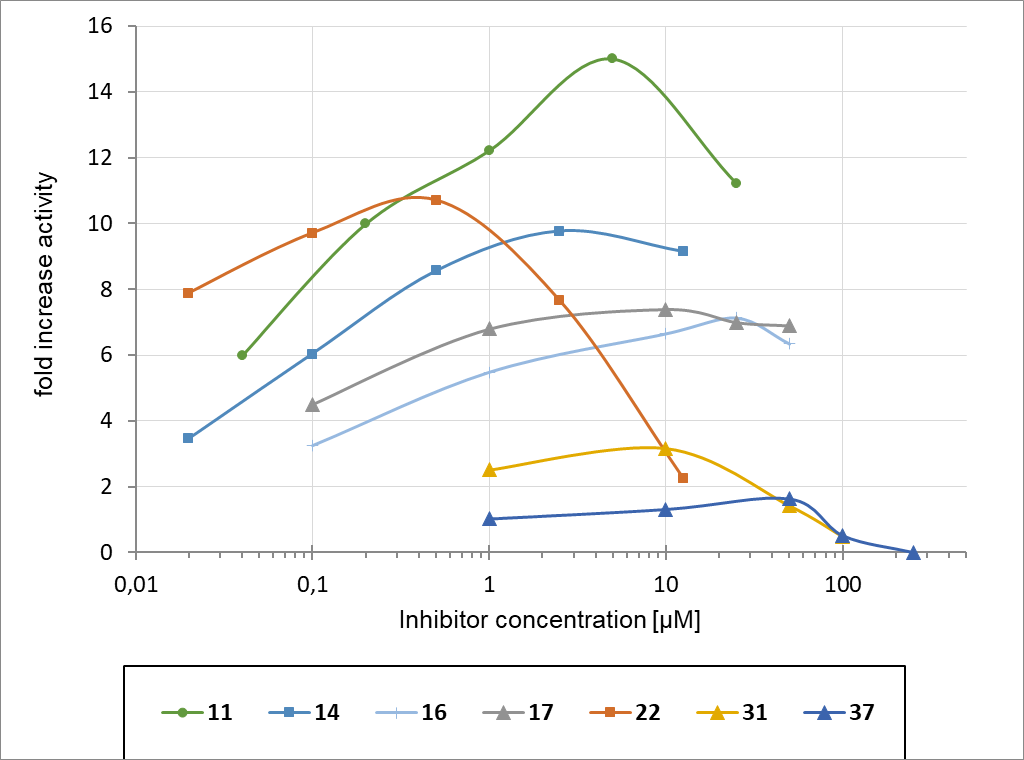
Activity enhancements of R201C mutant lysosomal β-galactosidase with compounds **11**, **14**, **16**, **17**, **22**, **31** and **37**.

**Figure 8:**
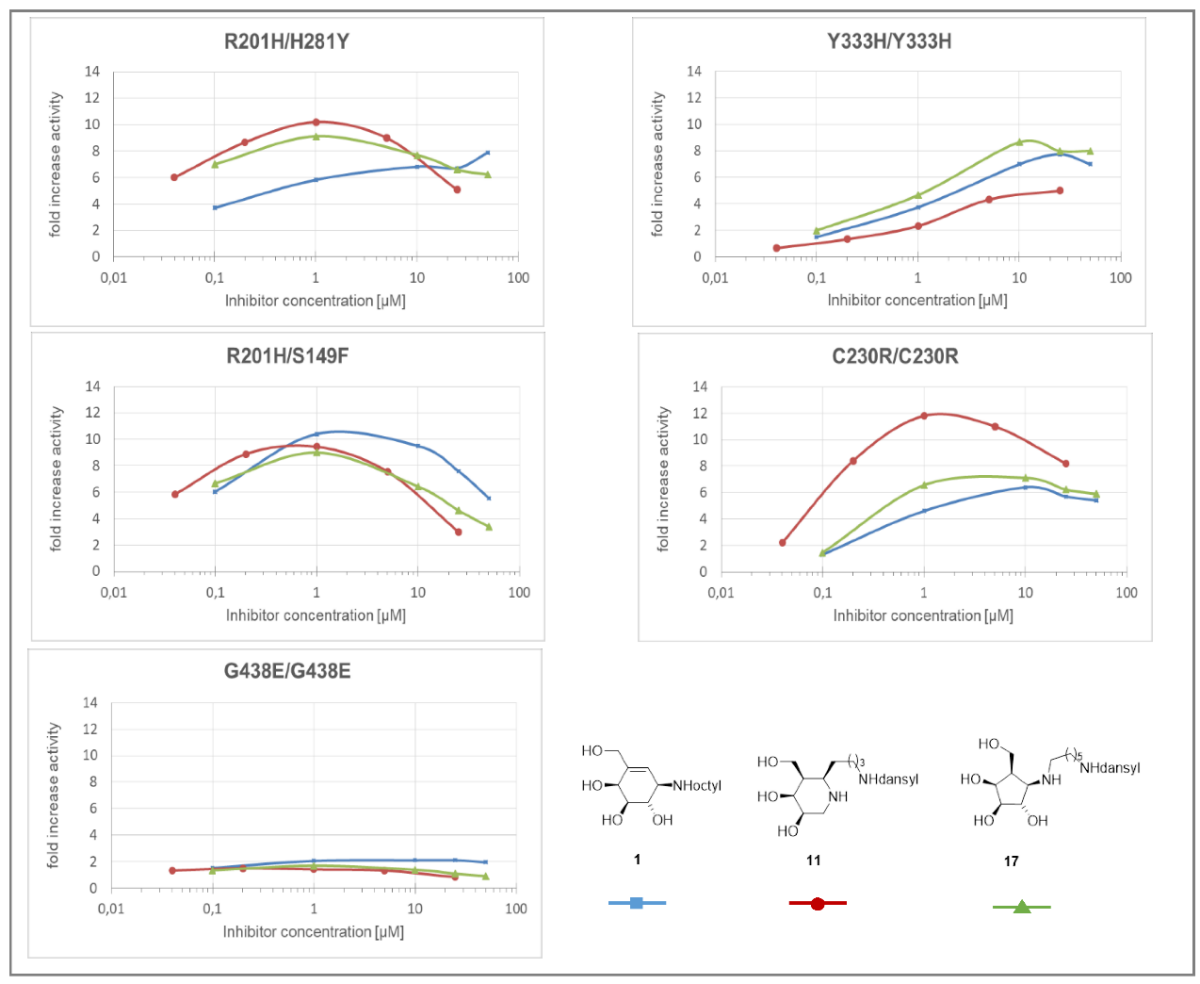
Activity enhancements of various lysosomal β-galactosidase mutants with compounds **1**, **11**, **17**.

Chaperoning activities of compounds with G_M1_-gangliosidosis and Morquio B related cell lines are outlined in Figure 7 and Figure 8. Cells with the R201C mutant of β-galactosidase which is related to juvenile G_M1_-gangliosidosis and has previously been shown to be amenable to chaperone **1** reacted very positively to isofagomine derivatives **11**, **14** and **22** as well as to cyclopentanoid ligands **16** and **17**. The corresponding deoxy derivative **31** exhibited distinctly reduced chaperoning activity and was only trailed by deoxyfluoro analog **37**. Incubation of the cells with compounds **31** and **37**, respectively, up to 10 µM had no notable influence on β-galactosidase activity and cell viability. Higher concentrations of these inhibitors led to decreased enzyme activity and to a complete arrest of cell growth over the incubation time. Concentrations of more than 100 µM resulted in dramatic loss of both, β-galactosidase activity as well as cell viability. 

With R201H/H281Y (adult G_M1_-gangliosidosis), both structural types, **11** as well as **17**, show β-gal activity increases of 6- to 7-fold at 0.1 µM and 9- to 10-fold at 1 µM with slight advantages for isofagomine derivative **11**. For comparison, the maximum effect of carbasugar **1** (6.8-fold increase) is clearly shifted to higher chaperone concentrations of 10–25 µM (Figure 7).

With C230R/C230R (late infantile G_M1_-gangliosidosis), chaperoning maxima were reached at 1 µM with 4-*epi*-isofagomine **11** (12-fold) and between 1 and 10 µM (7-fold) with carbacycle **17** (NOEV, **1**, 6.4-fold). The Morquio B/G_M1_-gangliosidosis mutation R201H/S149F exhibits comparable sensitivities to all three chaperones screened, with small advantage for NOEV (**1**, ≥10-fold), followed by **11** (9.5-fold) and **17** (9-fold). Interestingly, with Y333H/Y333H (juvenile G_M1_-gangliosidosis), carbacycle **17** is the most potent chaperone with a maximum activity of nearly 9-fold at 10 µM, followed by NOEV (**1**, nearly 8-fold at 25 µM) and **11** (5-fold at 25 µM). None of the three chaperones probed showed any chaperone activity with Y270D/Y270D and G438E/G438E, respectively. The compounds had no inhibitory effects on human lysosomal hexosaminidase in the concentration range probed (Figure 8).

## 3. Discussion

Two types of structurally very different but similarly potent β-galactosidase inhibitors, the C-5-chain extended 4-*epi*-isofagomines (**14** and **22**) as well as d-*galacto* configured aminohydroxymethyl-cyclopentane derivatives **17**, **31** and **37** were investigated concerning their properties as pharmacological chaperones for selected G_M1_-gangliosidosis as well as Morquio B cell lines. To gain more in-depth insights into structural requirements for good inhibition and chaperoning, an important hydroxyl group in the cyclopentanoid series was substituted. Removal of this secondary alcohol was expected to provide isofagomine-like improved activity through increase of the amine’s basicity whereas its substitution with a fluorine atom was exploited to probe hydrogen bonding with the amino acid residues in and around the active site. 

New inhibitor **22** turned out to be highly potent and exhibited encouraging dose-dependent chaperoning profiles with enzyme mutant R201C. For the cyclopentanoid chaperones, on the other hand, OH-2 appears to be a vital feature for their activity as inhibitors and pharmacological chaperones, since its absence and the concomitantly increased basicity of the adjacent amine apparently cannot compensate for the reduced number of interactions with amino acid residues resulting from the removal of this particularly crucial functional group.

Notably, despite indisputable biochemical evidence based on quite a few inhibitors probed [41,46,47,55,61,62,63,64], the frequently observed synergistic inhibitory effect of the terminal dansyl amide could not be correlated to additional specific binding events of this moiety to the protein as was implied by the lack of electron density for this substituent in the bacterial model enzyme-inhibitor complexes examined.

Furthermore, it is interesting to note that the five β-galactosidase mutants investigated (Figure 8) exhibit distinctly different susceptibilities towards the three classes of chaperones screened. Whereas for R201H/H281Y and, in particular, C230R/C230R the isoimino sugars (for example **11**) are clearly the most efficient chaperones across the entire concentration range probed, cyclopentane **17** shows clear advantage over pyranoid carbasugar **1** as well as piperidine **11** with Y333H/Y333H, and in the case of R201H/S149F, carbasugar NOEV (**1**), turned out to be the most potent chaperone for this particular mutant. Thus, for the development of effective pharmacological chaperones, the individual mutant under scrutiny should be screened with a representative range of suitable ligand types for optimized chaperoning efficacy.

## 4. Experimental

### 4.1. Synthetic Section

XRD data and NMR spectra for new compounds are all in the Supplementary Materials. Optical rotations were measured at 20 °C on a Perkin Elmer 341 polarimeter (Perkin Elmer, Waltham, MA, USA) at a wavelength of 589 nm and a path length of 10 cm. NMR spectra were recorded on a Varian INOVA 500 (Varian, Palo Alto, CA, USA) operating at 499.82 MHz (^1^H), 470.3 MHz (^19^F) and at 125.894 MHz (^13^C) or on a Bruker (Billerica, MA, USA) Ultrashield spectrometer at 300.36 (^1^H) and 75.53 (^13^C) MHz, respectively. CDCl_3_ was employed for protected compounds and methanol-*d*_4_ or D_2_O for unprotected inhibitors. Carbon and hydrogen numbering in NMR spectra was conducted in analogy to carbohydrate nomenclature and clockwise, starting with the amino bearing carbon as C-1 (Numbering pattern for cyclopentanes shown in Figure 2). Chemical shifts are listed in delta employing residual, non-deuterated solvent as the internal standard. Signals were assigned unambiguously by COSY, HSQC as well as APT analysis. For the sake of simplicity and comparison, carbohydrate numbering has been applied to the hydroxymethyl cyclopentane series throughout this work. This may be accounted for by the fact that these compounds can be regarded “monosaccharide derivatives” resulting from formal homolytic extrusion of the ring oxygen. Stereochemical features remain the same as in the respective parent sugar. The signals of the aromatic groups are located in the expected regions and are not listed explicitly. The signals of the protecting groups as well as of the *N*-substituents were found in the expected regions and are only listed explicitly when overlapping with important spectral features of the respective compound. Due to stable rotameric populations of the *N*-Boc group, signal splitting in the respective NMR spectra has been observed leading to somewhat poor resolution of the ^1^H-NMR and ^13^C-NMR spectra. Signals of *N*-Boc protected compounds may be retarded or show up as pairs. For crucial intermediate **33**, the structure was confirmed by XRD structural analysis: Suitable single crystals of compounds were immersed in silicone oil, mounted using a glass fiber and frozen in the cold nitrogen stream (100 K). X-Ray diffraction data were collected at low temperature on a Bruker Kappa APEX II diffractometer using Mo K_a_ radiation (l = 0.71073 Å) generated by an INCOATEC micro-focus source. The data reduction and absorption correction was performed with the Bruker SMART (Bruker AXS Inc., Madison, WI, USA) and Bruker SADABS (Bruker AXS Inc., Madison, Wisconsin, USA), respectively. The structures were solved with SHELXT [65] by direct methods and refined with SHELXL by least-square minimization against *F*^2^ using first isotropic and later anisotropic thermal parameters for all non-hydrogen atoms. Hydrogen atoms were added to the structure models on calculated positions using the riding model. The space group assignments and structural solutions were evaluated using PLATON [66,67].

MALDI-TOF Mass Spectrometry was performed on a Micromass TofSpec 2E Time-of-Flight Mass Spectrometer (Waters Corporation, Milford, MA, USA). Analytical TLC was performed on precoated aluminum plates silica gel 60 F254 (E. Merck 5554, E. Merck, Darmstadt, Germany) and detected with UV light (254 nm). For staining, a solution of vanillin (9 g) in a mixture of H_2_O (950 mL)/EtOH (750 mL)/H_2_SO_4_ (120 mL) or ceric ammonium molybdate (100 g ammonium molybdate/8 g ceric sulfate in 10% H_2_SO_4_ (1 L)) were employed followed by heating on a hotplate. For column chromatography, silica gel 60 (230–400 mesh, E. Merck 9385) or silica gel 60 (Acros Organics, AC 24036, Thermo Fisher Scientific Inc., Waltham, MA, USA) were used.

**6-[(5a*R*)-(*N*-*tert*-Butyloxycarbonyl-3,4-*O*-isopropylidene-6-*O*-methoxymethylene-4-*epi*-isofagomin-5a-yl)-hex-4-enoic nitrile (19)**

A solution of freshly prepared LDA [by dropwise addition of 2.5 M *n*-BuLi (2.0 mL) in hexane to diisopropyl amine (0.18 mL, 1.3 mmol, 20% solution in dry THF)] in dry THF was added dropwise at −78 °C under an atmosphere of nitrogen to a suspension of triphenyl-(3-cyano)propylphosphonium bromide (557 mg, 1.36 mmol). After stirring at −40°C for 60 min, a solution of aldehyde **18** (ref. [46,47], 150 mg, 0.39 mmol, dissolved in 2 mL dry THF) was added dropwise. After having been stirred for 12 h, the reaction mixture was diluted with CH_2_Cl_2_ and consecutively washed with HCl (6%) and saturated NaHCO_3_. After drying over Na_2_SO_4_, the filtrate was concentrated under reduced pressure to provide the crude product. Chromatographic purification (cyclohexane–EtOAc 20:1 to cyclohexane–EtOAc 10:1) afforded nitrile **19** (110 mg, 0.25 mmol, 66%) as a mixture of the *E*/*Z*-isomers. ^1^H NMR (300 MHz, CD_3_OD): δ = 5.40 (bs, 2 H, H-2′, H-3′), 4.63–4.55 (m, 2 H, O-CH_2_-O-CH_3_), 4.47–3.80 (m, 4 H, H-2eq, H-3, H-4, H-5a), 3.60 (m, 2 H, H-6a, H-6b), 3.33–3.28 (m, 3 H, O-CH_2_-O-CH_3_), 2.62 (m, 2 H, H-2ax, H-1′a), 2.45–2.01 (m, 6 H, H-5, H-1′b, H-4′a, H-4′b, H-5′a, H-5′b), 1.47 (s, 3 H, C(CH_3_)_2_), 1.38 (s, 9 H, N-CO-C(CH_3_)_3_), 1.27 (s, 3 H, C(CH_3_)_2_); ^13^C NMR (75.5 MHz, CD_3_OD): δ = 154.7, 154.4 (N-CO-C(CH_3_)_3_), 129.7–126.7 (C-2′, C-3′), 119.1, 119.0 (C-6′), 109.2, 109.2 (C(CH_3_)_2_), 96.6, 96.5 (O-CH_2_-O-CH_3_), 80.6, 79.9 (N-CO-C(CH_3_)_3_), 73.0, 72.9, 71.0, 70.4 (C-3, C-4), 66.3, 65.9 (C-6), 55.4, 55.3 (O-CH_2_-O-CH_3_), 50.5, 49.3 (C-5a), 40.2, 39.8, 39.3, 38.9 (C-2, C-5, C-4′), 29.7, 29.8, 28.7, 28.3, 25.9 (N-CO-C(CH_3_)_3_, C(CH_3_)_2_), 23.6, 23.3, 17.4 (C-1′, C-4′, C-5′). MS (MALDI): Calcd for [C_22_H_36_N_2_O_6_Na]^+^: *m*/*z* 447.2471 [M]^+^; Found [M]^+^ 447.2468.

**(5a*R*)-*N*-*tert*-Butyloxycarbonyl-5a-*C*-(6-amino)hexyl-3,4-*O*-isopropylidene-6-methoxymethylene-4-*epi*-isofagomine (20)**

A 10% solution of the nitrile **19** (110 mg, 0.25 mmol) in MeOH was stirred with Raney-Ni (moist in H_2_O) under an atmosphere of H_2_ at ambient pressure for 60 min. After completed conversion of the starting material, the catalyst was filtered off. Removal of the solvent, followed by purification on silica gel (CHCl_3_–MeOH 8:1 + 1 vol% NH_4_OH (25%)) provided amine **20** (92 mg, 0.21 mmol, 84%) as a yellow syrup. ${\left[\alpha \right]}_{D}^{20}$: −1.2 (*c* 2.6, MeOH); ^1^H NMR (300 MHz, CD_3_OD): δ = 4.66–4.63 (m, 2 H, O-CH_2_-O-CH_3_), 4.32 (m, 2 H, H-4, H-5a), 4.13–3.91 (m, 2 H, H-2eq, H-4), 3.39–3.35 (m, 3 H, O-CH_2_-O-CH_3_), 3.68 (m, 2 H, H-6a, H-6b), 2.82 (t, 2 H, H-6′), 2.81 (m, 1 H, H-2ax), 2.32 (m, 1 H, H-5), 2.06 (m, 1 H, H-1′a), 1.69–1.16 (m, 24 H, H-1′b, H-2′, H-3′, H-4′, H-5′, C(CH_3_)_3_, N-CO-C(CH_3_)_3_). ^13^C NMR (75.5 MHz, CD_3_OD): δ = 156.7, 156.5 (N-CO-C(CH_3_)_3_), 110.5 (C(CH_3_)_2_), 97.8, 97.6 (O-CH_2_-O-CH_3_), 81.5, 81.3 (N-CO-C(CH_3_)_3_), 74.5, 72.2, 72.0 (C-3, C-4), 67.5, 67.3 (C-6), 55.7 (O-CH_2_-O-CH_3_), 52.2, 50.8 (C-5a), 41.9, 41.4, 41.1, 40.7, 39.9 (C-2, C-5, C-6′), 32.2–26.2 (C-1′, C-2′, C-3′, C-4′, C-5′, N-CO-C(CH_3_)_3_, C(CH_3_)_2_). MS (MALDI): Calcd for [C_22_H_42_N_2_O_6_Na]^+^: *m*/*z* 453.2941 [M]^+^; Found [M]^+^ 453.2940.

**(5a*R*)-*N*-*tert*-Butyloxycarbonyl-5a-*C*-(6-dansylamino)hexyl-3,4-*O*-isopropylidene-6-*O*-methoxymethylene-4-*epi*-isofagomine (21)**

To a suspension of amine **20** (92 mg, 0.21 mmol) and Na_2_CO_3_ (68 mg, 0.64 mmol) in 3 mL MeOH, dansyl chloride (69 mg, 0.26 mmol) was added. After completed conversion of the starting material, the reaction mixture was evaporated to dryness. Purification on silica gel (cyclohexane–EtOAc 15:1) gave partially protected dansylamide **21** (86 mg, 0.13 mmol, 61%) as fluorescent wax. ${\left[\alpha \right]}_{D}^{20}$: +0.7 (*c* 1.0, CHCl_3_); ^1^H NMR (300 MHz, CDCl_3_): δ = 4.70–4.56 (m, 2 H, O-CH_2_-O-CH_3_), 4.26–3.81 (m, 4 H, H-2eq, H-3, H-4, H-5a), 3.55 (m, 2 H, H-6a, H-6b), 3.41–3.32 (m, 3 H, O-CH_2_-O-CH_3_), 2.78 (m, 2 H, H-6′), 2.52 (m, 1 H, H-2ax), 2.18 (m, 1 H, H-5), 1.78 (m, 1 H, H-1′a), 1.46 (s, 3 H, (C(CH_3_)_2_), 1.43 (s, 9 H, N-CO-C(CH_3_)_3_), 1.30 (s, 3 H, (C(CH_3_)_2_), 1.28–0.94 (m, 9 H, H-1′b, H-2′, H-3′, H-4′, H-5′). ^13^C NMR (75.5 MHz, CDCl_3_): δ = 155.0, 154.8 (N-CO-C(CH_3_)_3_), 109.3 (C(CH_3_)_2_), 96.7, 96.6 (O-CH_2_-O-CH_3_), 80.0, 79.9 (N-CO-C(CH_3_)_3_), 73.2, 73.1, 71.1, 70.9 (C-3, C-4), 66.5, 66.1 (C-6), 55.5, 55.4 (O-CH_2_-O-CH_3_), 50.8, 49.5 (C-5a), 45.6 (dansyl), 43.5, 43.4 (C-6′), 40.1, 39.5, 38.8 (C-2, C-5), 29.8, 29.6 (C-1′), 28.8, 26.5 (C(CH_3_)_2_), 28.7, 28.6 (N-CO-C(CH_3_)_3_), 27.1–26.2 (C-2′, C-3′, C-4′, C-5′). MS (MALDI): Calcd for [C_34_H_53_N_3_O_8_SNa]^+^: *m*/*z* 686.3451 [M]^+^; Found [M]^+^ 686.3450.

**(5a*R*)-5a-*C*-(6-Dansylamino)hexyl-4-*epi*-isofagomine (22)**

Compound **21** (86 mg, 0.13 mmol) was dissolved in MeOH (3 mL) and HCl (12 M) was added dropwise to adjust pH 1. After completed removal of all protecting groups, the solvents were removed under reduced pressure. Purification on silica gel (CHCl_3_–MeOH 8:1 + 1 vol% NH_4_OH (25%)) yielded free compound **22** (46 mg, 0.096 mmol, 74%) as fluorescent wax. ${\left[\alpha \right]}_{D}^{20}$: −19.1 (*c* 0.58, MeOH); ^1^H NMR (300 MHz, CD_3_OD): δ = 3.85 (m, 3 H, H-3, H-4, H-6a), 3.70 (dd, 1 H, *J*_6a,6b_ 11.8 Hz, *J*_5,6b_ 4.5 Hz, H-6b), 3.21 (m, 1 H, H-5a), 3.12 (dd, 1 H, *J*_2ax,2eq_ 12.3 Hz, *J*_2eq,3_ 2.9 Hz, H-2eq), 3.07 (dd, 1 H, *J*_2ax,3_ 2.3 Hz, H-2ax), 3.07 (t, 2 H, H-6′), 2.01 (m, 1 H, H-5), 1.66 (m, 2 H, H-1′), 1.34–0.96 (m, 8 H, H-2′, H-3′, H-4′, H-5′). ^13^C NMR (75.5 MHz, CD_3_OD): δ = 69.3 (C-4), 66.7 (C-3), 58.6 (C-5a), 57.4 (C-6), 45.8 (dansyl), 43.7 (C-6′), 42.3 (C-5), 30.4, 30.1, 29.6, 27.1, 26.5 (C-1′, C-2′, C-3′, C-4′, C-5′). MS (MALDI): Calcd for [C_24_H_37_N_3_O_5_SH]^+^: *m*/*z* 480.2532 [M]^+^; Found [M]^+^ 480.2531.

**(3a*R*,3b*S*,6a*S*,7*S*,7a*R*)-1-Benzyl-7-bromo-5,5-dimethylhexahydro-1*H*[1,3]dioxolo[4′5′:3,4]cyclopenta[1,2-*c*]isoxazole (25)**

To an ice-cooled solution of alcohol **23** (ref. [57], 292 mg, 1.00 mmol) in dichloromethane (10 mL), pyridine (0.324 mL, 4.01 mmol) and trifluoromethanesulfonyl anhydride (0.219 mL, 1.30 mmol) were added and the reaction was allowed to reach ambient temp under stirring. After completed conversion of the starting material, the reaction mixture was diluted with dichloromethane and consecutively washed with aqueous HCl (6%) and saturated aqueous sodium bicarbonate. After drying (Na_2_SO_4_), the filtrate was concentrated under reduced pressure. Resulting crude triflate **24** was dissolved in DMF (10 mL) and NaBr (516 mg, 5.01 mmol) was added and the mixture was stirred at ambient temp for 12 h. The reaction mixture was then concentrated under reduced pressure, the residue was treated with CH_2_Cl_2_ and the resulting suspension was washed with brine. The organic layer was dried (Na_2_SO_4_), filtered and solvents were removed. Purification of the remaining residue on silica gel (cyclohexane–EtOAc 10:1) provided bromodeoxy compound **25** (187 mg, 0.528 mmol, 53%, over 2 steps) as a colorless syrup. ${\left[\alpha \right]}_{D}^{20}$: +11.8 (*c* 1.0, CHCl_3_); ^1^H NMR (500 MHz, CDCl_3_); Mixture of conformers: δ = 4.71–4.54 (m, 2 H, H-3, H-4), 4.30 (m, 1 H, *J*_5,6a_ 4.2 Hz, *J*_5,6a_ 8.5 Hz, H-6a), 4.08–4.01 (m, 2 H, H-6b, N-CH-Ph), 3.98 (dd, 1 H, *J*_1,2_ = *J*_2,3_ 7.0 Hz, H-2), 3.91–3.75 (m, 2 H, H-1, N-CH-Ph), 3.38–3.28 (m, 1 H, H-5), 1.54, 1.32 (2 s, 3 H each, C(CH_3_)_2_). ^13^C NMR (125.9 MHz, CDCl_3_): δ = 113.5, 113.4 (C(CH_3_)_2_), 88.7, 88.2 (C-3), 79.4, 78.9 (C-1), 78.5, 78.1 (C-4), 65.0 (C-6), 59.8, 59.7 (N-CH_2_-Ph), 54.8 (C-2), 47.4, 47.1 (C-5), 27.5, 27.4, 25.6, 25.6 (C(CH_3_)_2_). MS (MALDI): Calcd for [C_16_H_20_BrNO_3_H]: *m*/*z* 357.0705 [M]^+^; Found [M]^+^ 357.0708.

**[(3a*S*,4*R*,5*S*,6a*R*)-5-Amino-2,3-dimethyltetrahydro-4*H*-cyclopenta[*d*][1,3]dioxol-4-yl]methanol (26)**

A 10% methanolic solution of bromo compound **25** (187 mg, 0.528 mmol) was stirred with Raney-Ni under an atmosphere of H_2_ at ambient pressure for 12 h. After completed conversion of the starting material, the catalyst was removed by filtration, the filtrate was concentrated under reduced pressure and the remaining residue was chromatographically purified (CHCl_3_–MeOH 8:1 + 1 vol% aq. NH_4_OH (25%)) to provide aminocyclopentane **26** (65.4 mg, 0.349 mmol, 66%) as a pale yellow syrup. ${\left[\alpha \right]}_{D}^{20}$: −3.2 (*c* = 1.0, MeOH); ^1^H NMR (500 MHz, CD_3_OD) δ = 4.80 (dd, 1 H, *J*_2,3_ = *J*_3,4_ 5.8 Hz, H-3), 4.73 (dd, 1 H, *J*_4,5_ 5.7 Hz, H-4), 3.98 (dd, 1 H, *J*_5,6a_ 7.0 Hz, *J*_6a,6b_ 11.1 Hz, H-6a), 3.94 (dd, 1 H, *J*_5,6b_ 8.1 Hz, H-6b), 3.39 (m, 1 H, H-1), 2.10–2.02 (m, 2 H, H-2a, H-5), 2.01 (ddd, 1 H, *J*_2b,3_ 5.8 Hz, *J*_2a,2b_ 11.8 Hz, H-2b), 1.52, 1.37 (C(CH_3_)_2_. ^13^C NMR (125.9 MHz, CD_3_OD) δ = 110.8 (C(CH_3_)_2_), 82.4 (C-3), 82.3 (C-4), 59.0 (C-6), 56.0 (C-1), 51.2 (C-5), 40.7 (C-2), 26.9, 23.0 (C(CH_3_)_2_). MS (MALDI): Calcd for [C_9_H_17_NO_3_H]: *m*/*z* 188.1287 [M+H]^+^; Found [M+H]^+^ 188.1287.

**(1*R*,2*S*,3*R*,4*S*)-4-Amino-3-(hydroxymethyl)cyclopentane-1,2-diol (27)**

A solution of isopropylidene acetal **26** (15.8 mg, 84.4 µmol) in methanol (0.5 mL) was treated with aqueous HCl (2 M, 0.5 mL) at ambient temperature. After completed conversion was observed solvents were removed under reduced pressure and the remaining residue was purified on silica gel (CHCl_3_–MeOH–NH_4_OH (25%). 8:4:1) to give free polyol **27** as the free base (10.2 mg, 69.3 mmol, 82%). ${\left[\alpha \right]}_{D}^{20}$: +18.5 (*c* 0.86, H_2_O, pH 1); ^1^H NMR (500 MHz, CD_3_OD) δ = 4.15 (dd, 1 H, *J*_2b,3_ 4.4 Hz, *J*_3,4_ 3.9 Hz, *J*_2a,3_ 8.4 Hz, H-3), 4.08 (dd, 1 H, *J*_4,5_ 3.8 Hz, H-4), 3.93 (dd, 1 H, *J*_5,6a_ 7.3 Hz, *J*_6a,6b_ 11.2 Hz, H-6a), 3.83 (dd, 1 H, *J*_5,6b_ 7.8 Hz, H-6b), 3.61 (ddd, 1 H, *J*_1,2a_ 8 Hz, *J*_1,2b_ 4.4 Hz, *J*_1,5_ 4 Hz, H-1), 2.59 (ddd, 1 H, *J*_2a,3_ 8.3 Hz, *J*_2a,2b_ 14.3 Hz, *J*_1,2a_ 8 Hz, H-2a), 2.20 (dddd, 1 H, H-5), 1.58 (ddd, 1 H, *J*_1,2b_ 4.4 Hz, *J*_2b,3_ 8 Hz, H-2b). ^13^C NMR (125.9 MHz, CD_3_OD) δ = 73.1 (C-4), 72.1 (C-3), 57.2 (C-6), 50.1 (C-1), 45.3 (C-5), 37.3 (C-2). MS (MALDI): Calcd for [C_9_H_17_NO_3_H]: *m*/*z* 148.0974 [M + H]^+^; Found [M+H]^+^ 148.0976.

**6-[(3a*S*,4*R*,5*S*,6a*R*)-4-Hydroxymethyl-2,2-dimethyltetrahydro-4*H*-cyclopenta[*d*][1,3]dioxol-5-yl)amino]hexanoic nitrile (28)**

Intermediate **26** (25.4 mg, 0.136 mmol) was dissolved in DMF (1 mL), 6-bromohexanoic nitrile (23.4 µL, 0.176 mmol) and NaHCO_3_ (45.6 mg, 0.705 mmol) were added and the mixture was stirred at 60 °C until quantitative conversion of the starting material was observed. The solvent was removed under reduced pressure and the remaining residue was purified by chromatography on silica gel (CHCl_3_–MeOH 15:1 + 1 vol% NH_4_OH (25%)) to yield cyanopentylamine **28** (23.6 mg, 62%) as a faintly yellow syrup. ${\left[\alpha \right]}_{D}^{20}$: +18.1 (*c* 1.0, CHCl_3_); ^1^H-NMR (300 MHz, CDCl_3_): δ = 4.68 (dd, 1 H, *J*_2,3_ = *J*_3,4_ 6 Hz, H-3), 4.57 (dd, 1 H, *J*_4,5_ 6 Hz, H-4), 4.05 (dd, 1 H, *J*_5,6a_ 7.7 Hz, *J*_6a,6b_ 11.7 Hz, H-6a), 3.93 (dd, 1 H, *J*_5,6b_ 6.7 Hz, H-6b), 3.21 (dd, 1 H, *J*_1,2b_ = *J*_1,5_ 5.9 Hz, H-1), 2.85–2.70 (m, 2 H, H-1′a, NH), 2.43–2.26 (m, 3 H, H-1′b, H-5′), 2.23–2.01 (m, 2 H, H-2a, H-5), 1.73–1.38 (m, 8 H, H-2b, H-2′, H-3′, H-4′, 6-OH), 1.45, 1.28 (2 s, 3H each, C(CH_3_)_2_). ^13^C-NMR (75.5 MHz, CDCl_3_): δ = 119.7 (CN), 110.1 (C(CH_3_)_2_), 81.2, 81.1 (C-3, C-4), 61.3 (C-1), 59.7 (C-6), 47.8 (C-5), 47.4 (C-1′), 34.8 (C-2), 29.6, 26.8, 26.6, 25.4, 23,2, 17.2 (C-2′, C-3′, C-4′, C-5′, C(CH_3_)_2_). MS (MALDI): Calcd for [C_15_H_26_N_2_O_3_H]: *m*/*z* 283.2022 [M + H]^+^; Found [M + H]^+^ 283.2020.

**(3a*S*,4*R*,5*S*,6a*R*)-5-(6-aminohexyl)amino-2,2-dimethyltetrahydro-4*H*-cyclopenta[*d*][1,3]dioxol-4-yl]methanol (29)**

A 10% methanolic solution of nitrile **28** was stirred with Raney-Ni under an atmosphere of H_2_ at ambient pressure and temperature until completed consumption of the starting material (30 min). The catalyst was then removed by filtration, the filtrate was concentrated under reduced pressure and the remaining residue was chromatographed on silica gel (CHCl_3_–MeOH–NH_4_OH (25%) 8:4:1) providing diamine **29** (18.5 mg, 78%) as a pale yellow syrup. ${\left[\alpha \right]}_{D}^{20}$: −3.4 (*c* 0.82, MeOH); ^1^H-NMR (300 MHz, CDCl_3_): δ = 4.68 (dd, 1 H, *J*_2,3_ = *J*_3,4_ 6.0 Hz, H-3), 4.57 (dd, 1 H, *J*_4,5_ 5.8 Hz, H-4), 4.05 (dd, 1 H, *J*_5,6a_ 7.6 Hz, *J*_6a,6b_ 11.6 Hz, H-6a), 3.93 (dd, 1 H, *J*_5,6b_ 6.4 Hz, H-6b), 3.22 (dd, 1 H, *J*_1,2b_ = *J*_1,5_ 6.0 Hz, H-1), 3.10 (bs, 3 H, 1-NH, 6′-NH_2_), 2.72 (m, 3 H, H-1′a, H-6′), 2.37 (m, 1 H, H-1′b), 2.22–2.01 (m, 2 H, H-2a, H-5), 1.67–1.54 (m, 1 H, H-2b), 1.53–1.22 (m, 15 H, H-2′, H-3′, H-4′, H-5′, 6-OH, C(CH_3_)_2_). ^13^C-NMR (75.5 MHz, CDCl_3_): δ = 110.1 (C(CH_3_)_2_), 81.3, 81.0 (C-3, C-4), 61.3 (C-1), 59.6 (C-6), 47.7, 47.6 (C-5, C-1′), 41.8 (C-6′), 34.9 (C-2), 32.6, 30.2, 27.2, 26.7, 26.7, 23.2 (C-2′, C-3′, C-4′, C-5′, C(CH_3_)_2_). MS (MALDI): Calcd for [C_15_H_30_N_2_O_3_H]: *m*/*z* 287.2335 [M + H]^+^; Found [M + H]^+^ 287.2332.

**(3a*S*,4*R*,5*S*,6a*R*)-2,2-Dimethyl-5-[6-(dansylamino)hexyl]aminotetrahydro-4*H*-cyclopenta[*d*][1,3]dioxol-4-yl)methanol (30)**

A solution of diamine **29** (18.2 mg, 63.5 µmol) in CH_2_Cl_2_–DMF (1 mL, 3:1) was treated with trimethylamine (35.4 µL, 254 µmol) and dansyl chloride (18.9 mg, 69.9 µmol). After completed conversion of the starting material (20 min), the solvents were removed under reduced pressure and the residue was chromatographed on silica gel (CHCl_3_–MeOH 15:1 + 1 vol% NH_4_OH (25%)) furnishing dansyl amide **30** (27.6 mg, 48.6 µmol, 84%) as a yellow syrup. ${\left[\alpha \right]}_{D}^{20}$: −1.4 (*c* 1.1, MeOH); ^1^H-NMR (300 MHz, CDCl_3_): δ = 4.98 (bs, 1 H, NH-dansyl), 4.67 (dd, 1 H, *J*_2b,3_ = *J*_3,4_ 6.0 Hz, H-3), 4.56 (dd, 1 H, *J*_4,5_ 5.8 Hz, H-4), 4.05 (dd, 1 H, *J*_5,6a_ 7.6 Hz, *J*_6a,6b_ 11.4 Hz, H-6a), 3.94 (dd, 1 H, *J*_5,6b_ 6.5 Hz, H-6b), 3.19 (m, 1 H, *J*_1,2b_ = *J*_1,5_ 5.8 Hz, H-1), 2.91–2.82 (m, 8 H, H-6′, dansyl), 2.70–2.59 (m, 1 H, H-1′a), 2.34–2.23 (m, 1 H, H-1′b), 2.19–1.98 (m, 2 H, H-2a, H-5), 1.60 (ddd, 1 H, *J*_1,2b_ 14.9 Hz, H-2b), 1.43, 1.27 (2 s, 3H each, C(CH_3_)_2_), 1.41–1.06 (m, 5 H, H-2′, H-3′, H-4′, H-5′). ^13^C-NMR (75.5 MHz, CDCl_3_): δ = 110.1 (C(CH_3_)_2_), 81.2, 81.0 (C-4, C-3), 61.4 (C-1), 59.7 (C-6), 47.5, 47.4 (C-5, C-1′), 45.5 (dansyl), 43.2 (C-6′), 34.9 (C-2), 29.8, 29.6, 26.7, 26.6, 26.1, 23.2 (C-2′, C-3′, C-4′, C-5′, C(CH_3_)_2_). MS (MALDI): Calcd for [C_27_H_41_N_3_O_5_SH]: *m*/*z* 520.2845 [M + H]^+^; Found [M + H]^+^ 520.2848.

**(1*R*,2*S*,3*R*,4*S*)-3-Hydroxymethyl-4-(6′-dansylaminohexylamino)cyclopentane-1,2-diol (31)**

A solution of isopropylidene acetal **30** (27.4 mg, 527 µmol) in THF (0.5 mL) was treated with aqueous HCl (1 M, 0.5 mL) at ambient temperature. Solvents were removed under reduced pressure and the remaining residue was purified on silica gel (CHCl_3_–MeOH 8:1 + 1 vol% NH_4_OH (25%)) to give free carbasugar **31** as the free base (18.5 mg, 38.6 µmol, 73%). ${\left[\alpha \right]}_{D}^{20}$: +17.6 (*c* 0.80, MeOH, free base); ^1^H-NMR (300 MHz, CDCl_3_): δ = 4.04 (ddd, 1 H, *J*_2a,3_ 7.9 Hz, *J*_2b,3_ = *J*_3,4_ 4.3 Hz, H-3), 3.96 (dd, 1 H, *J*_4,5_ 4.4 Hz, H-4), 3.93–3.86 (m, 2 H, H-6a, H-6b), 3.39 (ddd, 1 H, *J*_1,2b_ = *J*_1,5_ 5.4 Hz, H-1), 2.93–2.80 (m, 8 H, H-6′, dansyl), 2.79–2.54 (m, 2 H, H-1′), 2.37 (ddd, 1 H, *J*_1,2a_ = *J*_2a,3_ 7.9 Hz, *J*_2a,2b_ 14.2 Hz, H-2a), 2.23 (dddd, 1 H, *J*_5,6a_ = *J*_5,6b_ 7 Hz, H-5), 1.69 (m, 1 H, H-2b), 1.49–1.04 (m, 8 H, H-2′, H-3′, H-4′, H-5′). ^13^C-NMR (75.5 MHz, CDCl_3_): δ = 74.5 (C-4), 73.2 (C-3), 58.8 (C-6), 58.6 (C-1), 48.4 (C-1′), 47.0 (C-5), 45.8 (dansyl), 43.7 (C-6′), 37.4 (C-2), 30.4, 28.4, 27.3, 27.1 (C-2′, C-3′, C-4′, C-5′). MS (MALDI): Calcd for [C_24_H_37_N_3_O_5_SH]: *m*/*z* 480.2532 [M + H]^+^; Found [M + H]^+^ 480.2533.

**(3a*R*,3b*S*,6a*S*,7*S*,7a*R*)-1-Benzyl-7-fluoro-5,5-dimethylhexahydro-1*H*-[1,3]dioxolo[4′,5′:3,4]cyclopenta[1,2-*c*]isoxazole (32)**

A solution of alcohol **23** (ref. [57], 298 mg, 1.02 mmol) in CH_2_Cl_2_ (10 mL) was cooled to 0 °C and treated with pyridine (0.330 mL, 4.09 mmol) and DAST (0.270 mL, 2.05 mmol). When completed conversion of the starting material was observed (2 h), the reaction mixture was quenched with saturated aqueous NaHCO_3_ and the organic layer was dried with Na_2_SO_4_, filtered and the solvent was removed under reduced pressure. Purification of the residue on silica gel (cyclohexane-EtOAc 10:1) provided deoxyfluoro compound **32** (245 mg, 0.835 mmol, 82%). ${\left[\alpha \right]}_{D}^{20}$: +55.5 (*c* 1.6, CHCl_3_); ^1^H NMR (500 MHz, CDCl_3_): δ = 4.78 (m, 1 H, *J*_2,F_ 50.7 Hz, H-2), 4.60 (m, 1 H, H-4), 4.58 (m, 1 H, *J*_3,F_ 19 Hz, *J*_2,3_ 6.5 Hz, *J*_3,4_ 3.7 Hz, H-3), 4.20 (dd, 1 H, *J*_5,6a_ 3.7 Hz, *J*_6a,6b_ 8.6 Hz, H-6a), 3.98–3.89 (m, 2 H, *J*_5,6b_ 8.5 Hz, H-6b, N-CH-Ph), 3.76 (d, 1H, N-CH-Ph) 3.71 (m, 1 H, *J*_1,F_ 20 Hz, *J*_1,2_ = *J*_1,5_ 4 Hz, H-1), 3.30 (m, 1 H, H-5), 1.46, 1.24 (2 s, 3 H each, C(CH_3_)_2_). ^13^C NMR (75.5 MHz, CDCl_3_): δ = 113.0 (C(CH_3_)_2_), 100.3 (*J*_2,F_ 182 Hz, C-2), 85.3 (*J*_2,F_ 27.9 Hz, C-3), 77.7 (*J*_4,F_ 4.7 Hz, C-4), 75.9 (*J*_1,F_ 25.1 Hz, C-1), 65.0 (C-6), 59.8 (N-CH_2_-Ph), 47.5, (*J*_5,F_ 3.5 Hz, C-5), 26.9, 25.3 (C(CH_3_)_2_). ^19^F NMR (470.3 MHz, CDCl_3_): δ = −189.4. MS (MALDI): Calcd for [C_16_H_20_FNO_3_H]: *m*/*z* 294.1505 [M + H]^+^; Found [M + H]^+^ 294.1504.

**[(3a*S*,4*R*,5*R*,6*S*,6a*S*)-5-Amino-6-fluoro-2,2-dimethyltetrahydro-4*H*-cyclopenta[*d*][1,3]dioxol-4-yl]methanol (33)**

A methanolic solution (3%) of isoxazolidine **32** (348 mg, 1.18 mmol) was stirred with Pearlman’s catalyst (Pd(OH)_2_/C, 20%) under an atmosphere of H_2_ at ambient pressure for 1 h. After completed conversion, the suspension was filtered, the filtrate was concentrated under reduced pressure and the residue was chromatographically purified (CHCl_3_–MeOH 14:1 + 1 vol% NH_4_OH (25%)) to obtain **33** as a pale yellow syrup (238 mg, 1.16 mmol, 98 %). ${\left[\alpha \right]}_{D}^{20}$: +22.5 (*c* 0.70, CHCl_3_); ^1^H NMR (500 MHz, CDCl_3_): δ = 4.73 (dd, 1 H, *J*_3,4_ = *J*_4,5_ 5.4 Hz, H-2), 4.72 (bd, 1 H, *J*_2,F_ 48 Hz, H-2), 4.60 (ddd, 1 H, *J*_3,F_ 16.6 Hz, *J*_2,3_ 5.8 Hz, H-3), 4.01 (dd, 1 H, *J*_5,6a_ 7.7 Hz, *J*_6a,6b_ 11.3 Hz, H-6a), 3.95 (dd, 1 H, *J*_5,6b_ 6.8 Hz, H-6b), 3.47 (dd, 1 H, *J*_1,F_ 14.4 Hz, *J*_1,5_ 6.3 Hz, H-1), 2.44 (ddd, 1 H, H-5), 2.07 (bs, 3 H, 6-OH, 1-NH_2_), 1.45, 1.29 (2 s, 3 H each, C(CH_3_)_2_). ^13^C NMR (75.5 MHz, CDCl_3_): δ = 111.3 (C(CH_3_)_2_), 100.5 (*J*_2,F_ 180.5 Hz, C-2), 84.1 (*J*_3,F_ 33.7 Hz, C-3), 80.9 (C-4), 58.1 (*J*_1,F_ 22.4 Hz, C-1), 58.0 (C-6), 46.4, (C-5), 26.3, 25.0 (C(CH_3_)_2_). ^19^F NMR (470.3 MHz, CDCl_3_): δ = −181.1. MS (MALDI): Calcd for [C_9_H_16_NO_3_H]: *m*/*z* 206.1192 [M + H]^+^; Found [M + H]^+^ 206.1192.

**(1*S*,2*S*,3*S*,4*R*,5*R*)-4-Amino-3-fluoro-5-hydroxymethylcyclopentane-1,2-diol (34)**

A 10% methanolic solution of compound **33** (238 mg, 1.16 mmol) was treated with HCl (12 M, 0.1 mL) for 2 h. After completed conversion of the starting material, the solution was concentrated under reduced pressure and the residue was chromatographically purified (CHCl_3_–MeOH–NH_4_OH (25%) 8:4:1) to obtain free compound **34** (177 mg, 1.07 mmol, 92 %) as a colorless syrup. Treatment with HCl_g_ in methanol in the presence of small amounts of ethyl acetate afforded the corresponding hydrochloride (**34**∙HCl) as a white solid. ${\left[\alpha \right]}_{D}^{20}$: +31.2 (free base, *c* 0.88, H_2_O); ^1^H NMR (500 MHz, D_2_O) (free base): δ = 5.17 (ddd, 1 H, *J*_2,F_ 52.5 Hz, *J*_1,2_ 3.9 Hz, *J*_2,3_ 7.0 Hz, H-2), 4.32 (dd, 1 H, *J*_3,F_ = 21.7 Hz, *J*_3,4_ 3.9 Hz, H-3), 4.27 (dd, 1 H, *J*_4,5_ 4 Hz, H-4), 4.01 (ddd, 1 H, *J*_1,F_ 20.8 Hz, *J*_1,5_ 8.8 Hz, H-1), 3.99 (dd, 1 H, *J*_5,6a_ 6.6 Hz, *J*_6a,6b_ 11.7 Hz, H-6a), 3.91 (dd, 1 H, *J*_5,6b_ 8.0 Hz, H-6b), 2.79 (dddd, 1 H, H-5). ^13^C NMR (75.5 MHz, D_2_O) (free base): δ= 101.6 (*J*_2,F_ 185.9 Hz, C-2), 76.1 (*J*_3,F_ 22.0 Hz, C-3), 72.2 (*J*_4,F_ 9.1 Hz, C-4), 56.6 (C-6), 54.4 (*J*_1,F_ 26.8 Hz, C-1), 41.8, (*J*_5,F_ 2.7 Hz, C-5). ^19^F NMR (470.3 MHz, D_2_O) (free base): δ = −192.4. ^1^H NMR (300 MHz, D_2_O) (hydrochloride): δ = 5.12 (ddd, 1 H, *J*_2,F_ 52.3 Hz, *J*_1,2_ 3.9 Hz, *J*_2,3_ 6.6 Hz, H-2), 4.26 (dd, 1 H, *J*_3,F_ = 21.3 Hz, *J*_3,4_ 4.1 Hz, H-3), 4.20 (dd, 1 H, *J*_4,5_ 3.8 Hz, H-4), 3.97 (ddd, 1 H, *J*_1,F_ 20.9 Hz, *J*_1,5_ 8.8 Hz, H-1), 3.92 (dd, 1 H, *J*_5,6a_ 6.9 Hz, *J*_6a,6b_ 11.5 Hz, H-6a), 3.83 (dd, 1 H, *J*_5,6b_ 8.2 Hz, H-6b), 2.74 (dddd, 1 H, H-5). ^13^C NMR (75.5 MHz, D_2_O) (hydrochloride): δ = 100.9 (*J*_2,F_ 186.3 Hz, C-2), 76.1 (*J*_3,F_ 22.0 Hz, C-3), 72.1 (*J*_4,F_ 9.1 Hz, C-4), 56.5 (C-6), 54.4 (*J*_1,F_ 27.3 Hz, C-1), 41.5, (*J*_5,F_ 2.5 Hz, C-5). MS (MALDI): Calcd for [C_6_H_12_FNO_3_H]: *m*/*z* 166.0880 [M + H]^+^; Found [M + H]^+^ 166.0883.

**Benzyl 6-(1*R*,2*S*,3*S*,4*S*,5*R*)-2-fluoro-3,4-dihydroxy-5-(hydroxymethyl)cyclopentylamino hexylcarbamate (35)**

To a 10% solution of amine **34** (117 mg, 0.708 mmol) and acetic acid (40 µL), benzyl (6-oxohexyl) carbamate [68] (230 mg, 0.921 mmol) was added. After 5 min, sodium cyanoborohydrate (66.8 mg, 1.06 mmol) was added and the suspension was stirred until completed conversion was observed (1 h). The solvent was removed under reduced pressure and the residue was chromatographically purified (CHCl_3_–MeOH 8:1 + 1 vol% NH_4_OH (25%)) to obtain compound **35** as a colorless syrup (169 mg, 0.424 mmol, 60%). ${\left[\alpha \right]}_{D}^{20}$: +16.8 (*c* 1.1, MeOH); ^1^H NMR (500 MHz, CD_3_OD): δ = 5.13 (s, 2 H, NH(C-O)-O-CH_2_-Ph), 4.85 (ddd, 1 H, *J*_2,F_ 53.4 Hz, *J*_1,2_ = *J*_2,3_ 4.6 Hz, H-2), 4.12 (dd, 1 H, *J*_3,4_ = *J*_3,4_ 4.4 Hz, H-4), 4.05 (ddd, 1 H, *J*_3,F_ 22.2 Hz, H-3), 3.94 (dd, 1 H, *J*_5,6a_ 6.8 Hz, *J*_6a,6b_ 11.4 Hz, H-6a), 3.84 (dd, 1 H, *J*_5,6b_ 6.6 Hz, H-6b), 3.24 (ddd, 1 H, *J*_1,F_ 21.8 Hz, *J*_1,5_ 8.4 Hz, H-1), 3.18 (t, 2 H, *J* 6.9 Hz, H-6′), 2.81 (m, 1 H, H-1′a), 2.60 (m, 1 H, H-1′b), 2.46 (m, 1 H, H-5), 1.63–1.34 (m, 8 H, H-2′, H-3′, H-4′, H-5′). ^13^C NMR (75.5 MHz, CD_3_OD) (free base): δ = 158.9 (NH(C-O)-O-CH_2_-Ph), 106.0 (*J*_2,F_ 185.0 Hz, C-2), 78.5 (*J*_3,F_ 23.1 Hz, C-3), 73.6 (*J*_4,F_ 8.3 Hz, C-4), 67.3 (N(C-O)-O-CH_2_-Ph), 63.2 (*J*_1,F_ 22.4 Hz, C-1), 58.7 (C-6), 45.9, (*J*_5,F_ 3.8 Hz, C-5), 41.7 (C-6′), 30.8, 30.5, 28.0, 27.6 (C-2′, C-3′, C-4′, C-5′). ^19^F NMR (470.3 MHz, CD_3_OD): δ = −184.3. MS (MALDI): Calcd for [C_6_H_12_FNO_3_H]: *m*/*z* 399.2295 [M+H]^+^; Found [M+H]^+^ 399.2295.

**(1*S*,2*S*,3*S*,4*R*,5*R*)-4-(6′-Dansylaminohexyl)amino-3-fluoro-5-hydroxymethylcyclopentane-1,2-diol (37)**

To a solution of carbamate **35** (52.5 mg, 0.132 mmol) in MeOH, 10% Pd/C was added and the suspension was stirred under an atmosphere of H_2_ at ambient pressure. After completed conversion was observed (1 h), the suspension was filtered and the filtrate was concentrated under reduced pressure. The residue of crude free amine **36** was dissolved in CH_3_CN/H_2_O (1 mL, 5:1) and treated with Et_3_N (55.1 µL, 0.395 mmol) and dansyl chloride (39.1 mg, 0.145 mmol). After completed conversion of the starting material (20 min), the solvent was removed under reduced pressure. Purification on silica gel (CHCl_3_–MeOH. 8:1 + 1 vol% NH_4_OH (25%)) provided compound **37** (38.2 mg, 69.2 µmol, 53%, over 2 steps) as yellow syrup. ${\left[\alpha \right]}_{D}^{20}$: +14.0 (*c* 1.8, MeOH); ^1^H NMR (500 MHz, CD_3_OD): δ = 4.83 (ddd, 1 H, *J*_2,F_ 53.4 Hz, *J*_1,2_ 4.0 Hz, *J*_2,3_ 4.4 Hz, H-2), 4.12 (dd, 1 H, *J*_3,4_ = *J*_3,4_ 4.6 Hz, H-4), 4.05 (ddd, 1 H, *J*_3,F_ 22.2 Hz, H-3), 3.92 (dd, 1 H, *J*_5,6a_ 6.8 Hz, *J*_6a,6b_ 11.2 Hz, H-6a), 3.82 (dd, 1 H, *J*_5,6b_ 6.5 Hz, H-6b), 3.20 (ddd, 1 H, *J*_1,F_ 21.7 Hz, *J*_1,5_ 8.3 Hz, H-1), 2.96–2.88 (m, 8 H, H-6′, dansyl), 2.67 (m, 1 H, H-1′a), 2.49–2.41 (m, 2 H, H-5, H-1′b), 1.42–1.06 (m, 8 H, H-2′, H-3′, H-4′, H-5′). ^13^C NMR (75.5 MHz, CD_3_OD): δ = 105.6 (*J*_2,F_ 185.1 Hz, C-2), 78.5 (*J*_3,F_ 23.1 Hz, C-3), 73.6 (*J*_4,F_ 8.3 Hz, C-4), 63.2 (*J*_1,F_ 22.4 Hz, C-1), 58.7 (C-6), 48.6 (C-1′), 45.9, (*J*_5,F_ 4.1 Hz, C-5), 45.8 (dansyl), 43.7 (C-6′), 30.4, 30.3, 27.7, 27.3 (C-2′, C-3′, C-4′, C-5′). ^19^F NMR (470.3 MHz, CD_3_OD): δ = −184.4. MS (MALDI): Calcd for [C_6_H_12_FNO_3_H]: *m*/*z* 498.2438 [M+H]^+^; Found [M+H]^+^ 498.2439.

### 4.2. Kinetic Studies

Kinetic studies were performed at 37 °C in an appropriate buffer (specific conditions depicted below). In a typical assay, the enzyme was incubated with different inhibitor concentrations for up to 5 min before initiating the reaction by the addition of substrate. The initial reaction rate was measured by monitoring the increase in absorbance at 400 nm for up to ten minutes using a Cary4000 (Varian, Palo Alto, CA, USA). *K*_i_ determinations were performed using at least two different substrate concentrations. For each inhibitor, a range of four to six inhibitor concentrations bracketing the ultimately determined *K*_i_ value was used for each substrate concentration. Dixon plots (1/v vs. [I]) were constructed to validate the use of competitive inhibition model. The data were then fit using non-linear regression analysis with Grafit 7.0 [Erithacus Software, UK].

n.i. stands for no inhibition or weak inhibition with an estimated *K*_i_ value higher than 1 mM.

#### Specific Assay Conditions for Each Enzyme

***Agrobacterium* sp. β-glucosidase:** [69,70] 50 mM sodium phosphate buffer (pH 7). Substrate: *p*NP β-Gal, *K*_m_ = 4.1 mM.

***E. coli* lac *z* β-galactosidase:** 50 mM sodium phosphate, 1.0 mM MgCl_2_ (pH 7). Substrate: *p*NP β-Gal, *K*_m_ = 60 µM.

***Bovine liver* β-galactosidase:** 50 mM sodium phosphate buffer (pH 7). Substrate: *p*NP β-Gal, *K*_m_ = 0.65 mM.

**Fabrazyme (Acid α-galactosidase):** 20 mM sodium citrate, 50 mM sodium phosphate, 1.0 mM tetrasodium EDTA, 0.25% *v*/*v* Triton X-100^®^ and 0.25% *w*/*v* taurocholic acid buffer (pH 5.5). Substrate: 2,4-DNP α-Gal [71], *K*_m_ = 0.65 mM.

**GCase (β-glucocerebrosidase):** 20 mM sodium citrate, 50 mM sodium phosphate, 1.0 mM tetrasodium EDTA, 0.25% *v*/*v* Triton X-100^®^ and 0.25% *w*/*v* taurocholic acid buffer (pH 7). Substrate: 2,4-DNP β-Glc [71], *K*_m_ = 2.7 mM.

***S. cerevisiae* α-Glucosidase:** 50 mM sodium phosphate buffer (pH 7.0). Substrate: *p*NP α-Glc, *K*_m_ = 0.75 mM.

**Human β-galactosidase** and **β-hexosaminidase** activity measurements were performed in duplicate assays, unless otherwise stated. Fibroblast cells were harvested by trypsinization in 0.9% NaCl containing 0.01% Triton, homogenized by sonication (3 × 10 s, Sonifier Bandelin Sonopuls, Bandelin, Berlin, Germany) and centrifuged at 13,000 rpm for 2 min in a table top centrifuge (Biofuge Pico, Heraeus, Hanau, Germany). Protein amounts were determined according to the method of Lowry [72].

For assessment of β-Gal activity, 20 µL of cell homogenate were mixed with 100 µL of 0.5 mM 4-methylumbelliferyl-β-d-galactopyranoside (Sigma-Aldrich, St. Louis, MO, USA), in 100 mM citrate buffer (pH 4.0) containing 100 mM NaCl and 0.02% NaN_3_. After incubation at 37 °C for 30 min, the reaction was stopped by adding 2.5 mL 400 mM glycine/NaOH (pH 10.4).

β-Hexosaminidase activity was measured by adding 10 µL homogenate to 90 µL 0.9% NaCl and 100 µL of 1 mM 4-methylumbelliferyl-*N*-acetyl-β-d-glucosaminide (Sigma-Aldrich, St. Louis, MO, USA) in 100 mM citrate buffer (pH 4.6) containing 0.2% BSA and 0.04% NaN_3_. The reaction was stopped after 10 min at 37 °C by addition of 2.5 mL of 400 mM glycine/NaOH (pH 10.4). The amount of hydrolyzed 4-methylumbelliferone was determined with a fluorescence spectrometer Hitachi F7000 (Chiyoda, Japan). All compounds probed were found practically devoid of β-hexosaminidase inhibitory activity.

Modified β-Gal assays were used to estimate the half maximal inhibitory concentration (IC_50_) of the particular chaperone. For IC_50_ determination, 0.001 to 100 µM of chaperone was added to the assay mixture. Activity was measured in normal fibroblasts. Data analysis was performed with Microcal™ Origin^®^ v6.0 (Origin Lab, Northampton, MA, USA) using a non-linear curve fitting module based on sigmoid curve fitting.

### 4.3. CjGH35 Purification and Crystallisation

*Cj*GH35 was expressed and purified as in Larsbrink [73]. Crystals were grown by Sitting Drop Vapour Diffusion using protein at 25–36 mg/mL in 25 mM HEPES pH 7.0, 100 mM NaCl, in ratios of 0.8–1.2 µL to 1 µL well solution, with the latter comprised of 2.3–2.8 M sodium acetate pH 7.4–7.6. Crystals were soaked with a speck of solid ligand powder, introduced into the drop on the end of a needle, for 1 to 3 days, and frozen directly into liquid nitrogen using Hampton CryoLoops^TM^ (Hampton, CA, USA).

### 4.4. Data Collection and Structure Refinement

Data were collected at the Diamond Light Source on beamlines IO4 (**1**, **16** and **17**), IO4-1 (**31**) and IO2 (**14** and **22**), and processed using *DIALS* (**1**, **16**, **17**, **22** and **31**) [74] or XDS (**14**) [75]. All data were scaled with *AIMLESS* [76] to resolutions of 1.46 to 1.60 Å, and were in space group P1. The structures were solved by molecular replacement using *REFMAC* (for **1**, **16**, **17** and **22**) [77] or *PHASER* (**31** and **14**) [78] using the structure of 4D1I.pdb without water molecules and ions as the model. The datasets were in space group P1, with 8 molecules in the asymmetric unit. The structures were refined using *REFMAC*, with twin refinement using observed intensities converted to amplitudes employed in the case of **14**, and anisotropic *B*-factor refinement employed for all of the structures. Manual rebuilding was performed using *Coot* [79] followed by cycles of refinement, and the ligand molecules were added into 2*mF_o_-DF_c_* density maps calculated after the protein chains had been modelled and most of the water molecules and sodium ions added. The crystallographic programs were run within the *CCP4I2* suite [80].

### 4.5. Patients and Cell Lines

Cell lines from one G_M1_-Gangliosidosis-patient (R201C) and one WT, were exposed to compounds **22**, **31**, and **37**, for evaluation of their chaperone effects.

Human skin fibroblasts were grown in minimal essential medium (MEM) with Earle’s Salts (Sigma Aldrich, St. Louis, MO, USA) containing 10% fetal bovine serum, 400 µM l-glutamine, and 50 µg/mL gentamycin at 37 °C and 5% CO_2_. All cells used in this study were between the third and nineteenth passages.

Potential chaperones were dissolved in DMSO and diluted in 10 mM phosphate buffer (pH 7.0) containing 100 mM NaCl, 0.01% NaN_3_, and 0.01% Triton for the IC_50_-measurements and in MEM for the *in-vivo* tests. The total DMSO concentration in the media was less than 1% and had no effect on β-Gal activity or cell viability.

### 4.6. Treatment of Cultured Fibroblasts

Fibroblasts were grown to semi-confluency in 6-well plates. Particular chaperone was added to the culture medium at following concentrations: 100–0.02 µmol. Cells were incubated for four additional days at 37 °C. Cells were harvested by scraping and prepared for β-galactosidase and β-hexosaminidase assays as described above.

## Figures and Tables

**Table 1:** *K*_i_-values [µM] of compounds with Abg = β-glucosidase/β-galactosidase from *Agrobacterium* sp.; *E. coli* = *lac Z* β-galactosidase from *E. coli*; Bovine liver = β-galactosidase from bovine liver; Fabrazyme = commercial recombinant lysosomal α-galactosidase; *S. cer.* = α-glucosidase from *S. cerevisiae*; GCase = recombinant human lysosomal β-glucocerebrosidase; β-Gal. human lys. = human lysosomal acid β-galactosidase; IC_50_ [µM] with β-Gal (human lysosomal); n.i. = no inhibition, with *K*_i_ > 1 mM.

Enzyme (GH Family)	Compounds						
	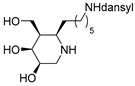 22	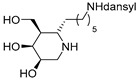 14 [46,47]	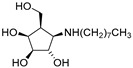 16	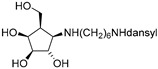 17 [55]	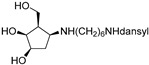 31	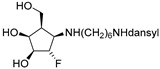 37	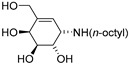 1
Abg (β-Glc/Gal) (GH1)	0.0003	0.0175	0.0010	0.0012	0.0035	0.077	7.7
*E. coli* (β-Gal) (GH2)	0.0004	0.0021	0.0132	0.0101	0.0041	5.2	2.8
Bovine liver (β-Gal) (GH35)	0.0005	0.0014	0.1167	0.0053	0.102	0.137	0.87 [16]
Fabrazyme (α-Gal) (GH27)	n.i.	n.i.	n.i.	238.1	n.i.	n.i.	n.i.
*S. cer.* (α-Glc) (GH13)	n.i.	n.i.	n.i.	n.i.	n.i.	22.5	n.i.
GCase (β-Glc) (GH30)	0.003	1.2	0.0639	0.0193	22.6	3.0	83
β-Gal human lys. (GH35); IC_50_	0.094	0.38	0.42	0.40	82.5	34.9	0.51

## Supplementary Materials

The following are available online at. XRD data and NMR spectra for new compounds.
