# Supplementary material for: Mechanistic Insights into the Chaperoning of Human Lysosomal-Galactosidase Activity: Highly Functionalized Aminocyclopentanes and C-5a-Substituted Derivatives of 4-epi-Isofagomine
Source: Molecules. 2020 Sep 3;25(17):4025. doi: 10.3390/molecules25174025 (PMC7504770; doi:10.3390/molecules25174025)
Supplement: Supplementary file 1 [file molecules-25-04025-s001.pdf]

Supplementary Materials:

Mechanistic Insights into the Chaperoning of Human Lysosomal-Galactosidase Activity: Highly Functionalized Aminocyclopentanes and *C*-5a-Substituted Derivatives of 4-*epi*-Isofagomine

Patrick Weber [1,+], Martin Thonhofer [1,+], Summer Averill [1], Gideon J. Davies [2], Andres Gonzalez Santana [3], Seyed A. Nasseri [3], Wendy A. Offen [2], Bettina M. Pabst [4], Eduard Paschke [4], Michael Schalli [1], Ana Torvisco [5], Marion Tschernutter [4], Christina Tysoe [3], Werner Windischhofer [4], Stephen G. Withers [3], Andreas Wolfsgruber [1], Tanja M. Wrodnigg [1] and Arnold E. Stütz [1,*]

[1] Glycogroup, Institute of Chemistry and Technology of Biobased Systems, Graz University of Technology, Stremayrgasse 9, A-8010 Graz Austria; stuetz@tugraz.at

[2] Department of Chemistry, University of York, Heslington, York YO10 5DD, North Yorkshire, United Kingdom; gideon.davies@york.ac.uk

[3] Chemistry Department, University of British Columbia, 2036 Main Mall, Vancouver BC, Canada V6T 1Z1; withers@chem.ubc.ca

[4] Institute of Inorganic Chemistry, Graz University of Technology, Stremayrgasse 9, A-8010 Graz Austria; ana.torviscogomez@tugraz.at

[5] Laboratory of Metabolic Diseases, Department of Pediatrics, MedUni Graz, Auenbruggerplatz 30, A-8036 Graz, Austria; werner.windischhofer@medunigraz.at

+ Authors contributed equally.

* Correspondence: stuetz@tugraz.at; Tel.: +43-316-873-32079

Supplementary data: XRD data, ^{1}H NMR, ^{13}C NMR, HSQC, COSY and ^{19}F NMR spectra

Table 1. XRD data of Enzyme-inhibitor complexes.

	*Cj*GH35-1	*Cj*GH35-14	*Cj*GH35-16	*Cj*GH35-17	*Cj*GH35-22	*Cj*GH35-31
**Data collection**						
Space group	*P1*	*P1*	*P1*	*P1*	*P1*	*P1*
Cell dimensions						
a, *b*, *c* (Å)	99.1, 115.9, 116.1	99.4, 115.8, 116.2	99.4, 115.6, 115.9	99.3, 115.7, 116.0	99.0, 116.0, 115.6	99.1, 115.4, 115.7
α, β, γ (°)	90.2, 90.0, 90.1	89.9, 90.1, 90.0	90.3, 90.0, 90.2	90.1, 90.0, 90.0	89.8, 90.1, 90.0	90.2, 90.0, 89.8
Resolution (Å)	116.10-1.46 (1.48-1.46)	63.21-1.50 (1.53-1.50)	115.94-1.50 (1.53-1.50)	115.98-1.50 (1.53-1.50)	63.16-1.60 (1.63-1.60)	81.86-1.50 (1.53-1.50)
Total no. of reflections	2979048	1425840	2751115	2761219	1376212	1700636
No. unique reflections	865389	793061	798809	798304	650269	789757
R_{merge}	0.059(0.645)	0.079 (0.372)	0.050(0.654)	0.049(0.773)	0.058 (0.497)	0.077 (0.601)
R_{pim}	0.037(0.432)	0.079 (0.372)	0.031(0.407)	0.031 (0.480)	0.046 (0.417)	0.065 (0.508)
$CC_{1/2}$	0.996(0.662)	0.982 (0.585)	0.998 (0.693)	0.998 (0.644)	0.995 (0.678)	0.986 (0.604)
$I / \sigma I$	9.6 (1.6)	4.9 (1.7)	11.1 (1.8)	11.0 (1.5)	4.6 (0.8)	5.9 (1.2)
Completeness (%)	96.4 (94.1)	95.6 (94.0)	96.6 (94.5)	96.5 (94.4)	95.8 (93.5)	96.2 (94.3)
Redundancy	3.4 (3.1)	1.8 (1.8)	3.4 (3.5)	3.5 (3.5)	2.1 (2.1)	2.2 (2.2)
**Refinement**						
No. reflections working set	821933	752547	758576	758165	617754	749920
No. reflections test set	43369	40511	40201	40094	32511	39666
R_{work} / R_{free}	0.13/0.17	0.14/0.16	0.13/0.18	0.14/0.18	0.15/0.21	0.13/0.18
No. atoms						
Protein	34634	33237	34583	34606	33484	34361
Ligand/ion	256	160	243	316	157	216
Water	5125	2095	4590	4495	2502	3978
B-factors (Å^2)						
Protein	23.7	21.8	25.3	26.3	29.1	24.0
Ligand/ion	26.2	22.0	25.5	27.8	28.9	25.1
Water	37.2	26.2	36.8	37.1	33.6	33.0
R.m.s. deviations						
Bond lengths (Å)	0.017	0.014	0.017	0.007	0.017	0.017
Bond angles (°)	1.979	1.837	1.968	1.444	1.942	1.960
Ramachandran plot residues						
In most favorable regions (%)	95.7	95.6	95.8	96.0	95.9	95.9
In allowed regions (%)	3.2	3.4	3.1	3.0	3.0	3.0
**PDB code**	6TBI	6TBJ	6TBF	6TBG	6TBK	6TBH

Single Crystal X-ray Crystallography

All crystals suitable for single crystal X-ray diffractometry were removed from a vial or a Schlenk and immediately covered with a layer of silicone oil. A single crystal was selected, mounted on a glass rod on a copper pin, and placed in the cold N_2 stream provided by an Oxford Cryosystems cryostream. XRD data collection was performed for compound **33**, on a Bruker APEX II diffractometer[1] with use of an IμS microsource (Incoatec microfocus) sealed tube of Mo Kα radiation (λ= 0.71073 Å) and a CCD area detector. Data integration was carried out using SAINT [1]. Empirical absorption corrections were applied using SADABS [2-3]. The structures were solved with use of the intrinsic phasing option in SHELXT[4] and refined by the full-matrix least-squares procedures in SHELXL [4-8] as implemented in the program SHELXLE [9]. The space group assignments and structural solutions were evaluated using PLATON [10-12]. The hydrogen atoms bonded to N-1 and O1 were located in the difference map. Other hydrogen atoms were positioned geometrically and refined using a riding model with C—H = 0.95–1.00 Å and with Uiso(H) = 1.2 (1.5 for methyl groups) times Ueq(C). Due to insufficient anomalous dispersion effects, the absolute structure was not established in this analysis but according to the configuration of the starting materials. All crystal structures representations were made with the program Diamond [13]. CIF files were edited, validated and formatted either with the programs encipher [14] and publCIF [15]. CCDC 2018297contain the supplementary crystallographic data for compound **33** respectively. These data can be obtained free of charge from The Cambridge Crystallographic Data Centre *via* www.ccdc.cam.ac.uk/data_request/cif. Table **2** contains crystallographic data and details of measurements and refinement for compound **33**.

Table 2. Crystallographic data and details of measurements for compound **33.**
Mo Kα (λ=0.71073Å). $R1= \Sigma/ |F_o|- |F_c|/|\Sigma|F_d$; $wR2 = [\Sigma_w(F_o^2-F_2^2)^2/\Sigma_w(F_o^2)^2]^{1/2}$

**Compound**	**AT749**
Formula	$C_9H_{16}FNO_3$
Fw (g mol^{-1})	205.23
a (Å)	6.3214(7)
b (Å)	12.1708(13)
c (Å)	13.1888(14)
α (°)	90
β (°)	90
γ (°)	90
V (Å^3)	1014.70(19)
Z	4
Crystal size (mm)	0.09 × 0.08 × 0.06
Crystal habit	Block, colourless
Crystal system	Orthorhombic
Space group	$P2_12_12_1$
d_{calc} (Mg m^{-3})	1.343
μ (mm^{-1})	0.11
T (K)	100(2)
2*θ* range (°)	2.3–24.7
F(000)	440
T_{min}, T_{max}	0.554, 0.747
R_{int}	0.092
No. of measured, independent and observed [*I* > 2s(*I*)] reflections	28012, 1788, 1605
independent reflections	1788
No. of parameters, restraints	139, 0
$\Delta›_{max}$, $\Delta›_{min}$ (e Å^{-3})	0.16, -0.14
R1, wR2 (all data)	R1 = 0.0380<br>wR2 = 0.0789
R1, wR2 (>2*σ*)	R1 = 0.0315<br>wR2 = 0.0752

6-[(5a*R*)-(*N-tert*-Butyloxycarbonyl-3,4-*O*-isopropylidene-6-*O*-methoxymethylene-4-*epi*-isofagomin-5a-yl)-hex-4-enoic nitrile (19)

^{1}H NMR (300 MHz, $CDCl_3$): Compound **19**

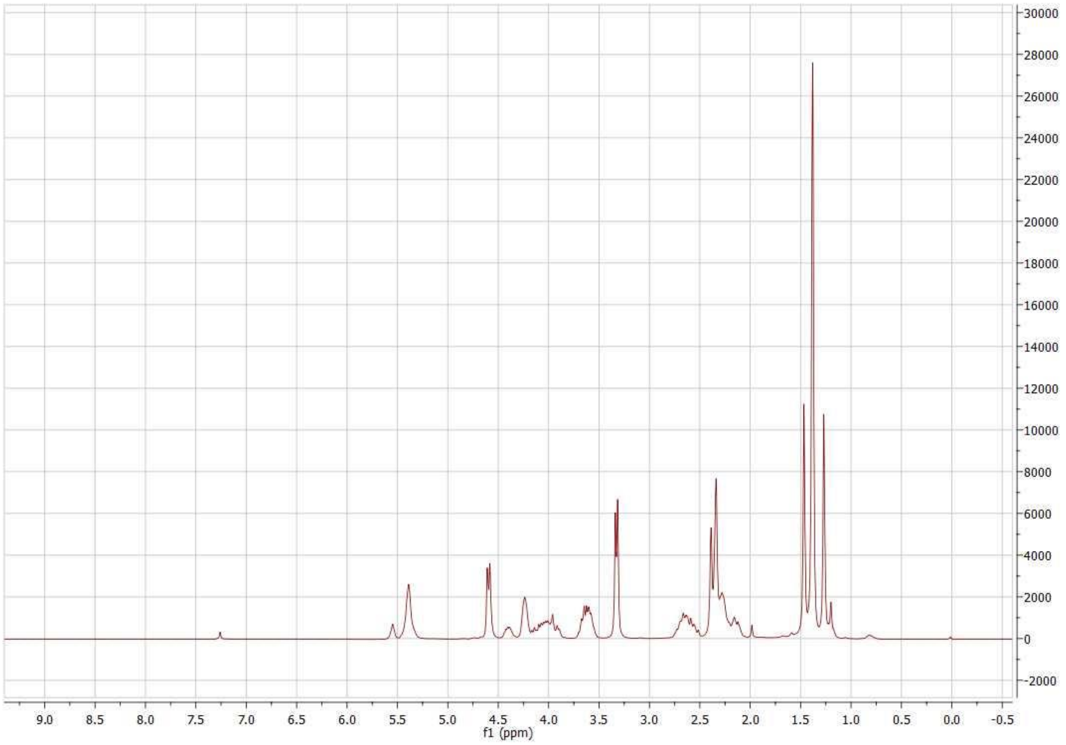

^{13}C NMR (75.5 MHz, $CDCl_3$): Compound **19**

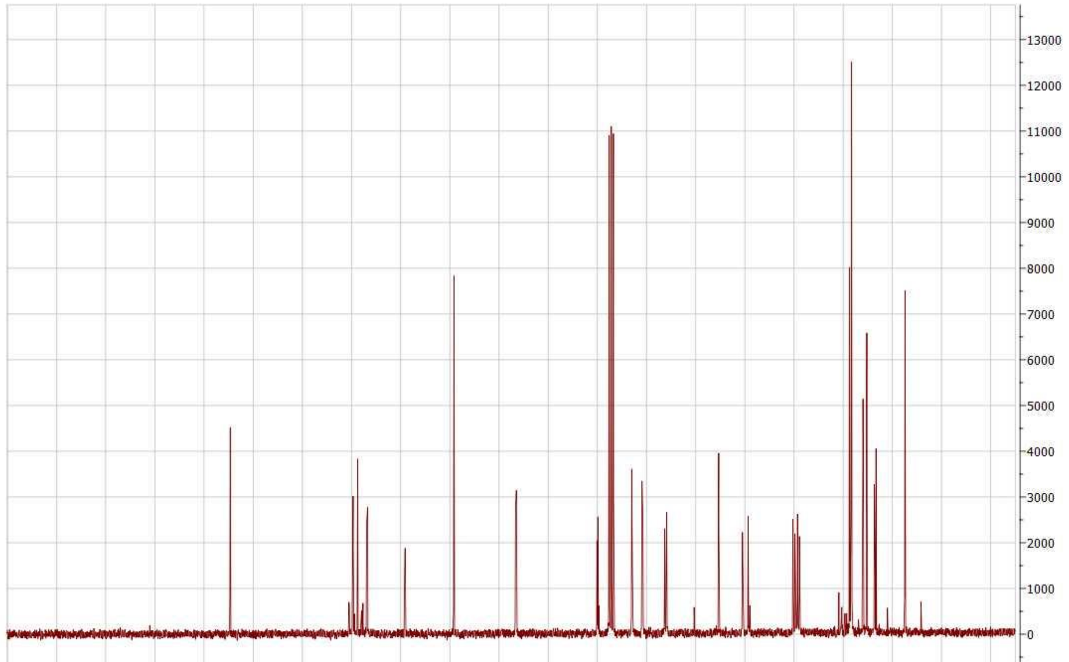

COSY ($CDCl_3$): Compound **19**

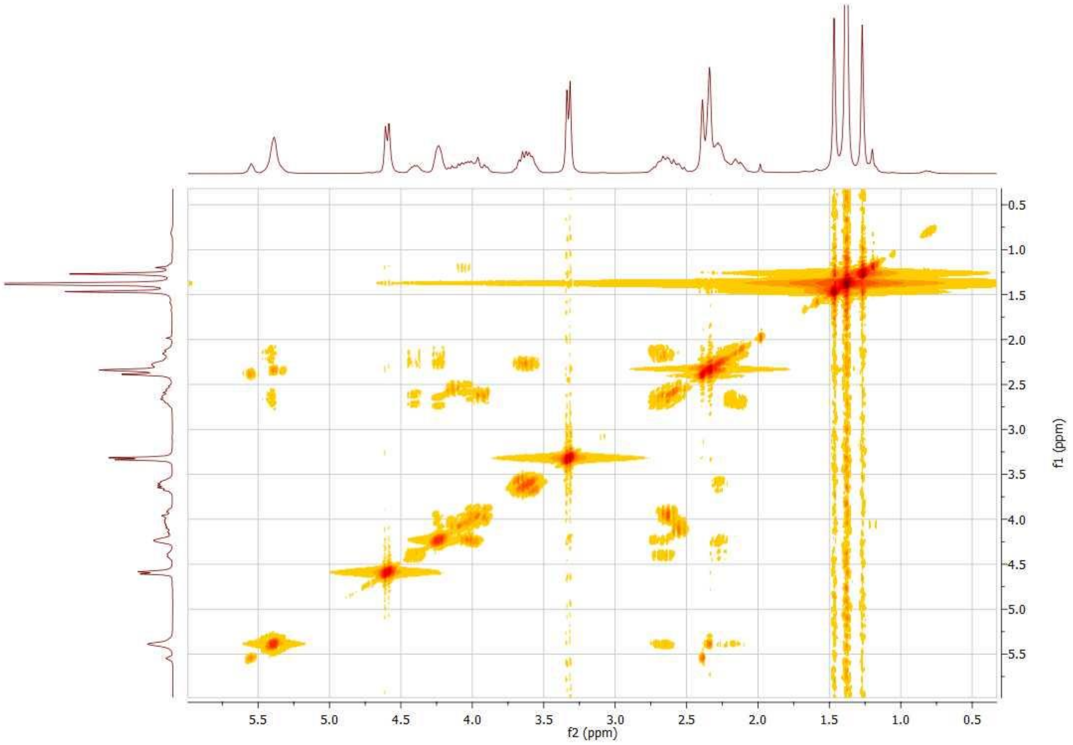

HSQC ($CDCl_3$): Compound **19**

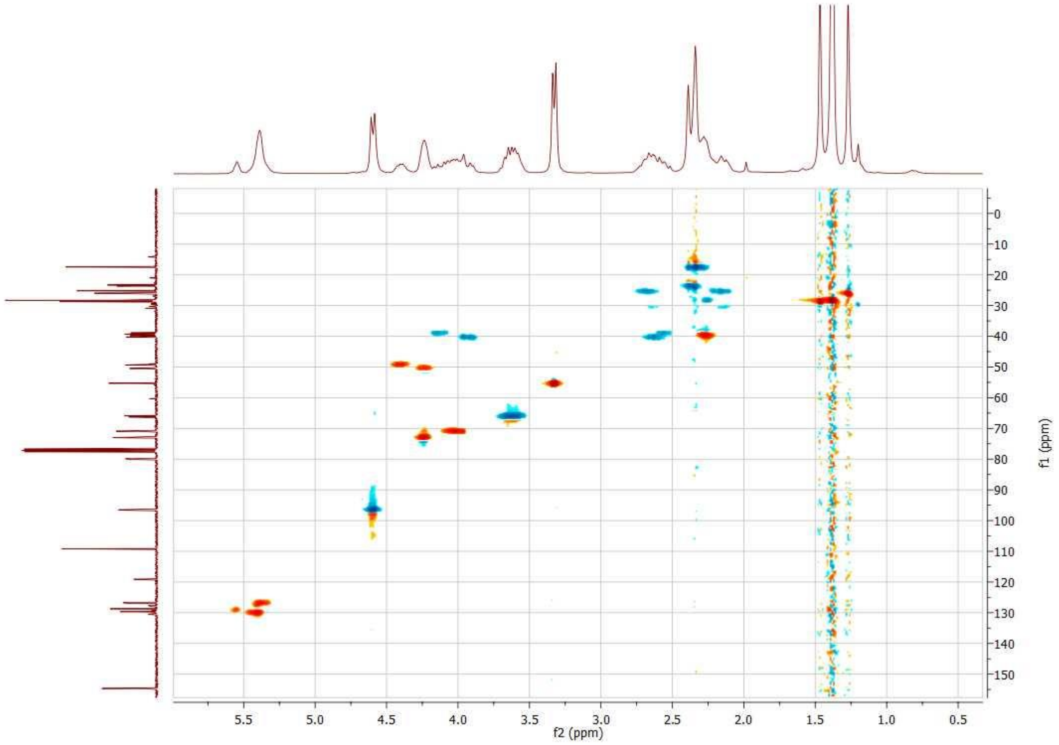

(5a*R*)-*N*-*tert*-Butyloxycarbonyl-5a-*C*-(6-amino)hexyl-3,4-*O*-isopropylidene-6-methoxymethylene-4-*epi*-isofagomine (20)

[1]**H NMR** (300 MHz, CD_3OD): Compound **20**

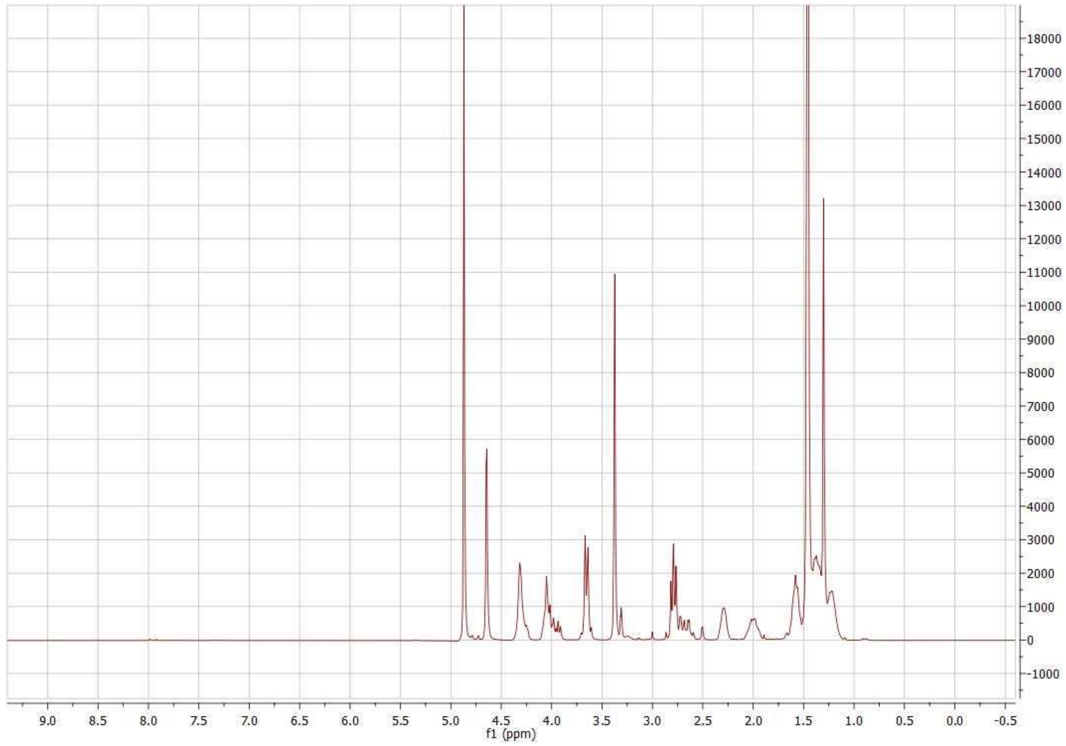

[13]**C NMR** (75.5 MHz, CD_3OD): Compound **20**

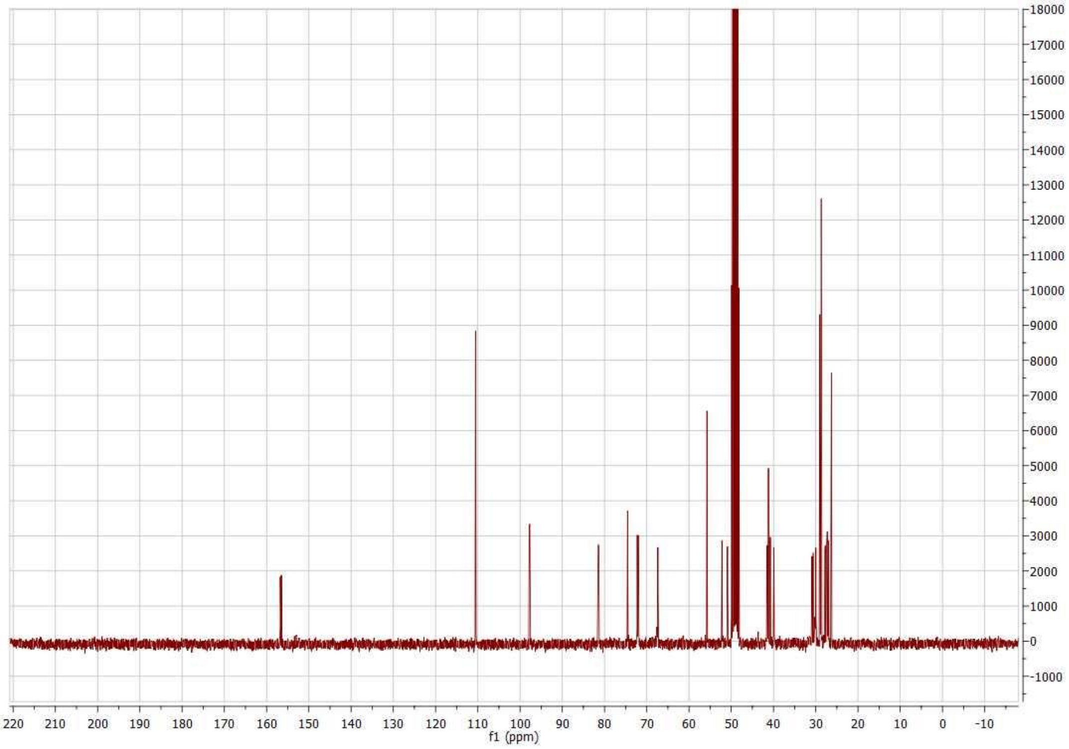

COSY (CD_3OD): Compound **20**

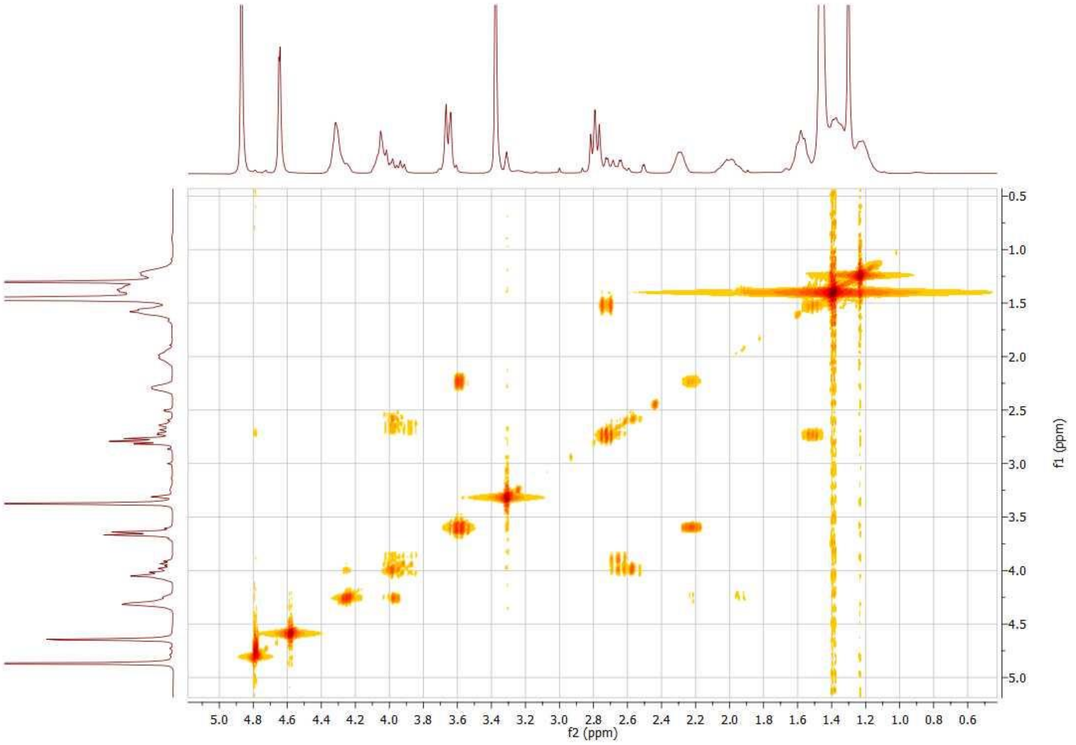

HSQC (CD_3OD): Compound **20**

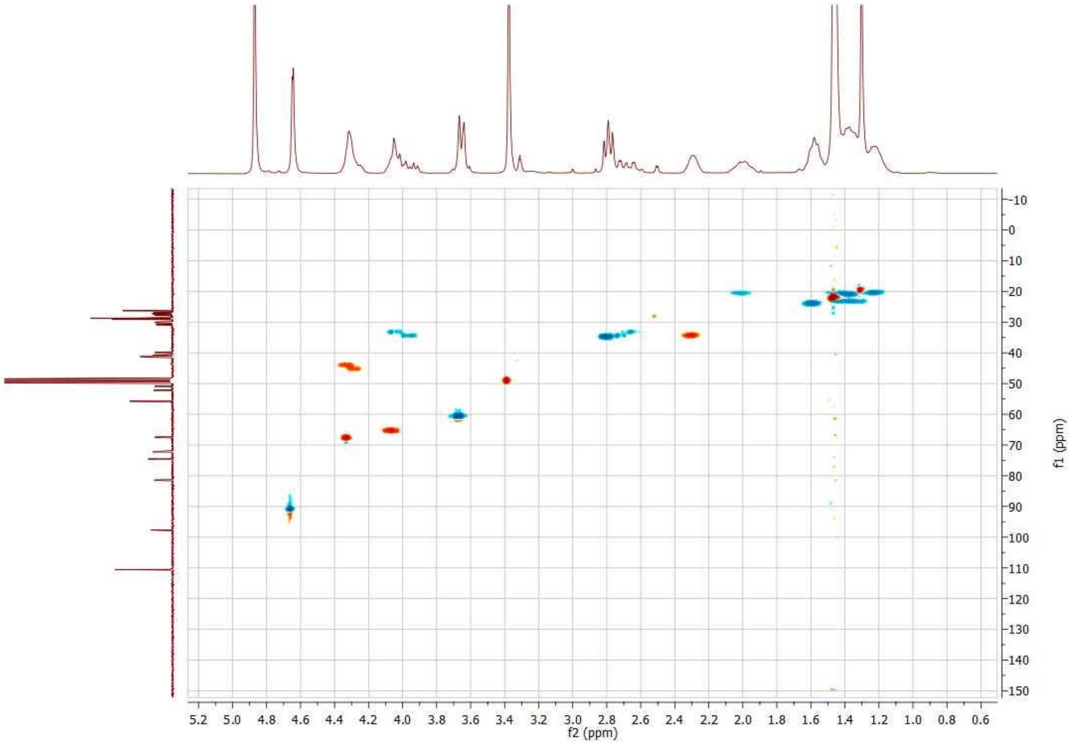

(5a*R*)-*N*-*tert*-Butyloxycarbonyl-5a-*C*-(6-dansylamino)hexyl-3,4-*O*-isopropylidene-6-*O*-methoxymethylene-4-*epi*-isofagomine (21)

[1]**H NMR** (300 MHz, $CDCl_3$): Compound **21**

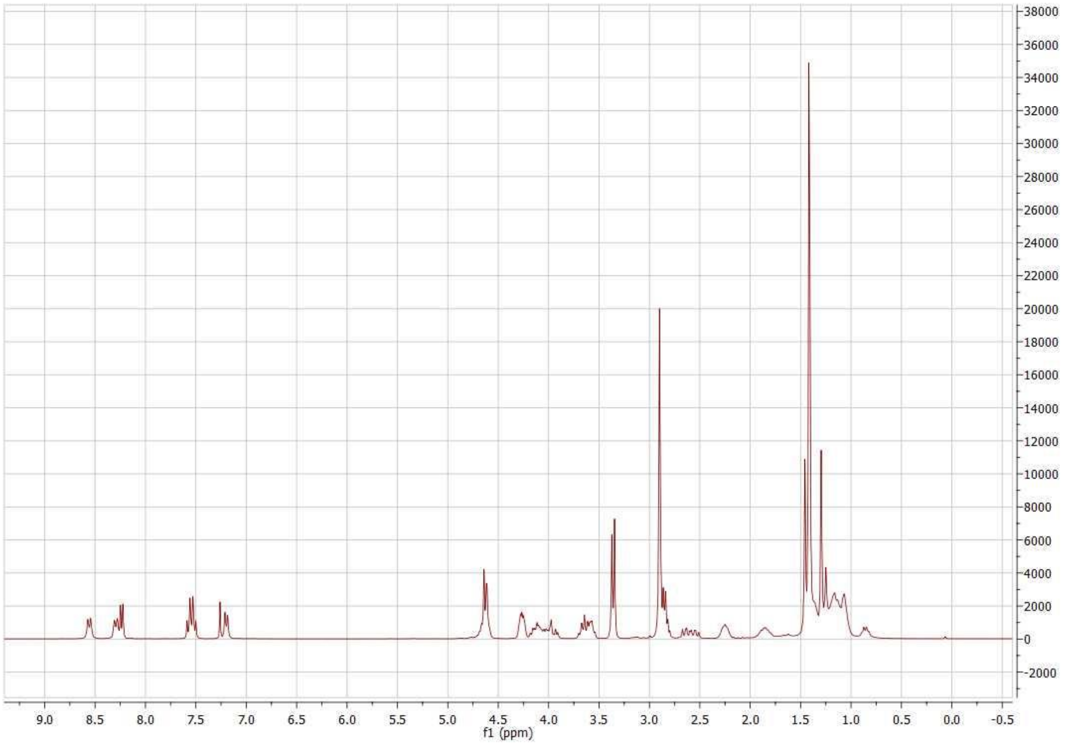

[13]**C NMR** (75.5 MHz, $CDCl_3$): Compound **21**

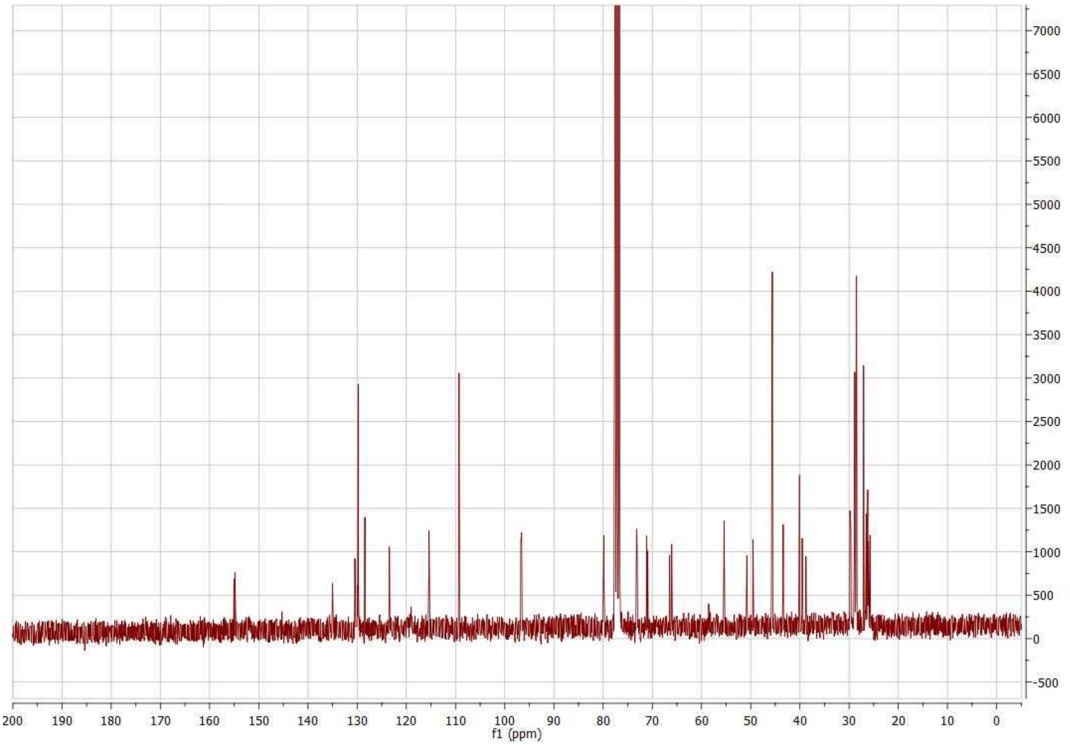

COSY (CDCl$_3$): Compound **21**

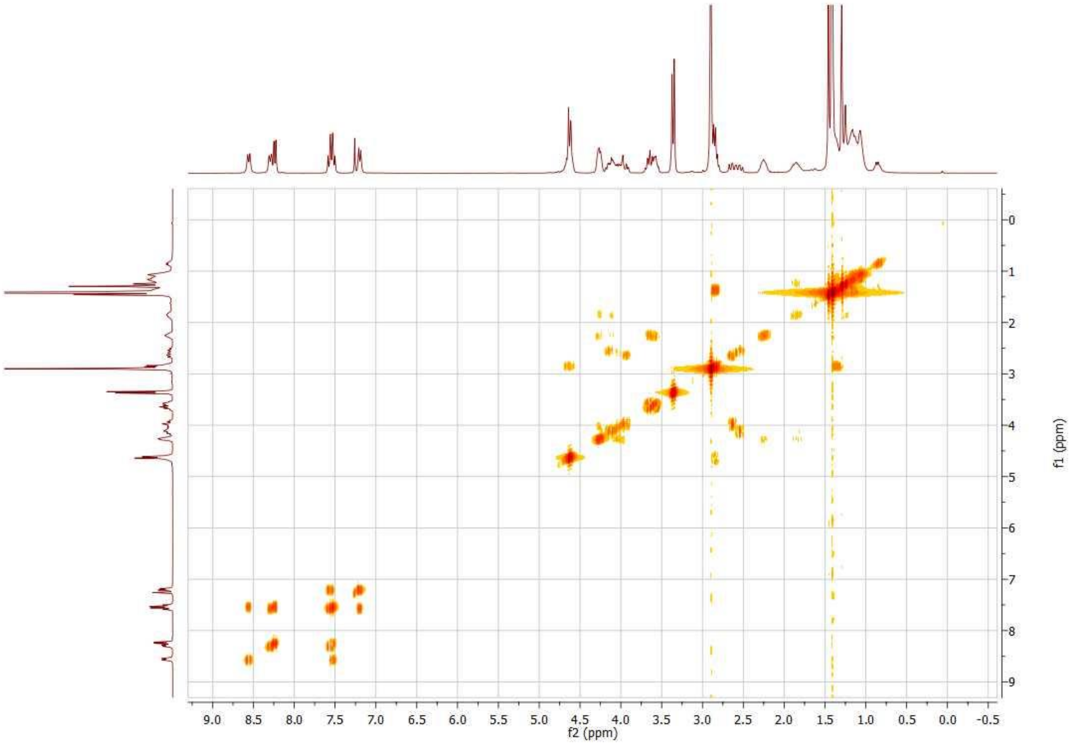

HSQC (CDCl$_3$): Compound **21**

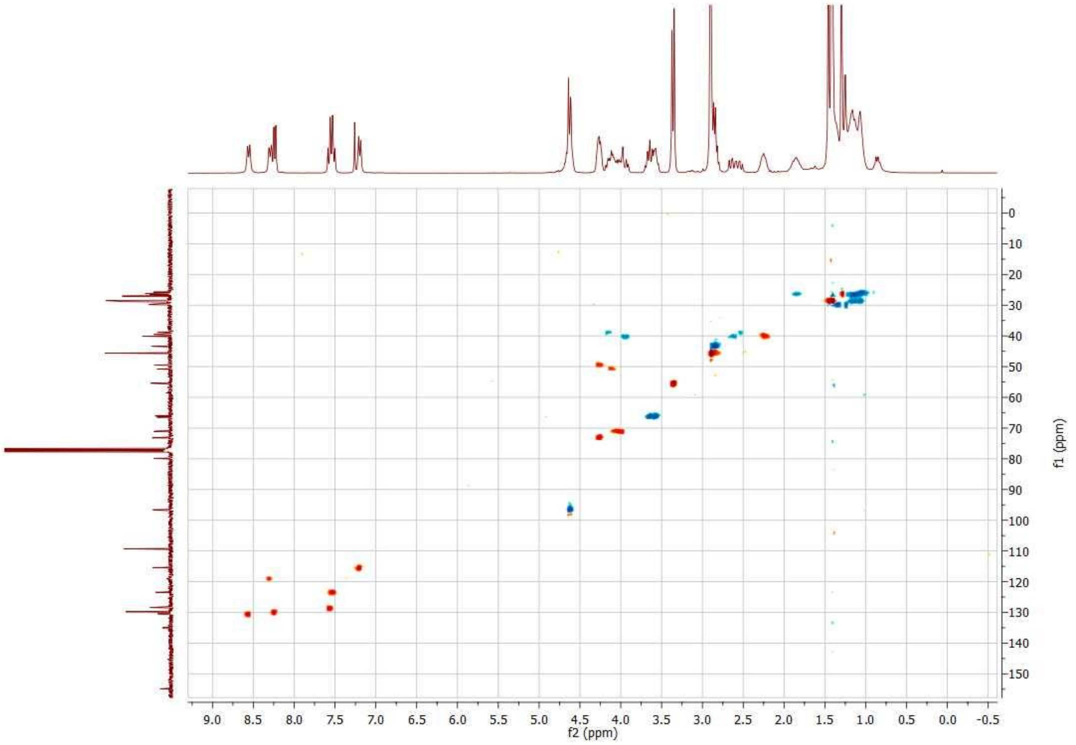

(5a*R*)-5a-*C*-(6-Dansylamino)hexyl-4-*epi*-isofagomine (22)

^{1}H NMR (300 MHz, CD_3OD): Compound **22**

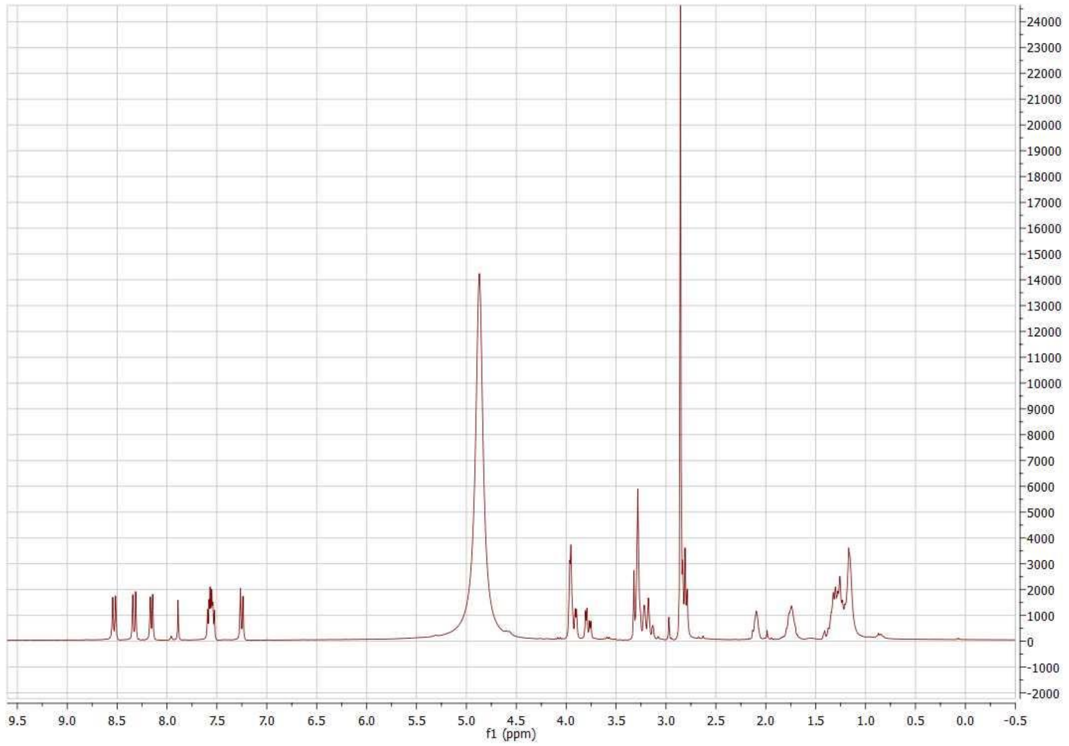

^{13}C NMR (75.5 MHz, CD_3OD): Compound **22**

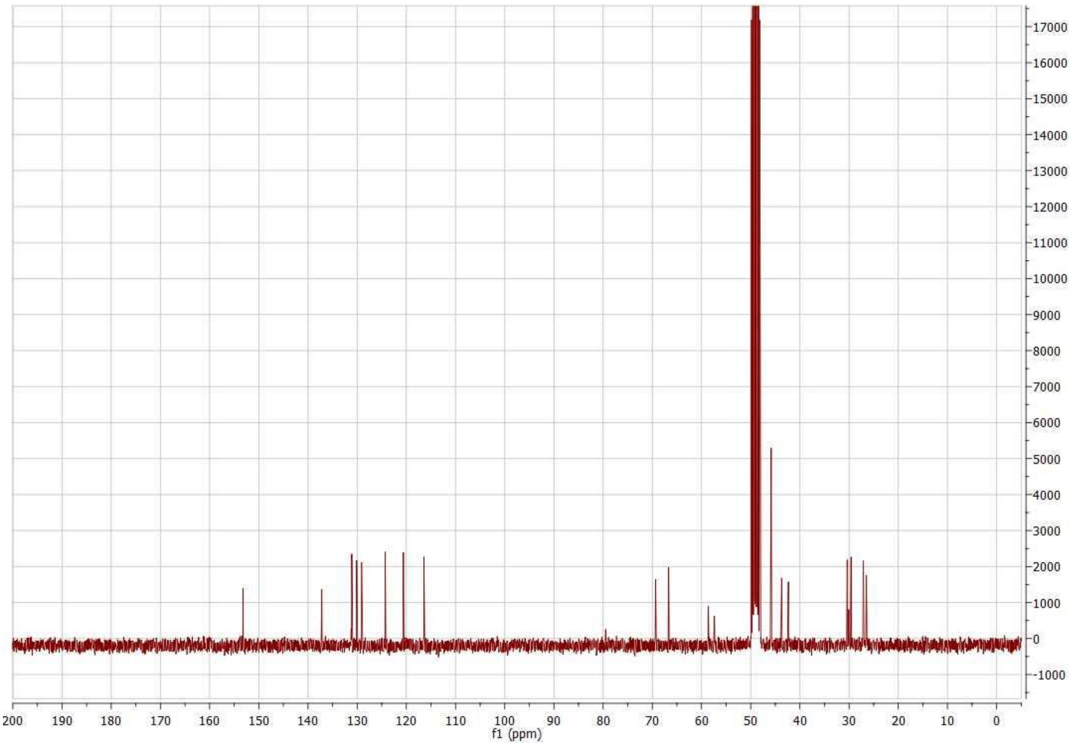

COSY (CD_3OD): Compound **22**

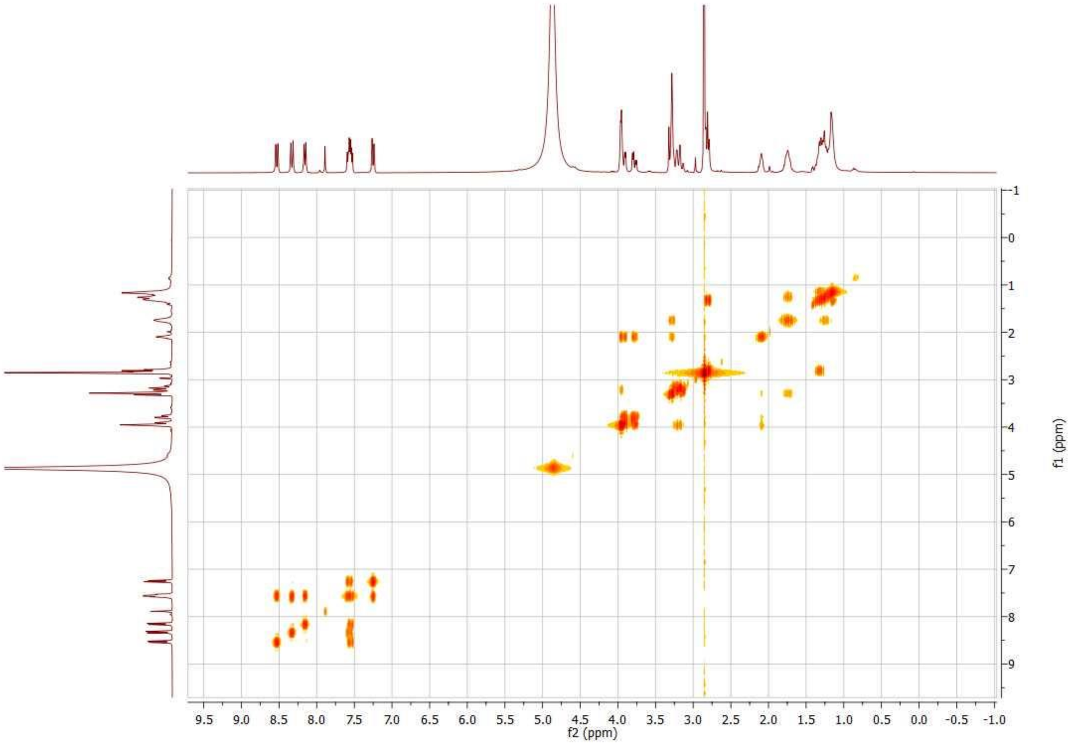

HSQC (CD_3OD): Compound **22**

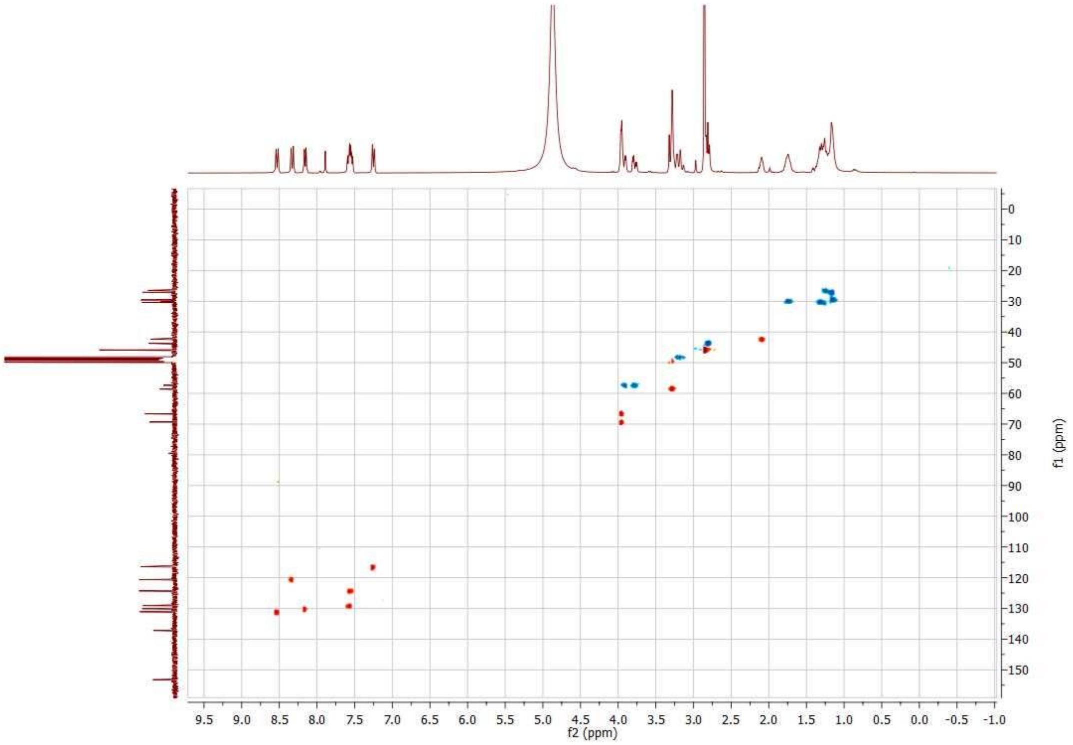

(3a*R*,3b*S*,6a*S*,7*S*,7a*R*)-1-Benzyl-7-bromo-5,5-dimethylhexahydro-1H[*1,3*]dioxolo [*4′5′:3,4*] cyclopenta[*1,2-c*]isoxazole (**25**)

^{1}H NMR (500 MHz, $CDCl_3$): Compound **25**

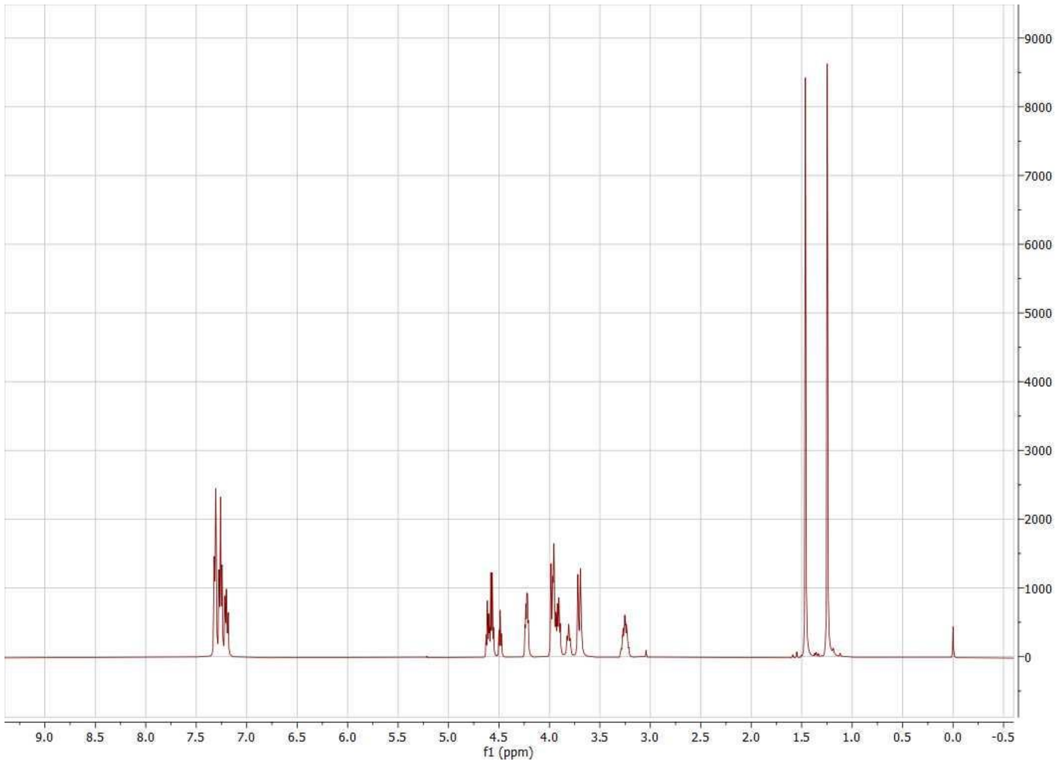

^{13}C NMR (125.9 MHz, $CDCl_3$): Compound **25**

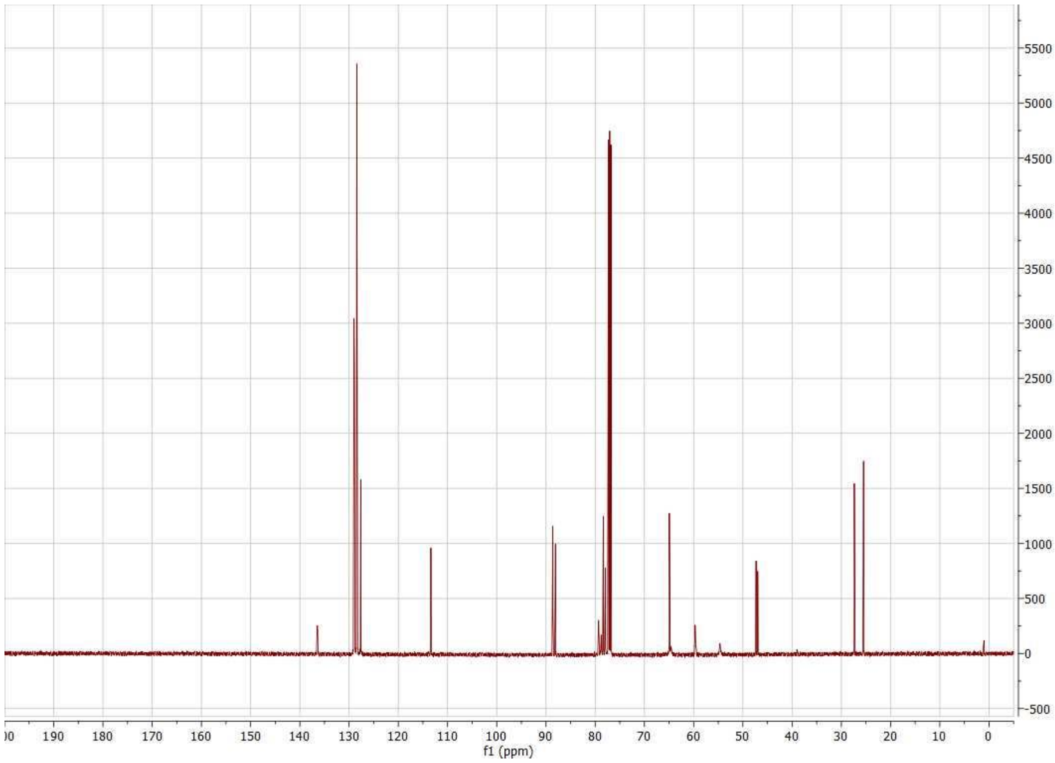

COSY ($CDCl_3$): Compound **25**

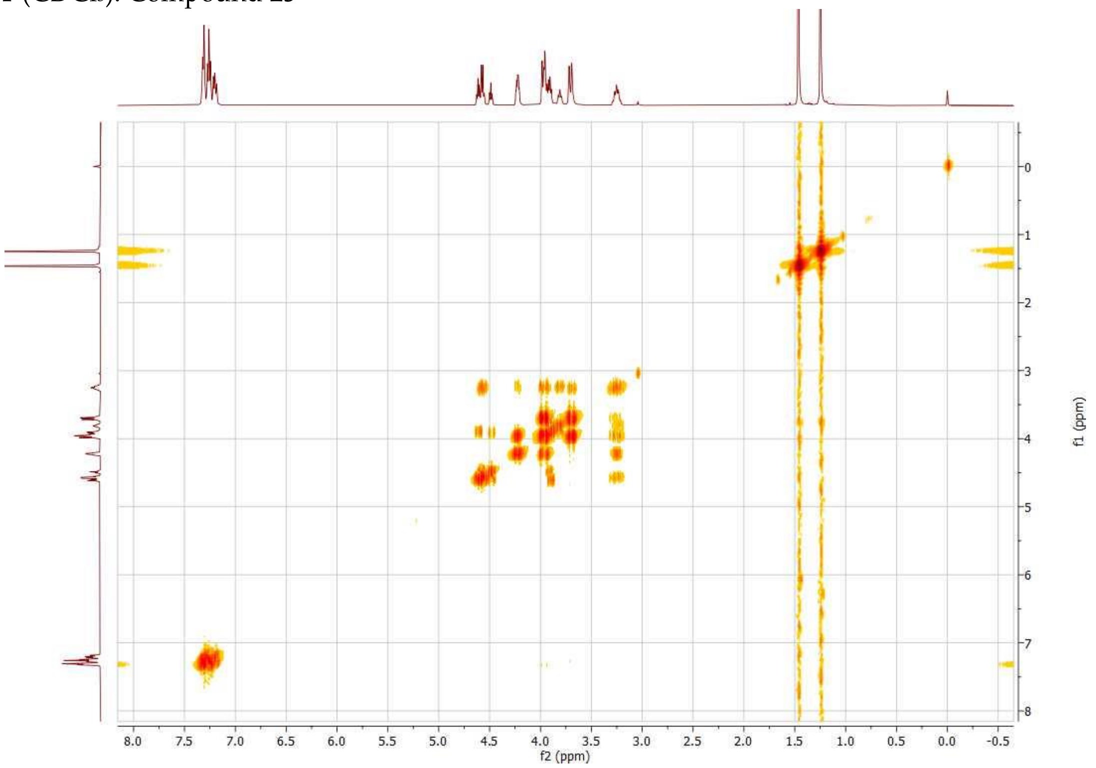

HSQC ($CDCl_3$): Compound **25**

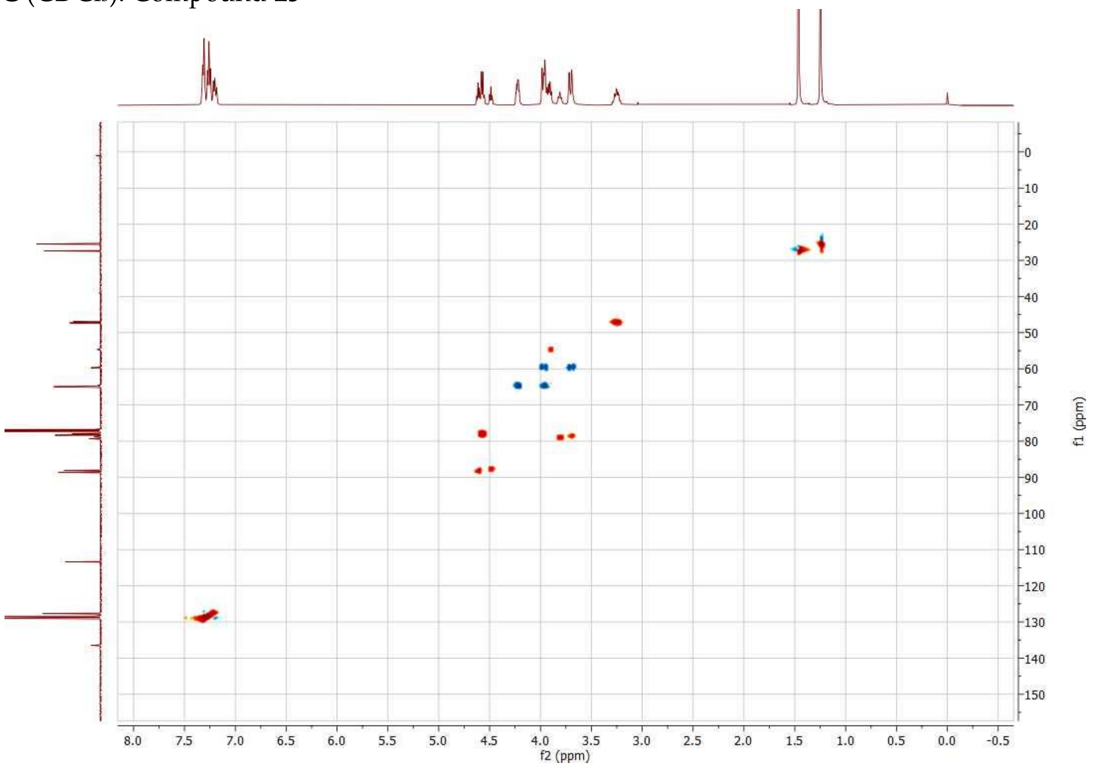

[(3a*S*,4*R*,5*S*,6a*R*)-5-Amino-2,3-dimethyltetrahydro-4H-cyclopenta[*d*][*1,3*]dioxol-4-yl]methanol (**26**)

^{1}H NMR (500 MHz, CD_3OD): Compound **26**

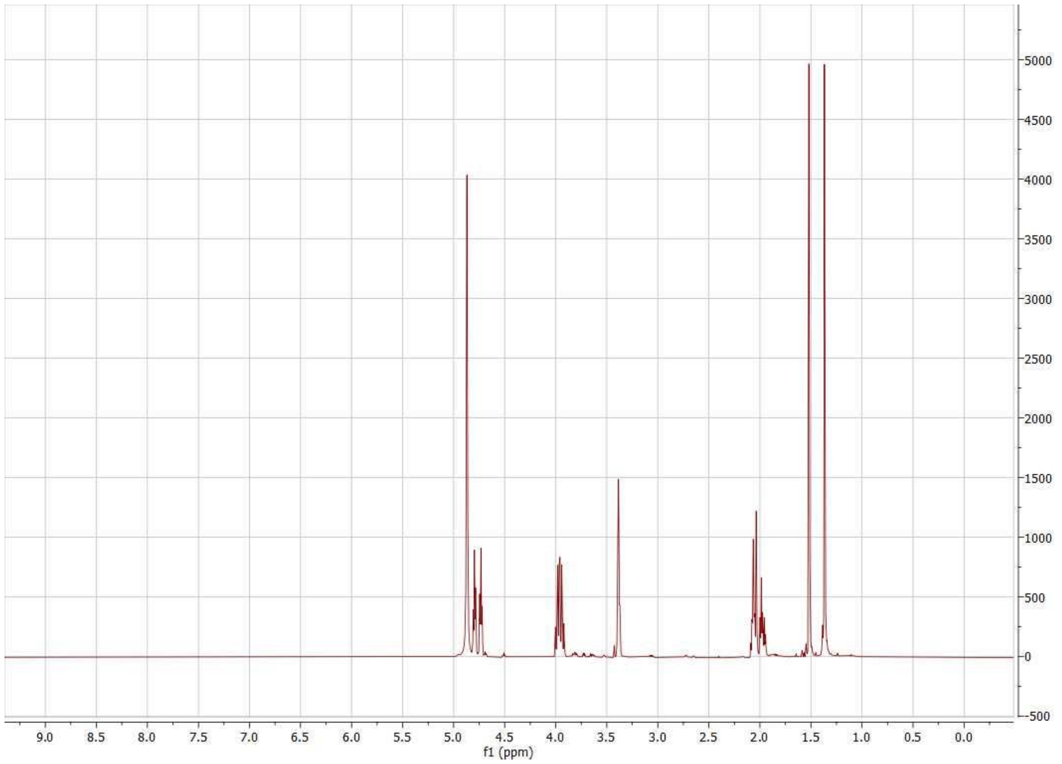

^{13}C NMR (125.9 MHz, CD_3OD): Compound **26**

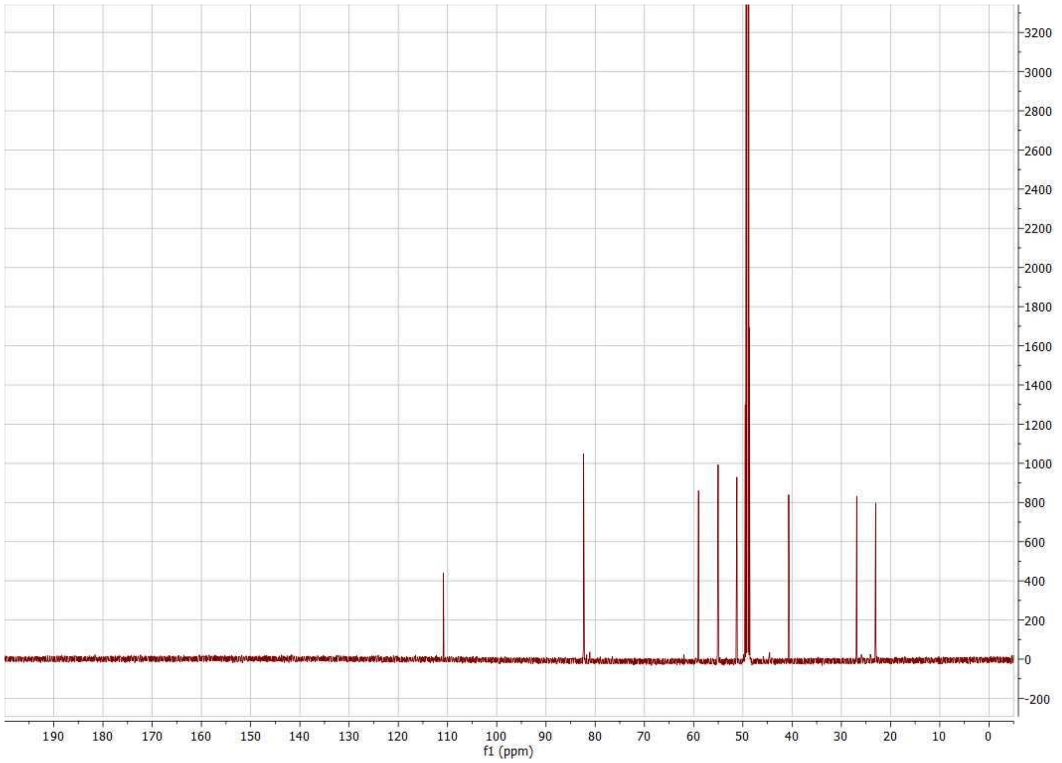

COSY (CD_3OD): Compound **26**

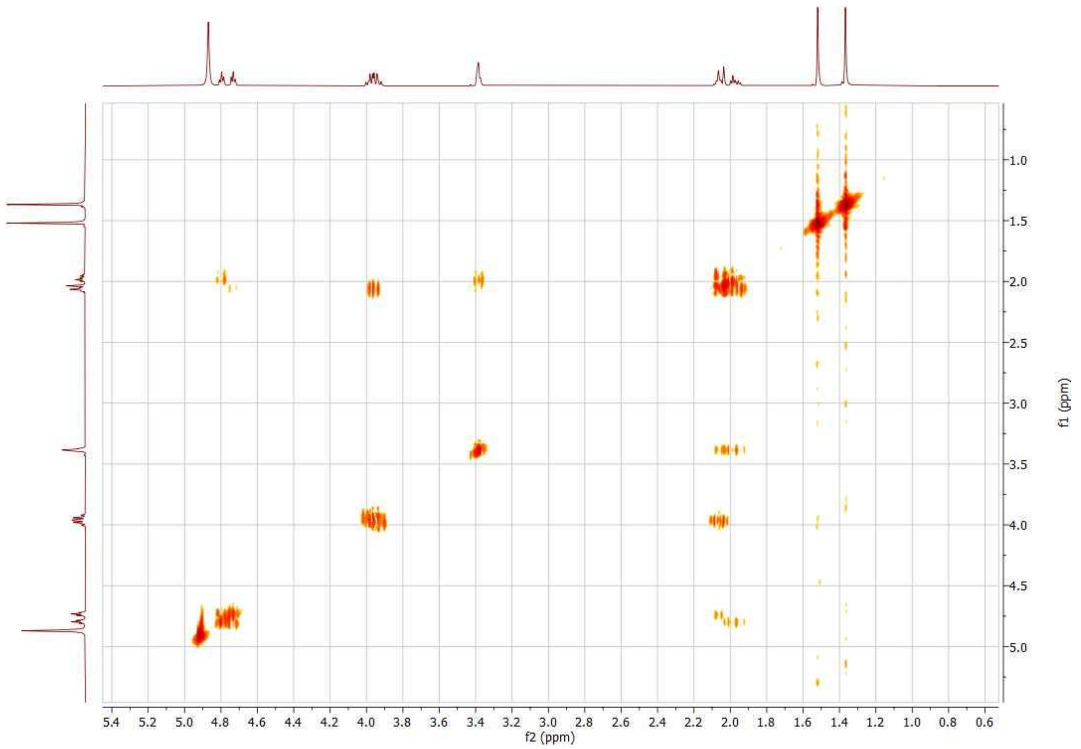

HSQC (CD_3OD): Compound **26**

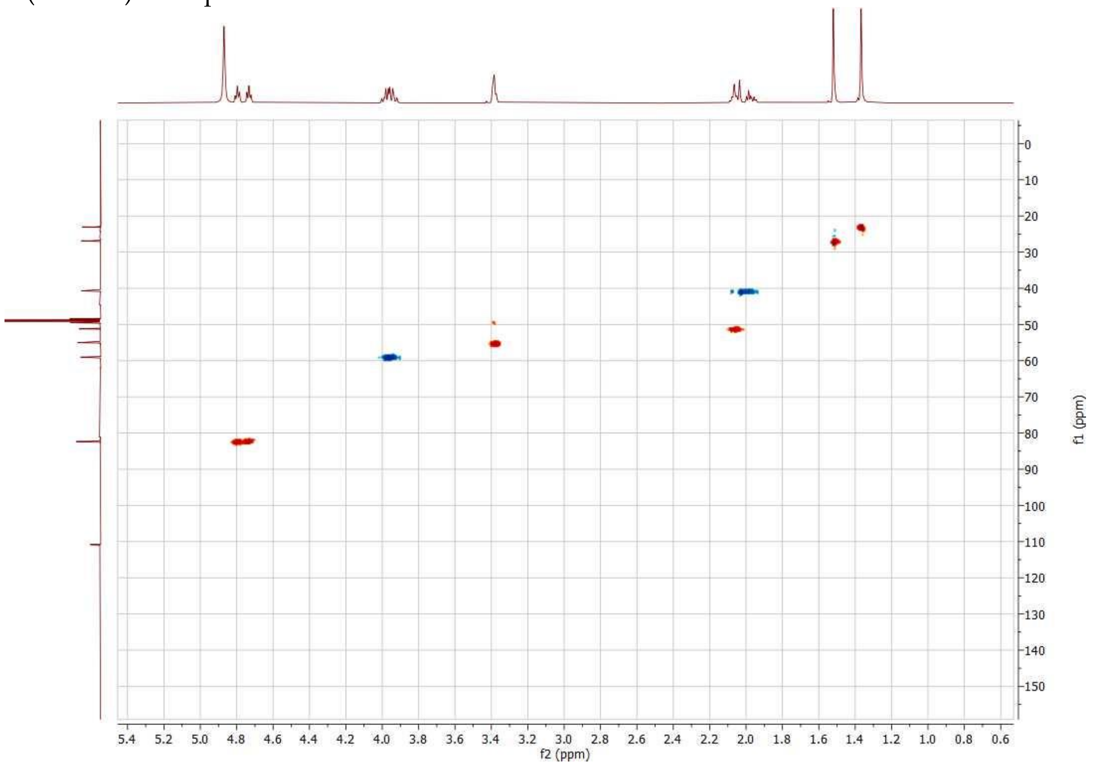

(1*R*,2*S*,3*R*,4*S*)-4-Amino-3-(hydroxymethyl)cyclopentane-1,2-diol (27)

[1]**H NMR** (300 MHz, D_2O): Compound **27**

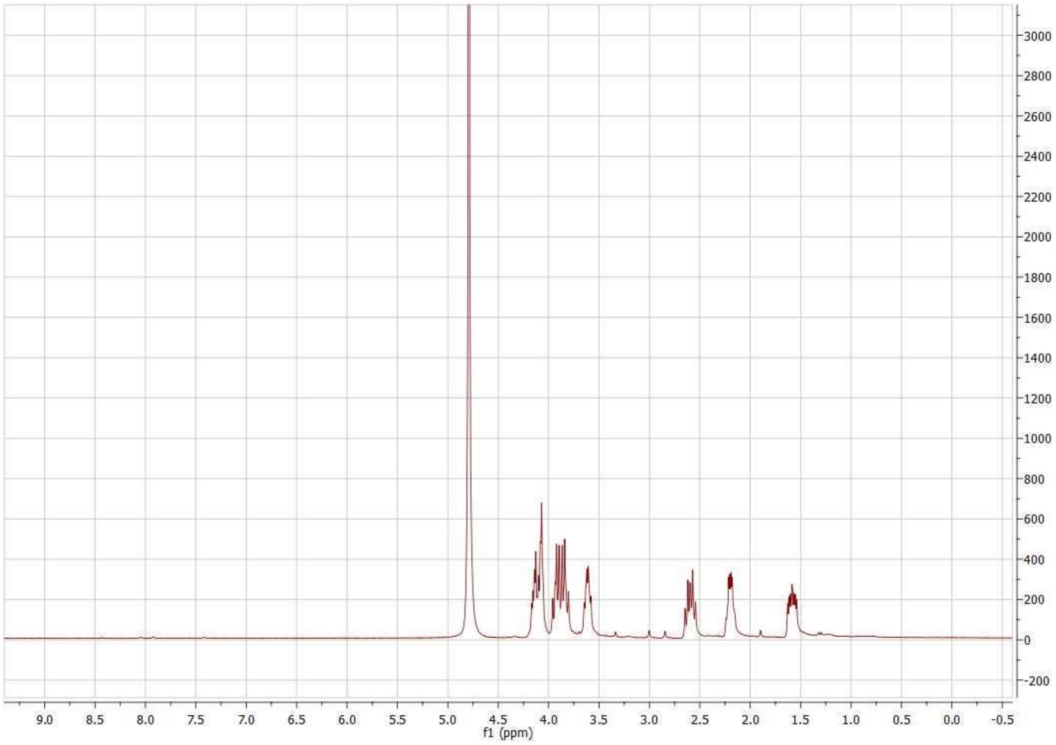

[13]**C NMR** (75.5 MHz, D_2O): Compound **27**

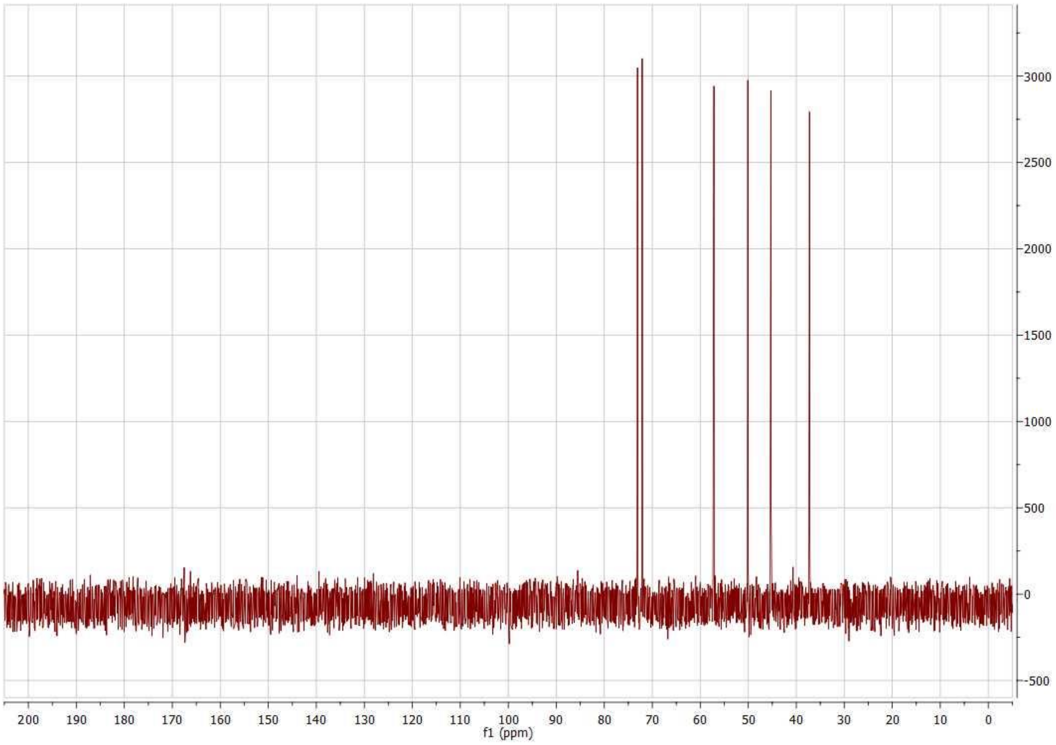

COSY (D_2O): Compound **27**

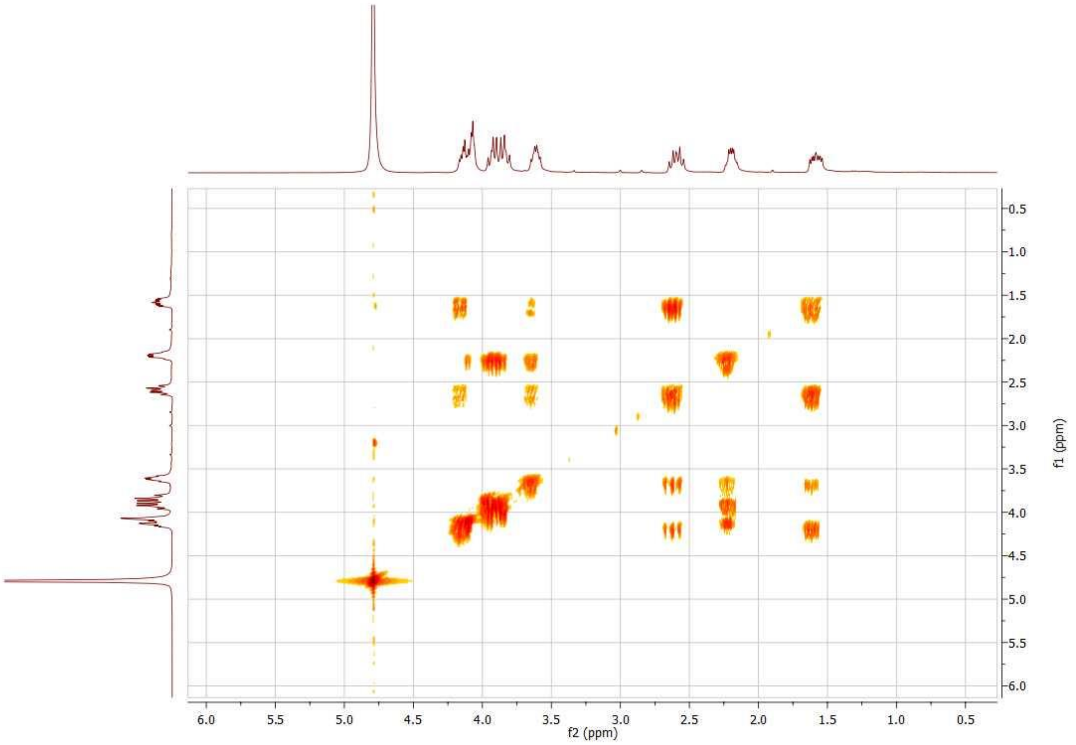

HSQC (D_2O): Compound **27**

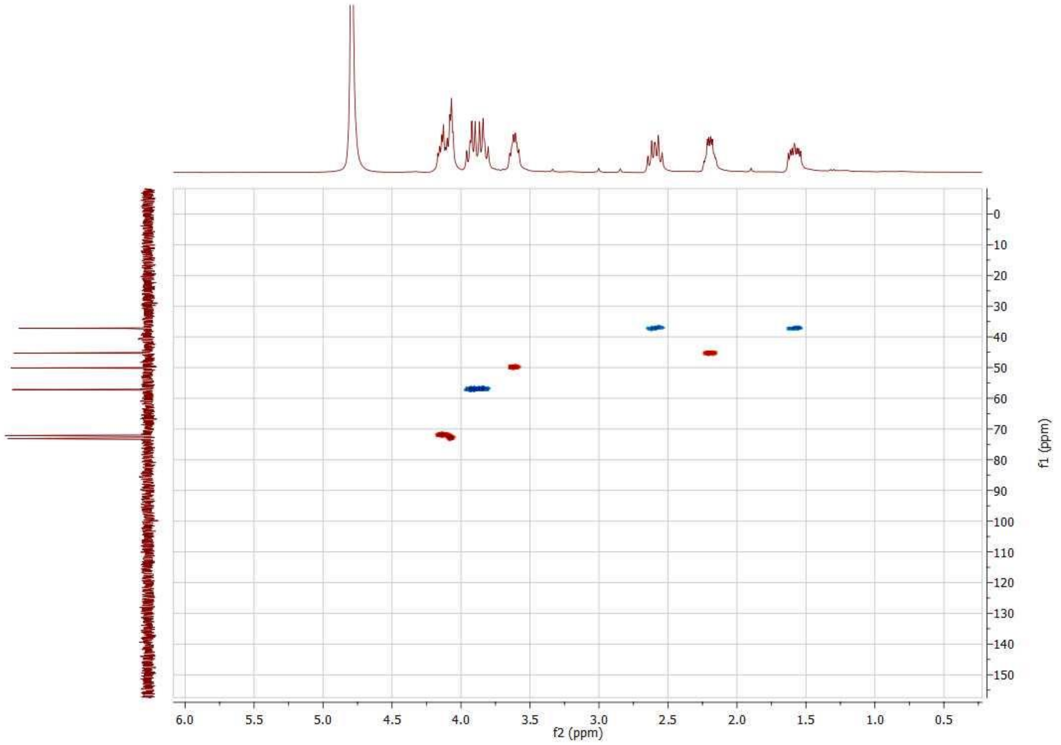

6-[(3a*S*,4*R*,5*S*,6a*R*)-4-Hydroxymethyl-2,2-dimethyltetrahydro-4H-cyclopenta[*d*][*1,3*]dioxol-5-yl)amino]hexanoic nitrile (28)

^{1}**H NMR** (300 MHz, $CDCl_3$): Compound **28**

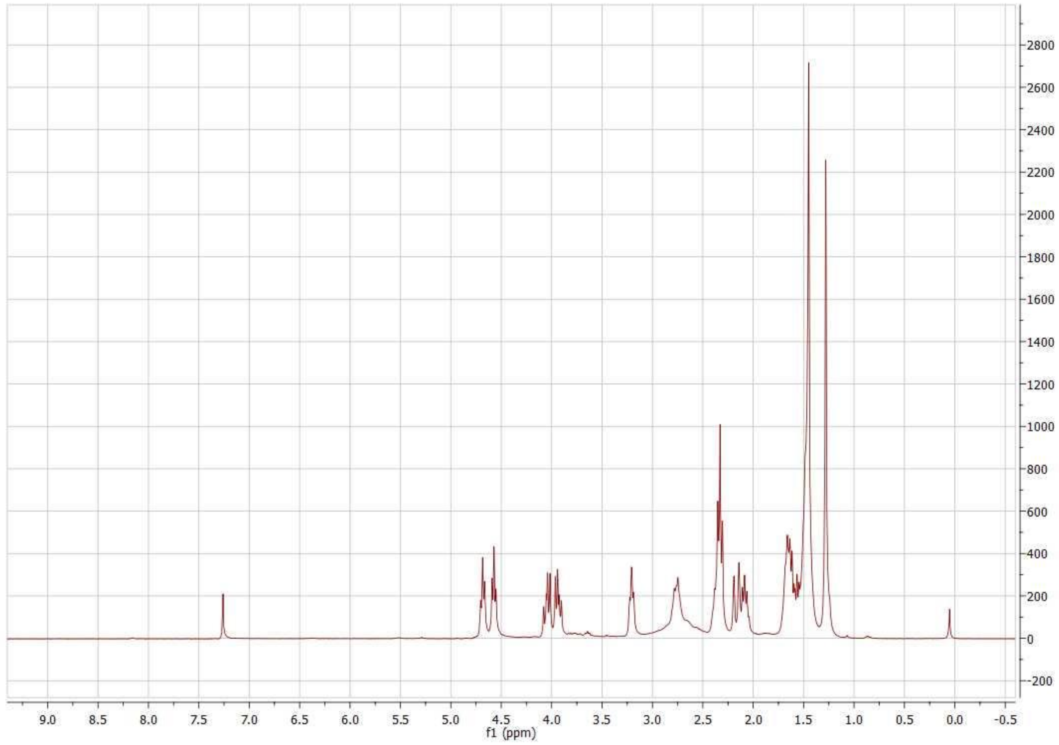

^{13}**C NMR** (75.5 MHz, $CDCl_3$): Compound **28**

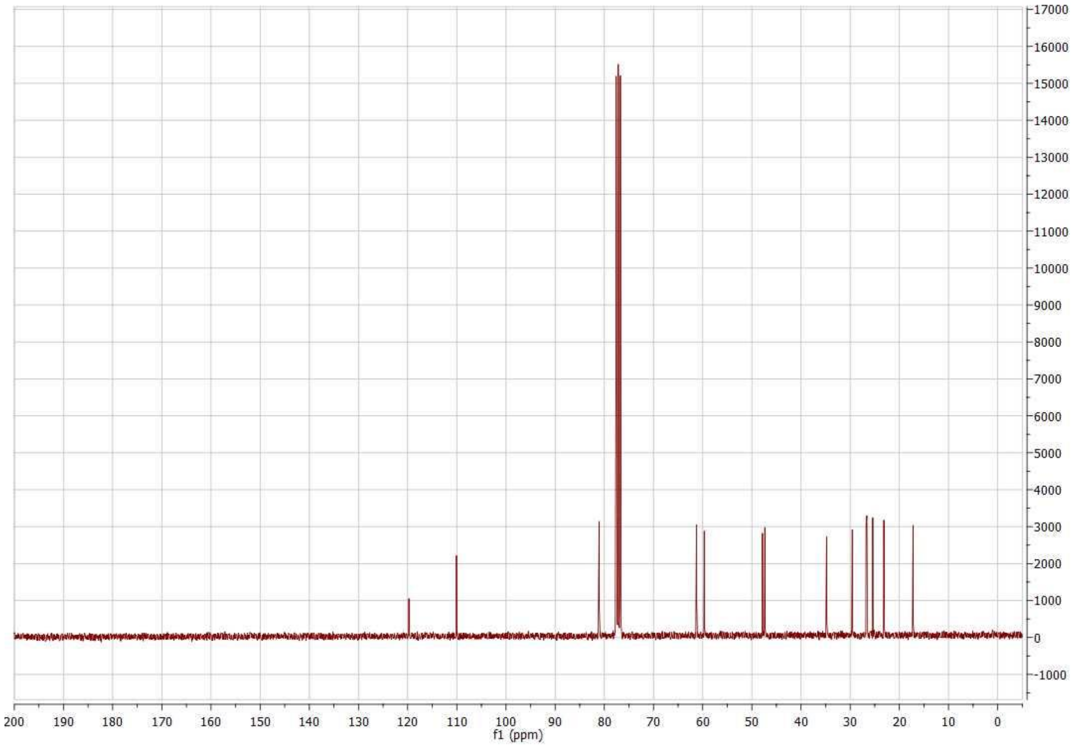

COSY (CDCl3): Compound **28**

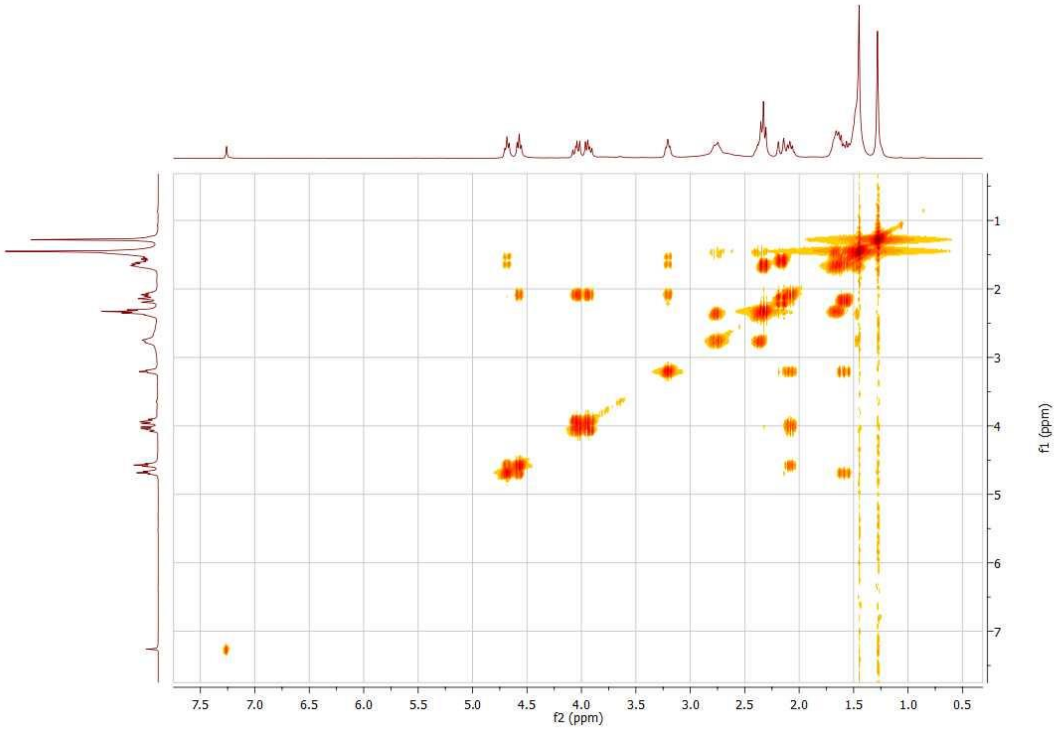

HSQC (CDCl3): Compound **28**

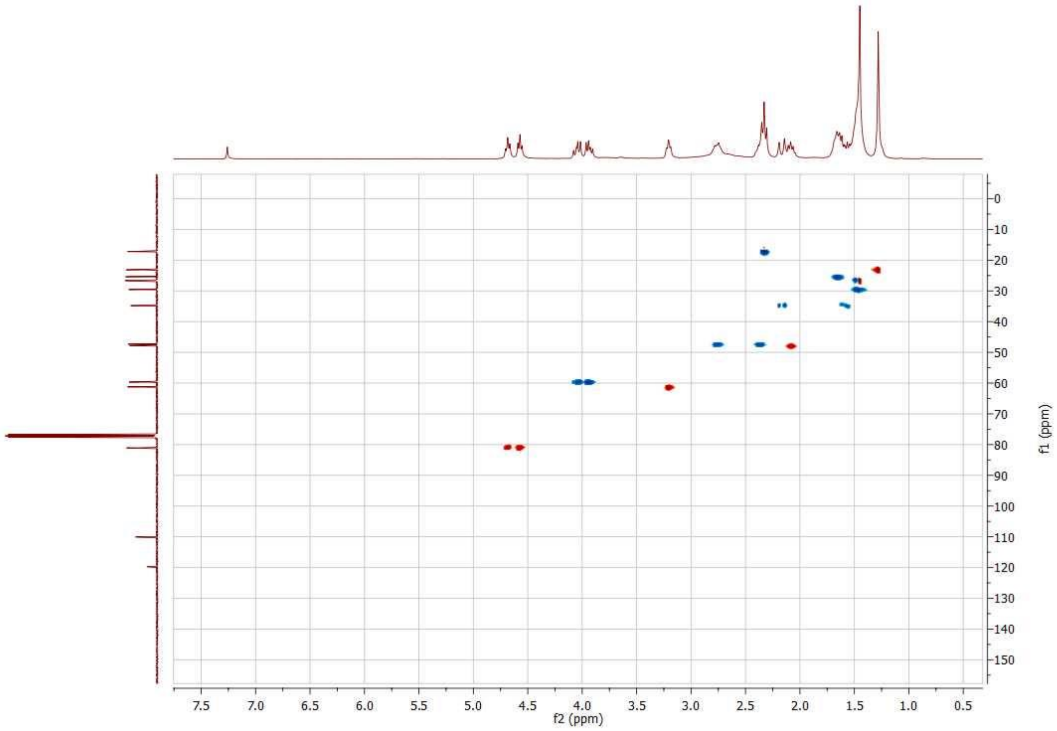

(3a*S*,4*R*,5*S*,6a*R*)-5-(6-aminohexyl)amino-2,2-dimethyltetrahydro-4H-cyclopenta[*d*][*1,3*]dioxol-4-yl]methanol (29)

^{1}H NMR (300 MHz, $CDCl_3$): Compound **29**

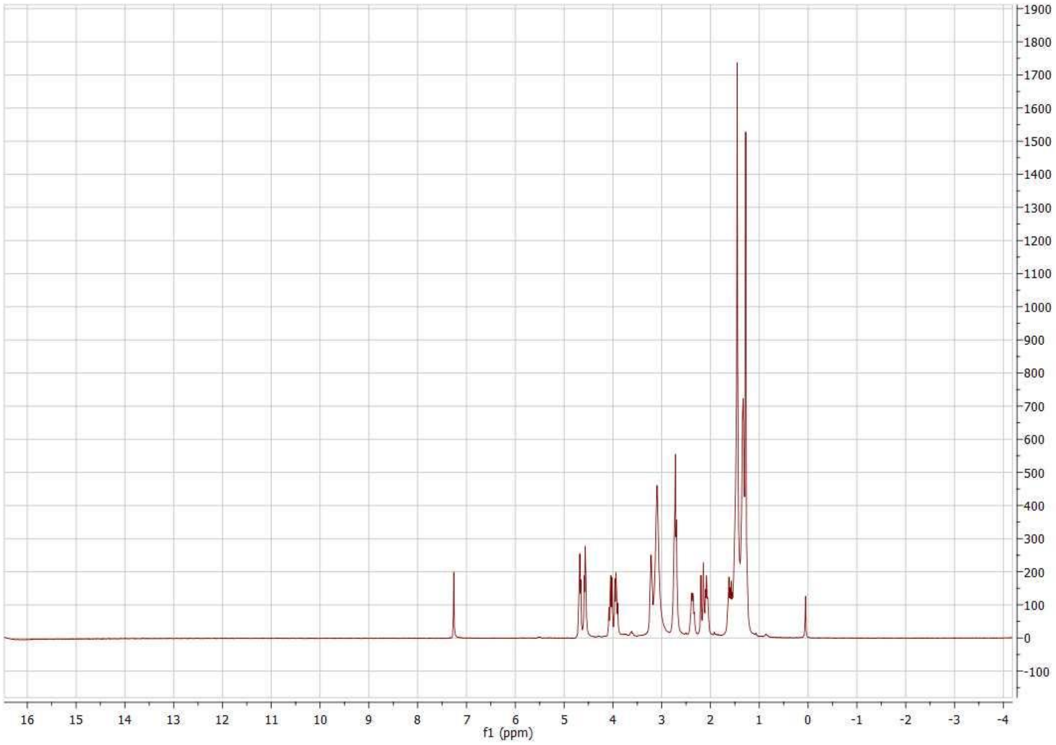

^{13}C NMR (75.5 MHz, $CDCl_3$): Compound **29**

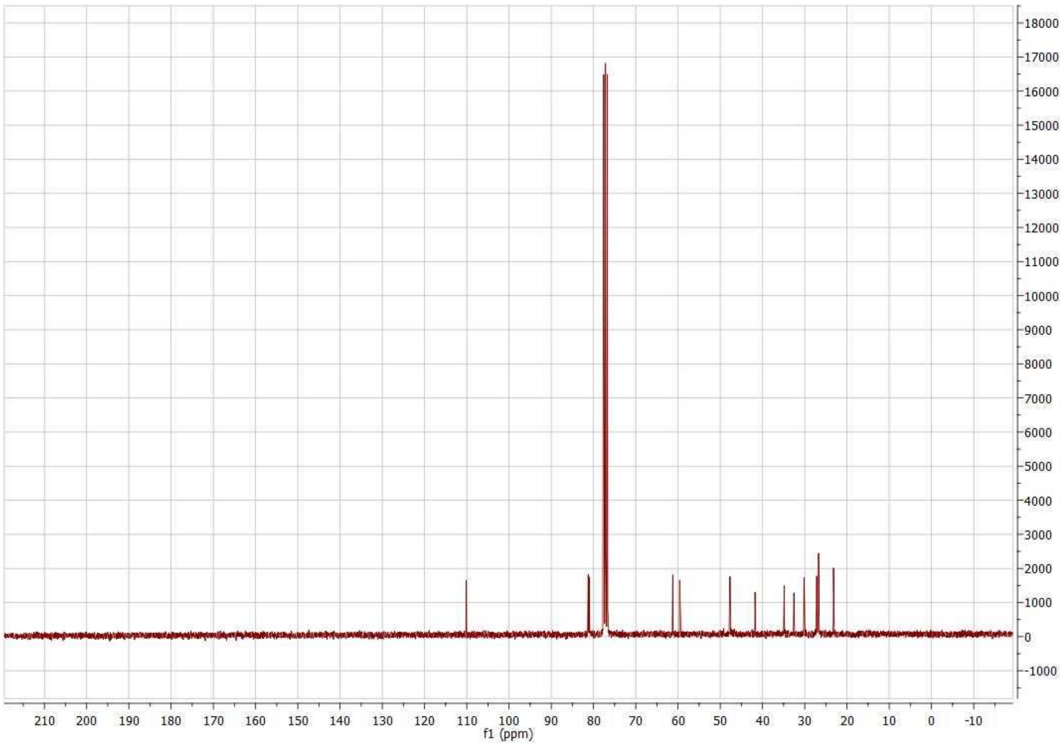

COSY ($CDCl_3$): Compound **29**

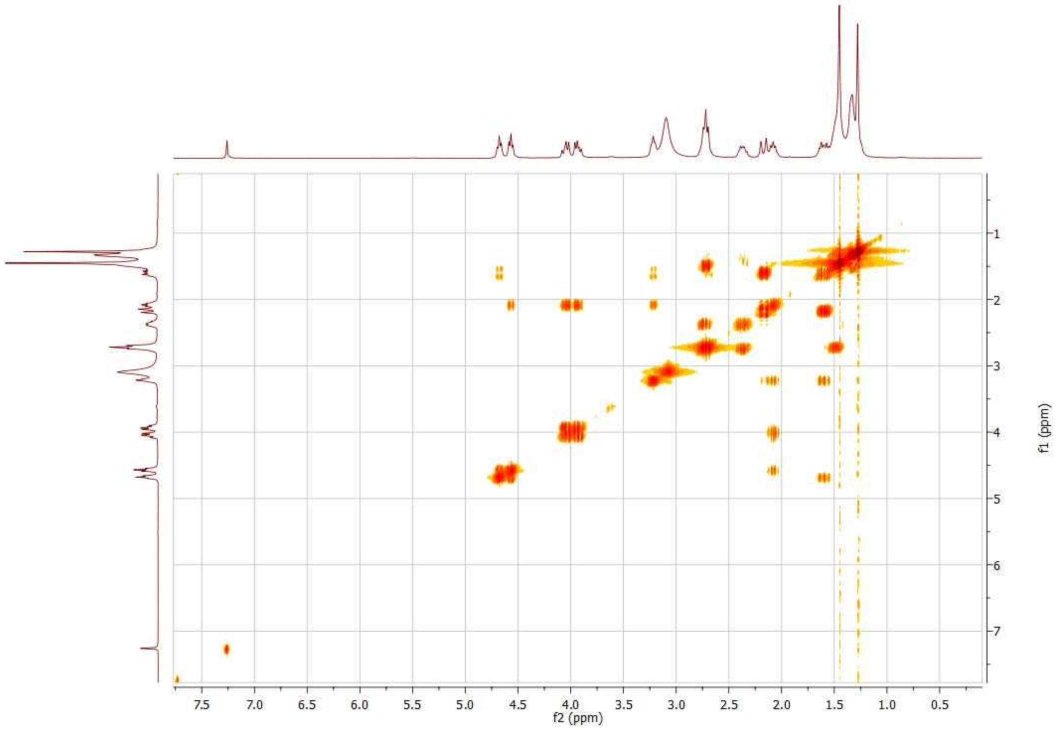

HSQC ($CDCl_3$): Compound **29**

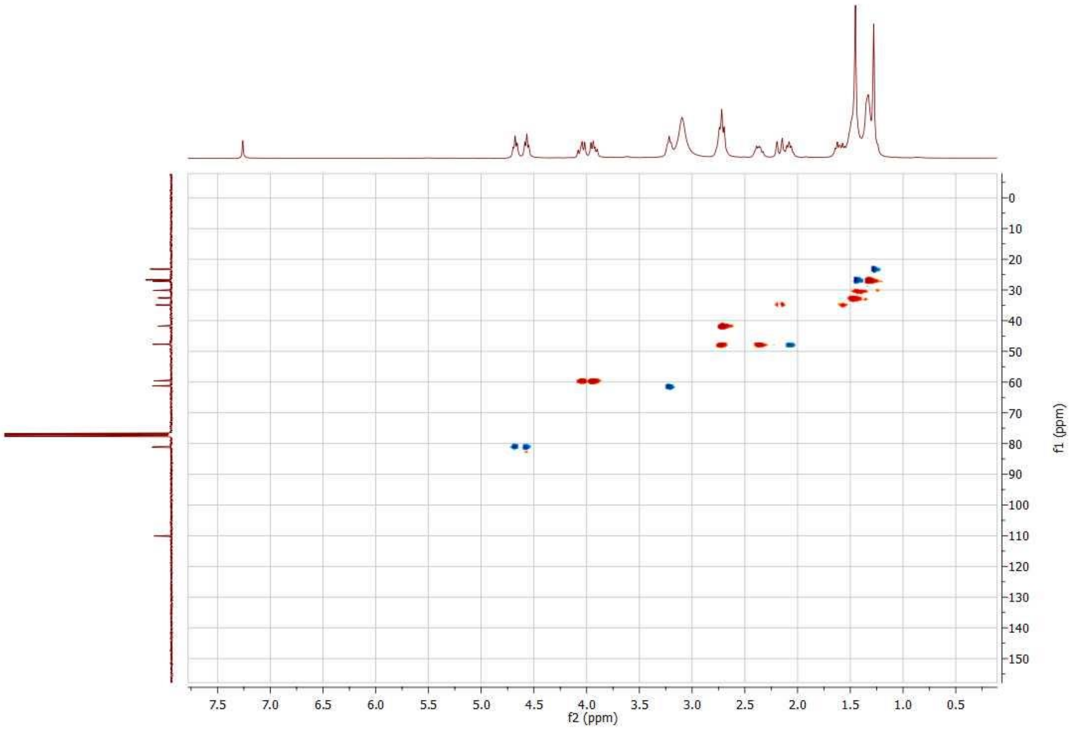

(3a*S*,4*R*,5*S*,6a*R*)-2,2-Dimethyl-5-[6-(dansylamino)hexyl]aminotetrahydro-4H-cyclopenta[*d*][*1,3*]dioxol-4-yl)methanol (**30**)

[1]**H NMR** (300 MHz, $CDCl_3$): Compound **30**

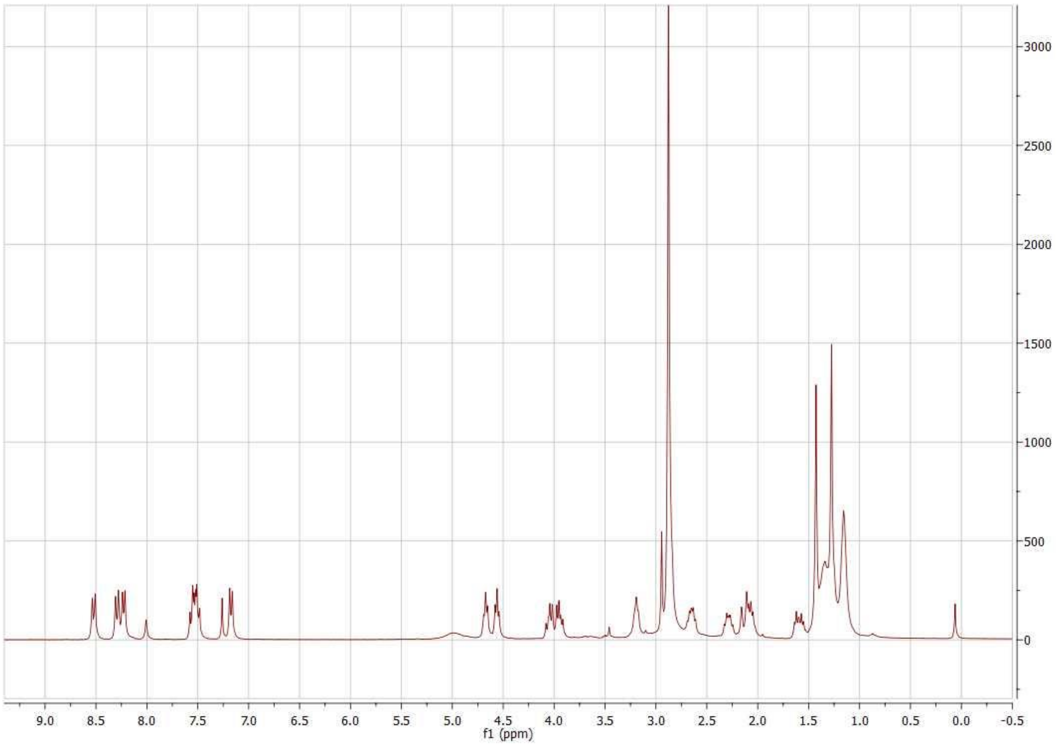

[13]**C NMR** (75.5 MHz, $CDCl_3$): Compound **30**

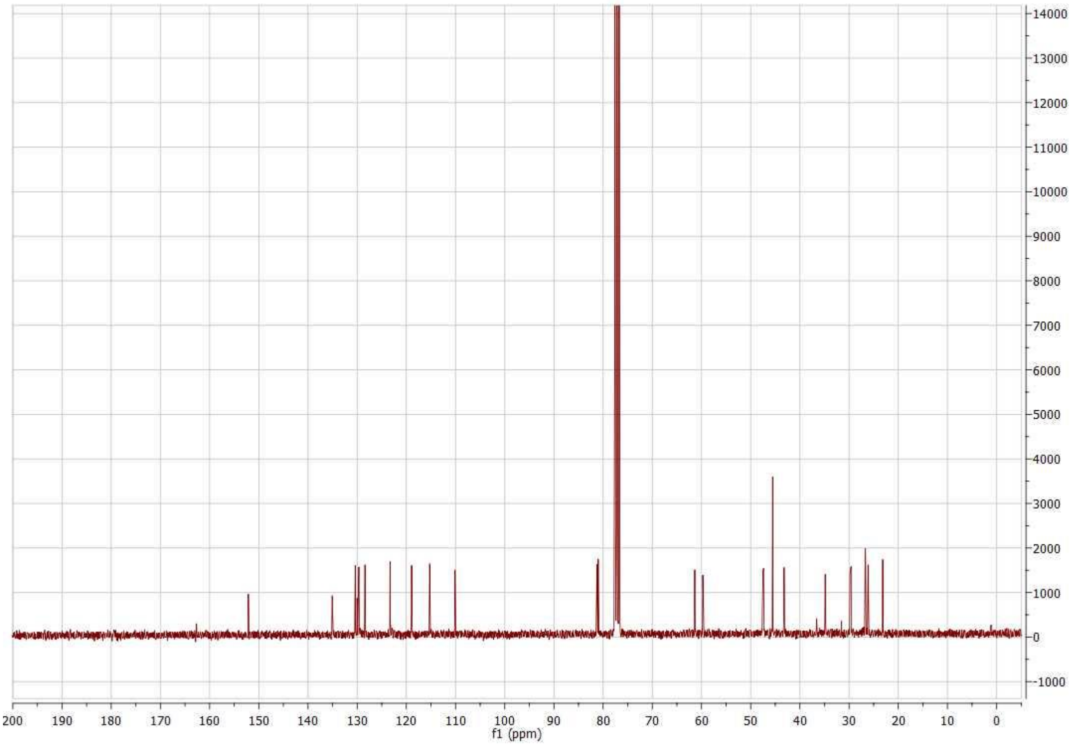

COSY ($CDCl_3$): Compound **30**

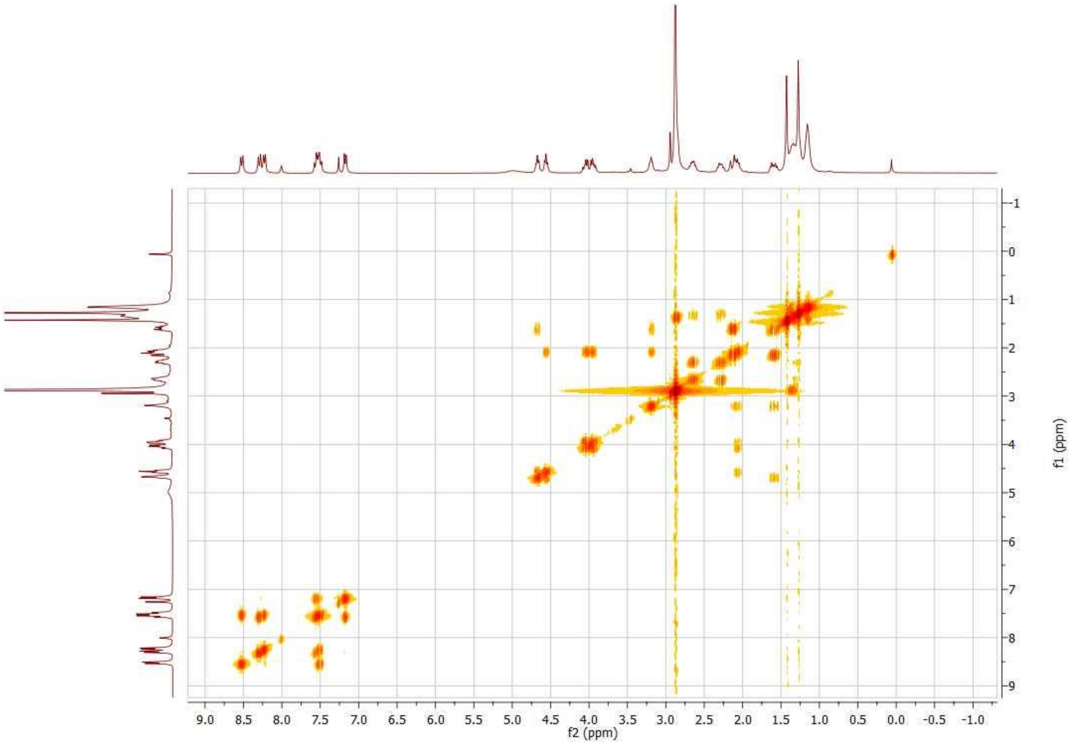

HSQC ($CDCl_3$): Compound **30**

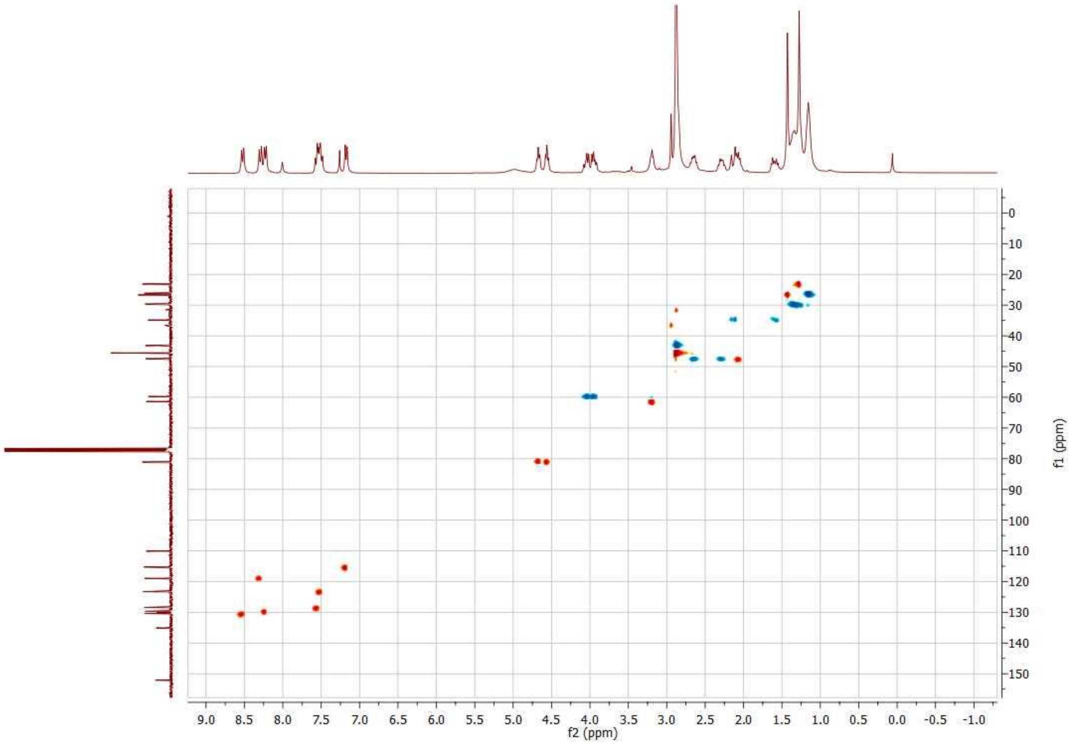

(1*R*,2*S*,3*R*,4*S*)-3-Hydroxymethyl-4-(6′-dansylaminohexylamino)cyclopentane-1,2-diol (31)

[1]**H NMR** (300 MHz, CD_3OD): Compound **31**

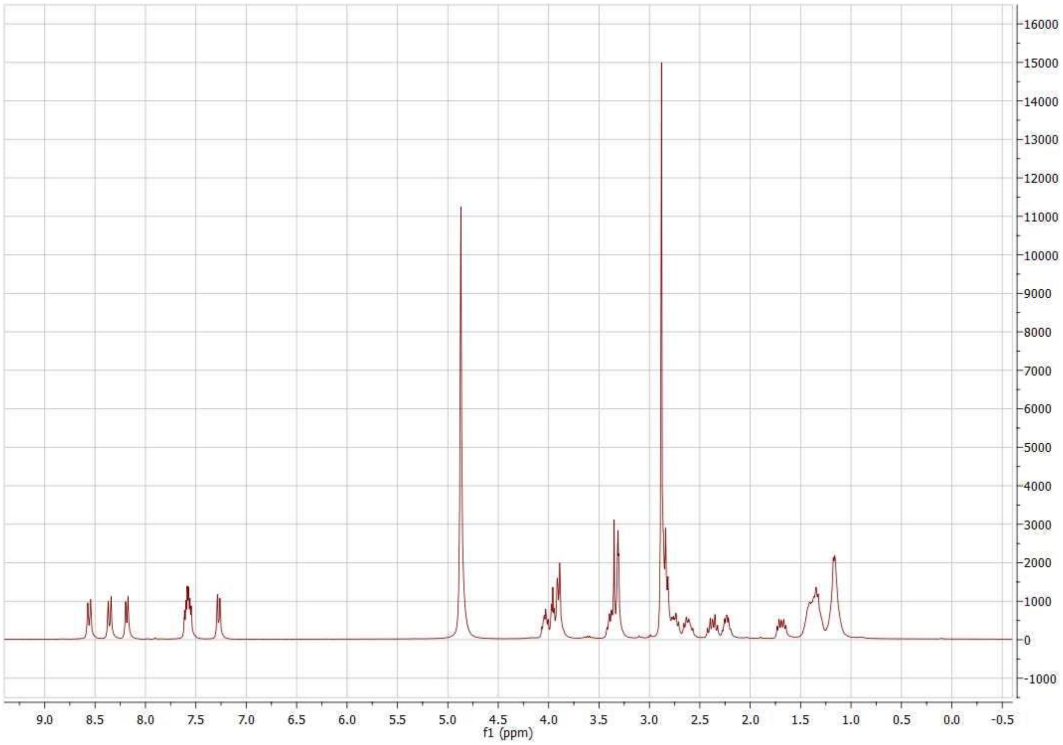

[13]**C NMR** (75.5 MHz, CD_3OD): Compound **31**

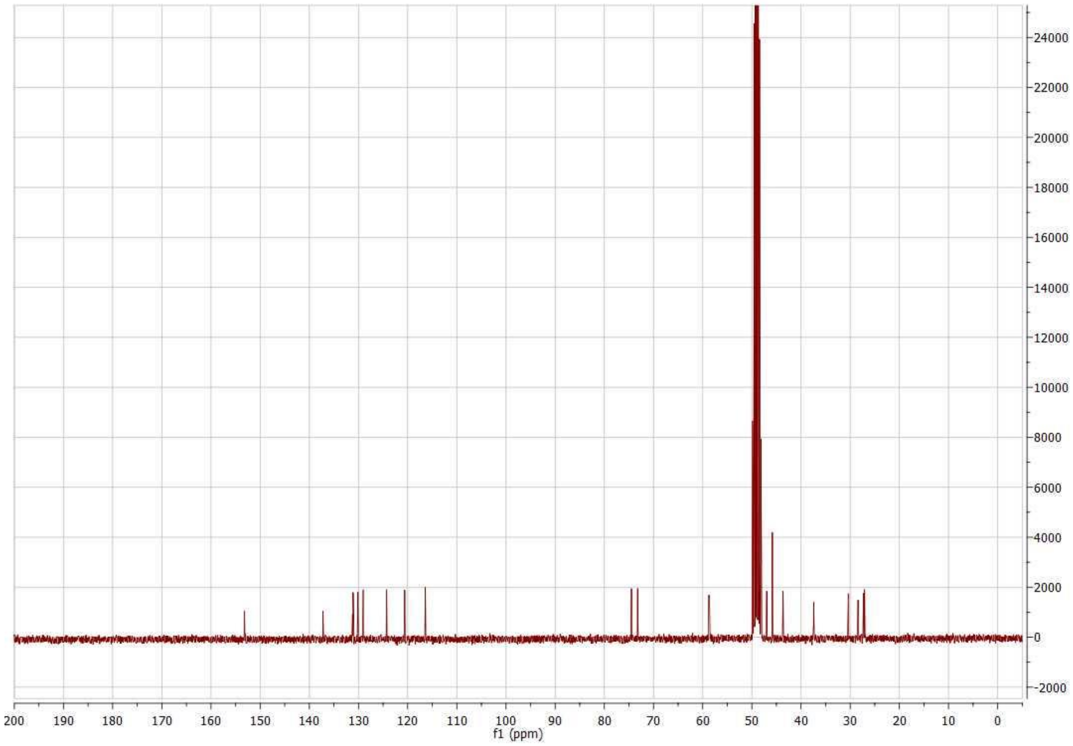

COSY (CD_3OD): Compound **31**

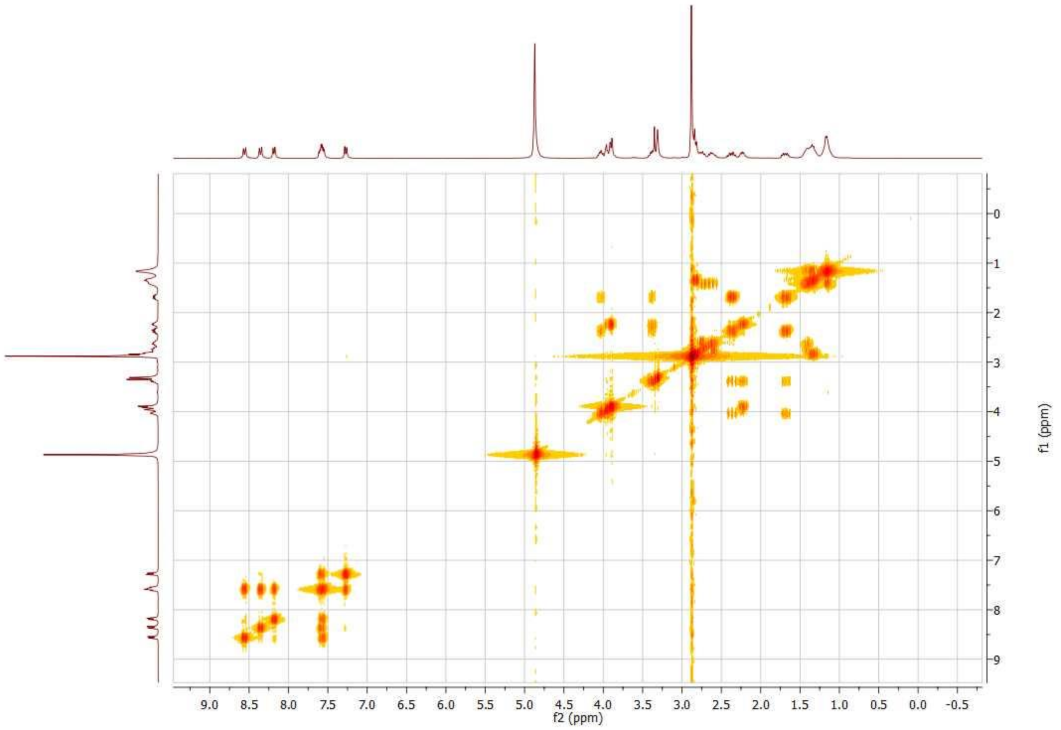

HSQC (CD_3OD): Compound **31**

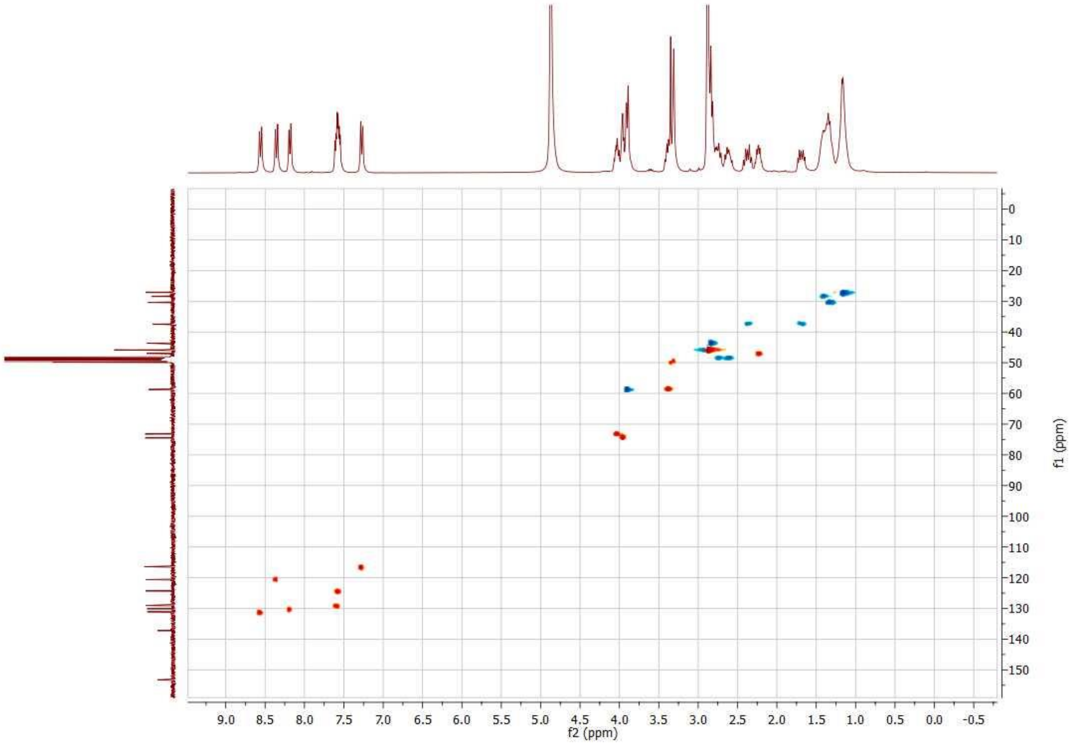

(3a*R*,3b*S*,6a*S*,7*S*,7a*R*)-1-Benzyl-7-fluoro-5,5-dimethylhexahydro-1H-[*1,3*]dioxolo[*4′,5′:3,4*]cyclopenta[*1,2-c*]isoxazole (32)

^{1}**H NMR** (500 MHz, $CDCl_3$): Compound **32**

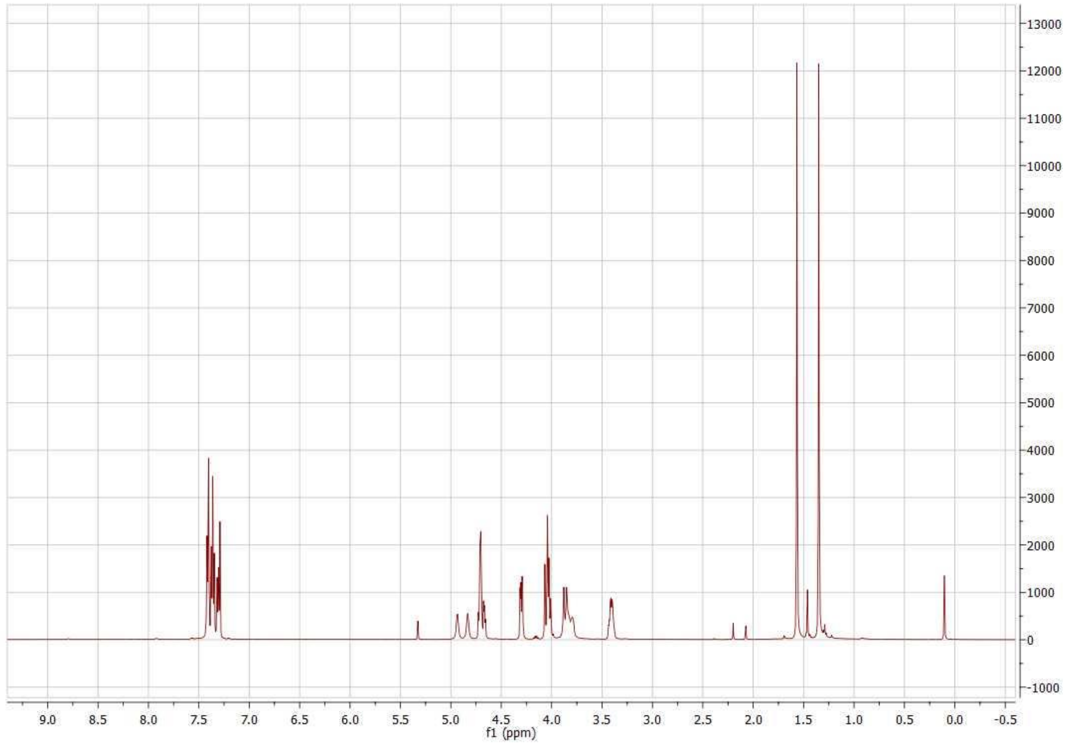

Sample contains traces of CH_2Cl_2, acetone, EtOAc and cyclohexane!

^{13}**C NMR** (75.5 MHz, $CDCl_3$): Compound **32**

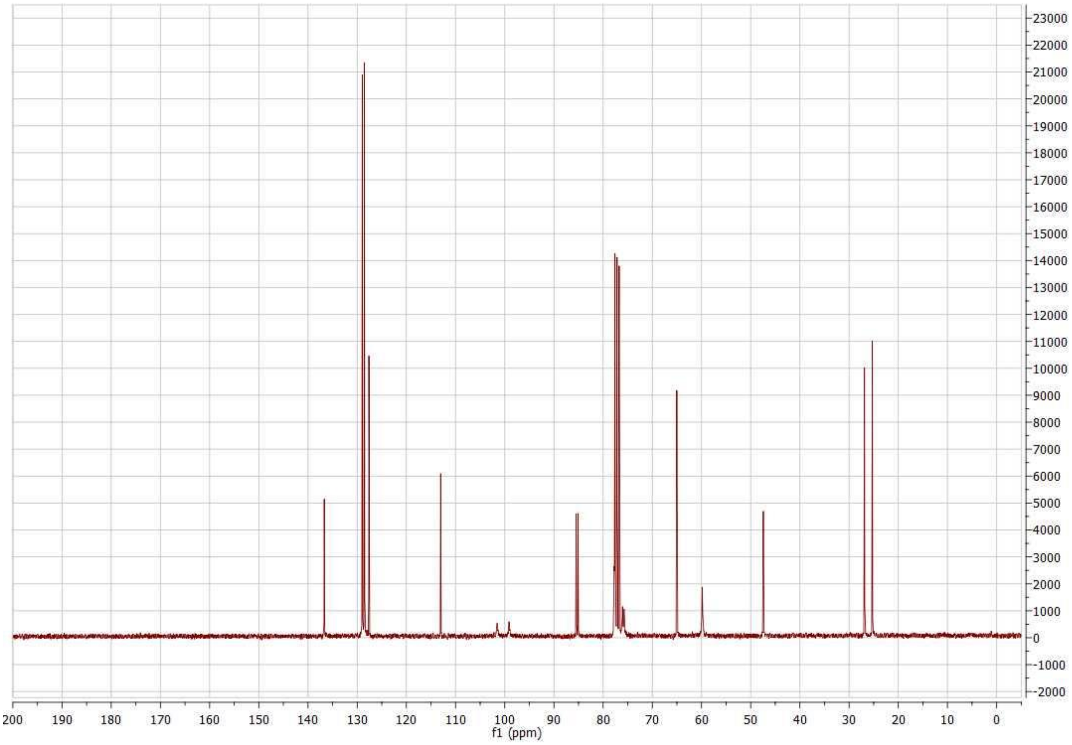

[19]**F NMR** (470.3 MHz, $CDCl_3$): Compound **32**

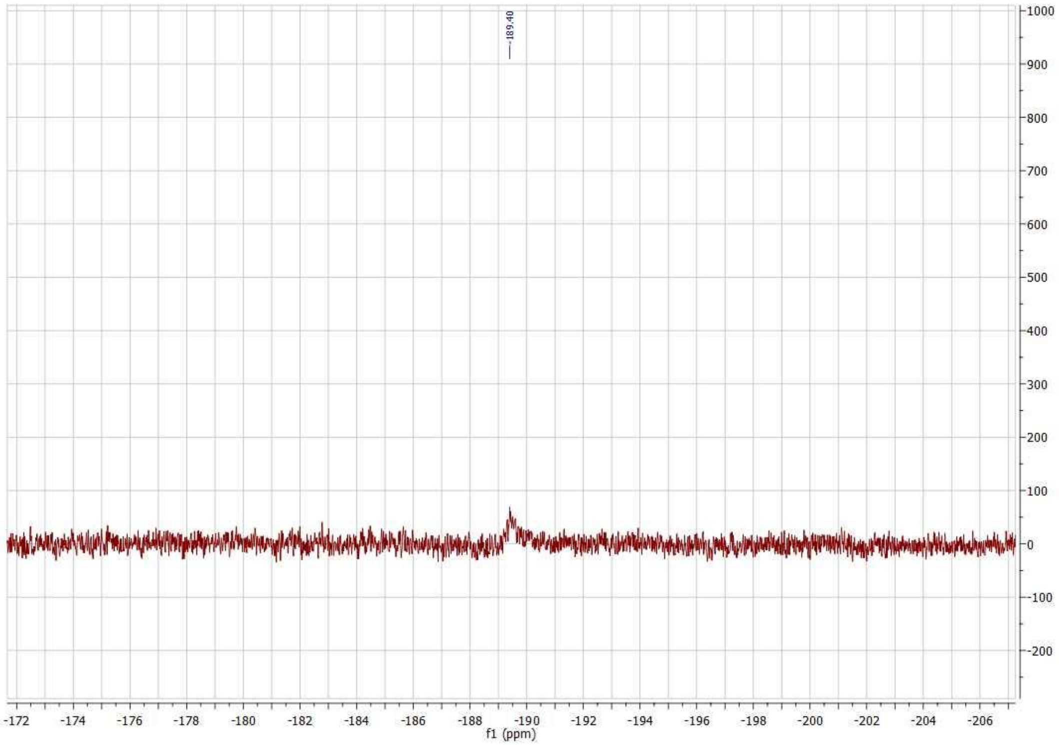

Only a small signal after 72 hours recording time!

COSY ($CDCl_3$): Compound **32**

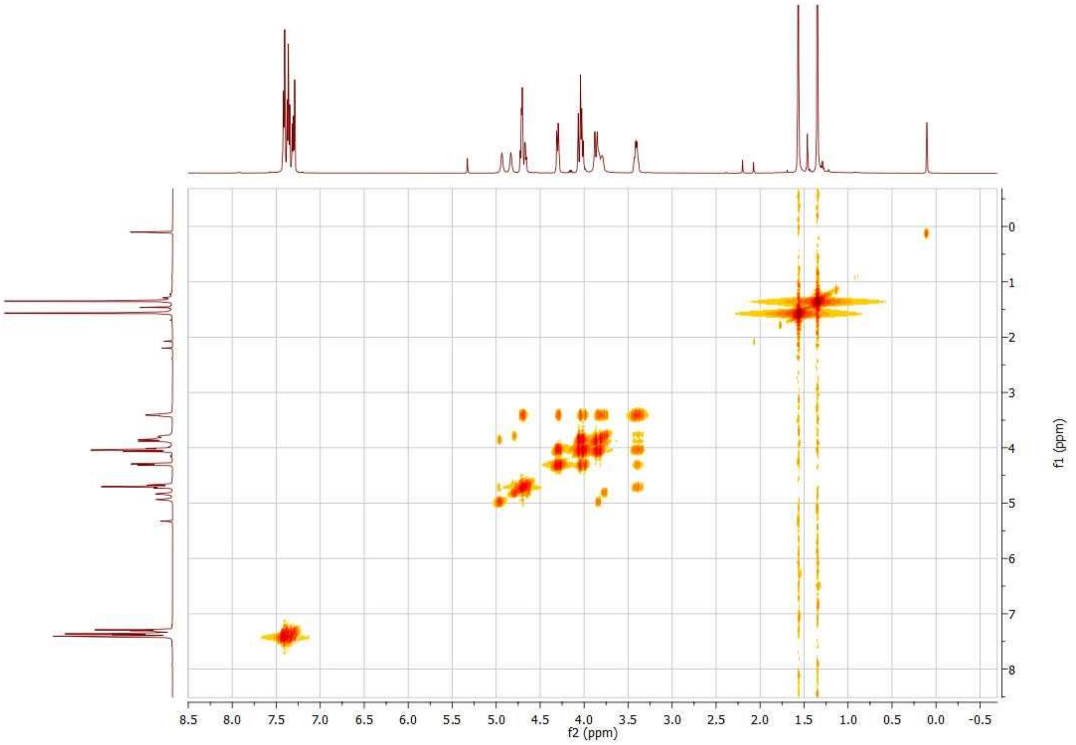

HSQC ($CDCl_3$): Compound **32**

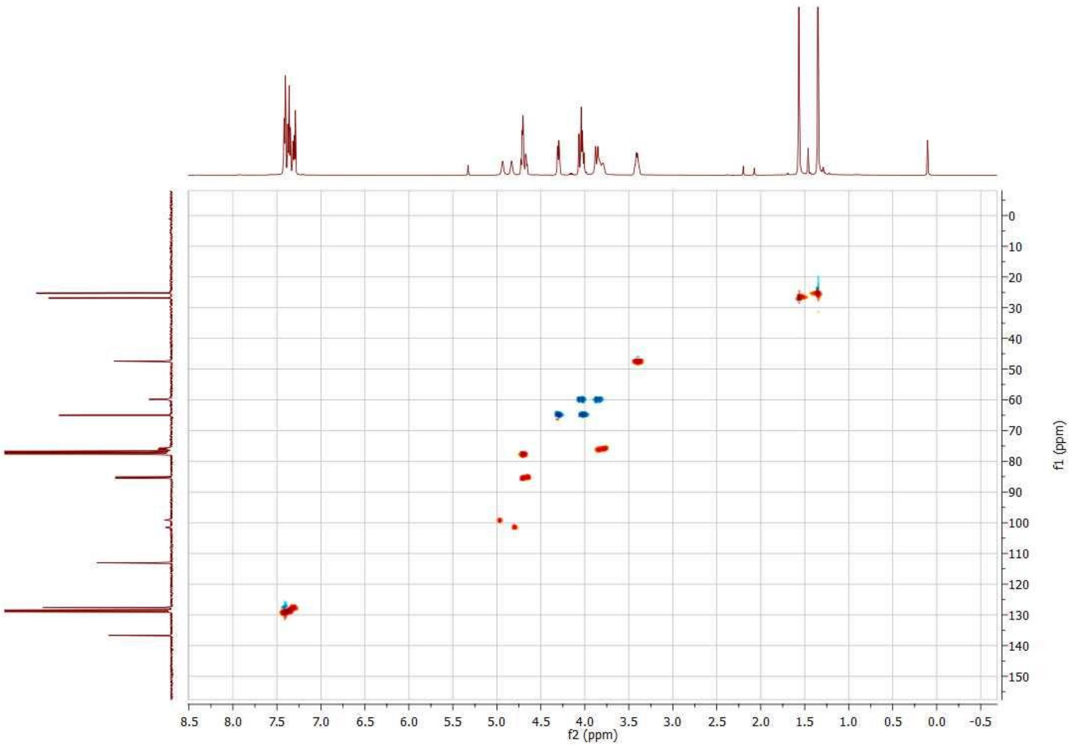

[(3a*S*,4*R*,5*R*,6*S*,6a*S*)-5-Amino-6-fluoro-2,2-dimethyltetrahydro-4H-cyclopenta[*d*][*1,3*]dioxol-4-yl]methanol (33)

[1]**H NMR** (500 MHz, $CDCl_3$): Compound **33**

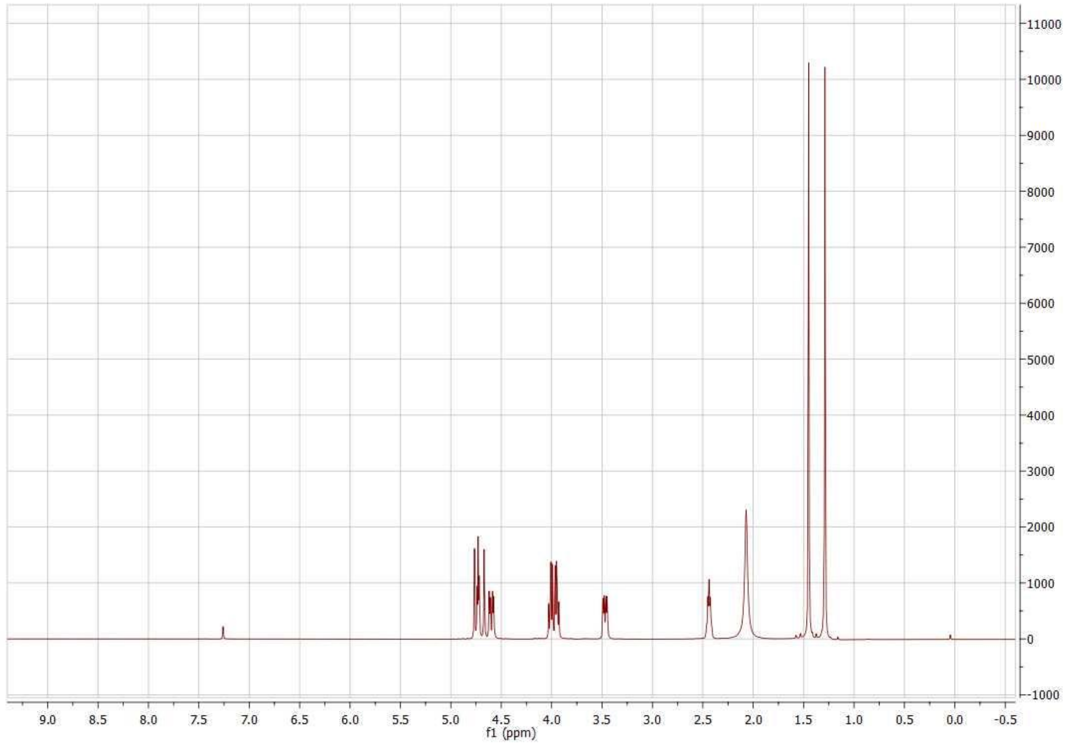

[13]**C NMR** (75.5 MHz, $CDCl_3$): Compound **33**

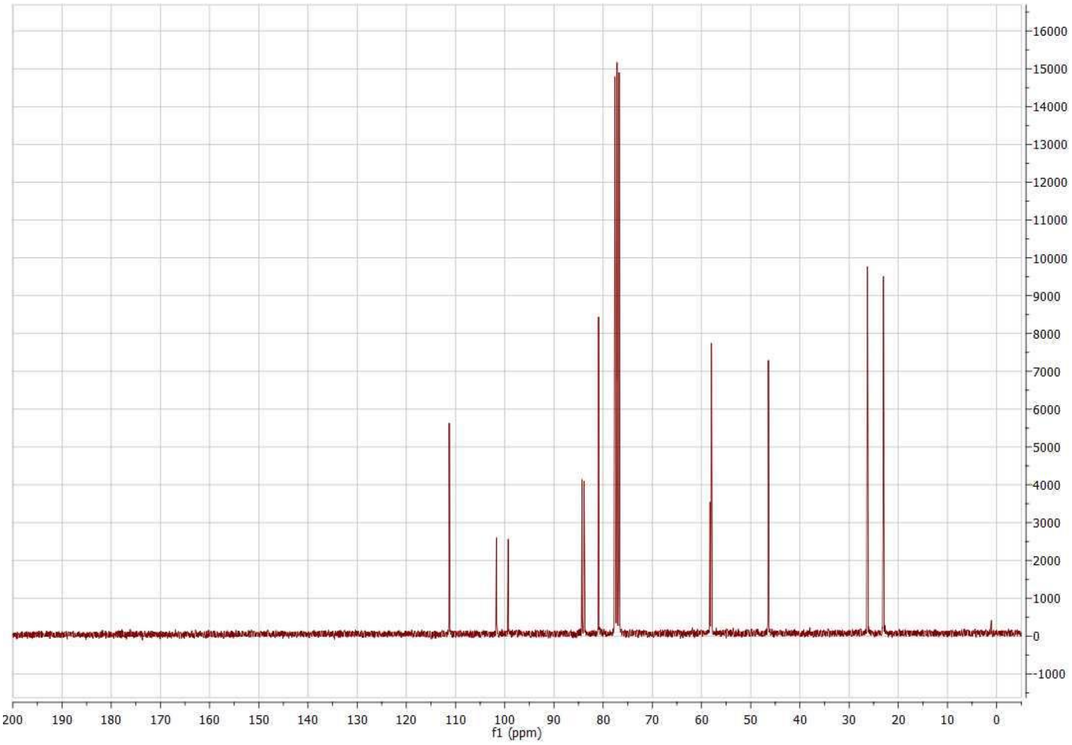

^{19}F NMR (470.3 MHz, $CDCl_3$): Compound **33**

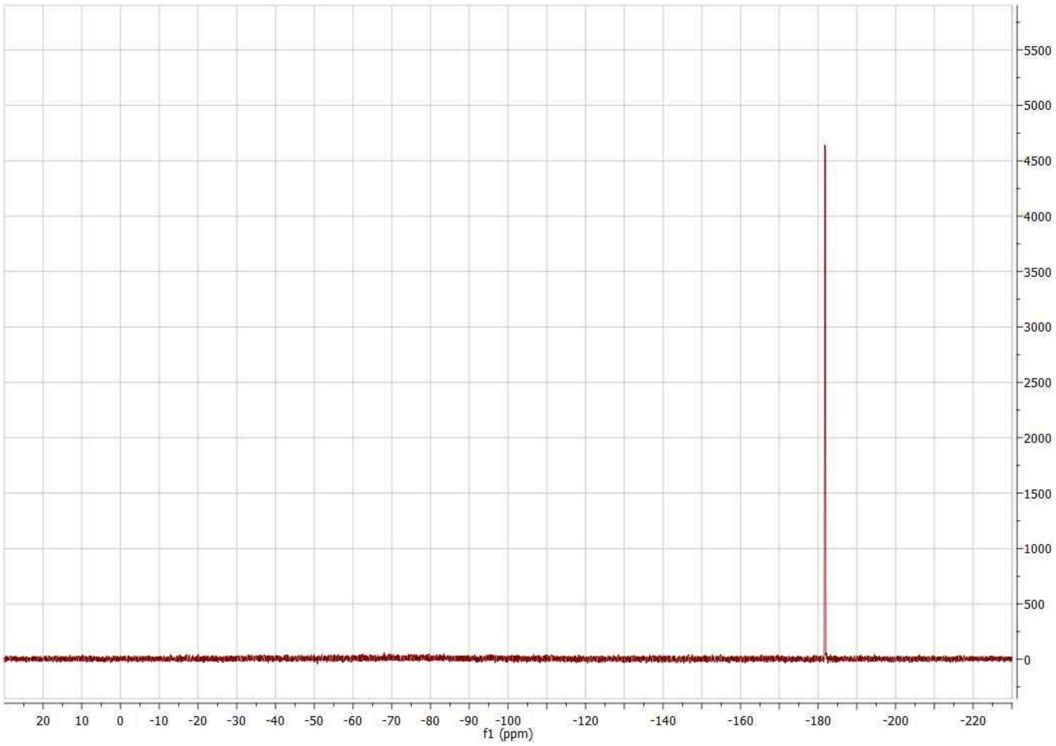

^{19}F NMR (470.3 MHz, $CDCl_3$): Compound **33**

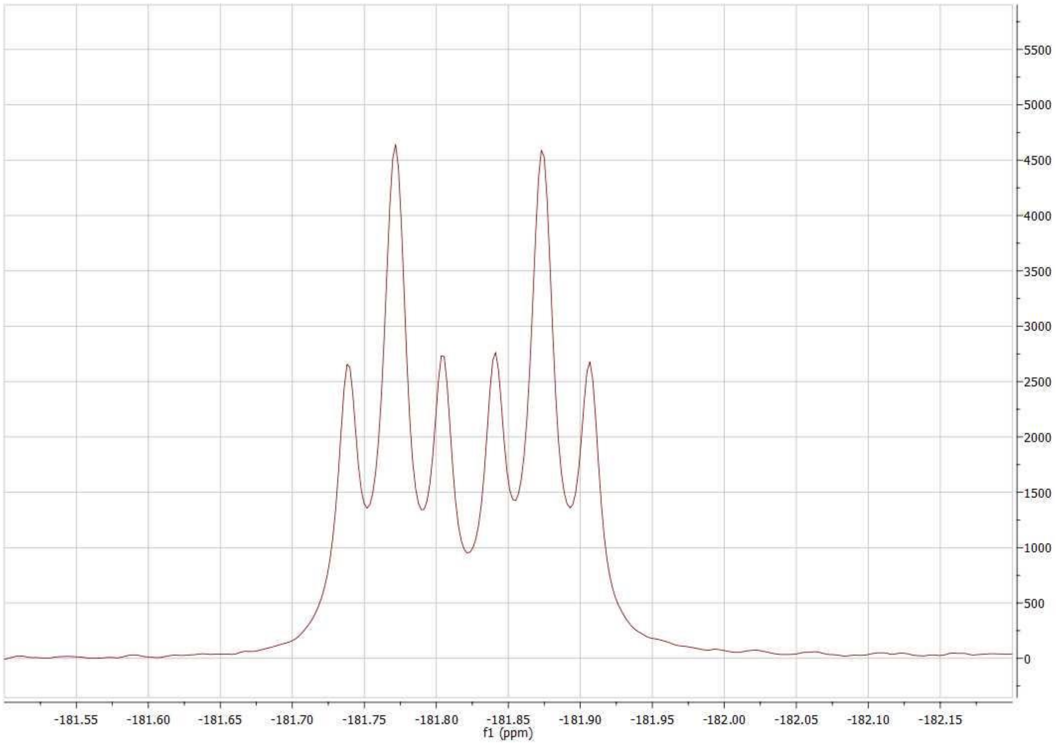

COSY ($CDCl_3$): Compound **33**

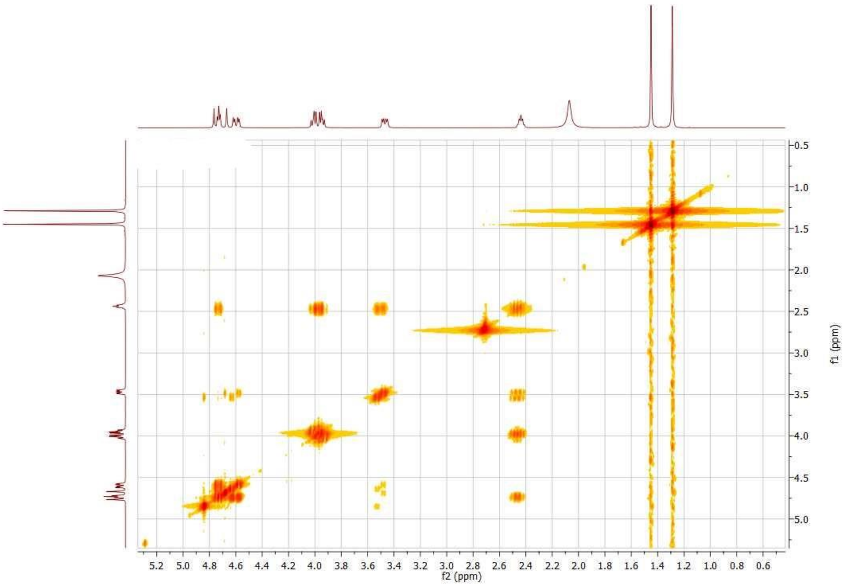

HSQC ($CDCl_3$): Compound **33**

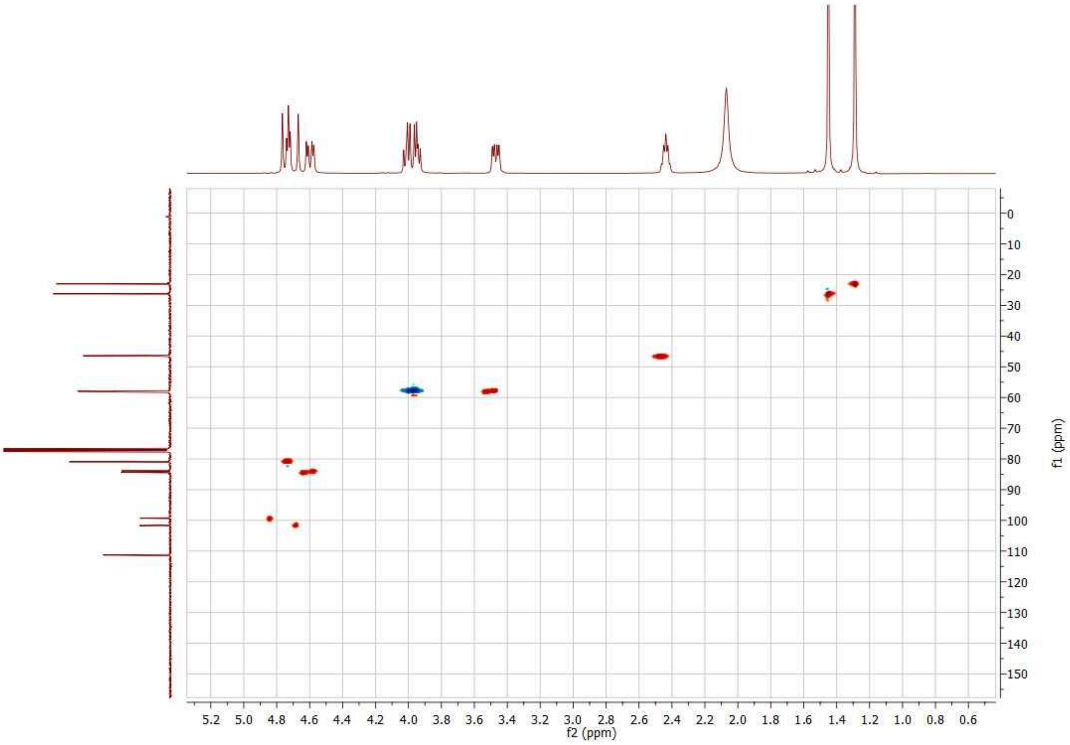

(1*S*,2*S*,3*S*,4*R*,5*R*)-4-Amino-3-fluoro-5-hydroxymethylcyclopentane-1,2-diol (34)

^{1}**H NMR** (500 MHz, D_2O): Compound **34**, free base.

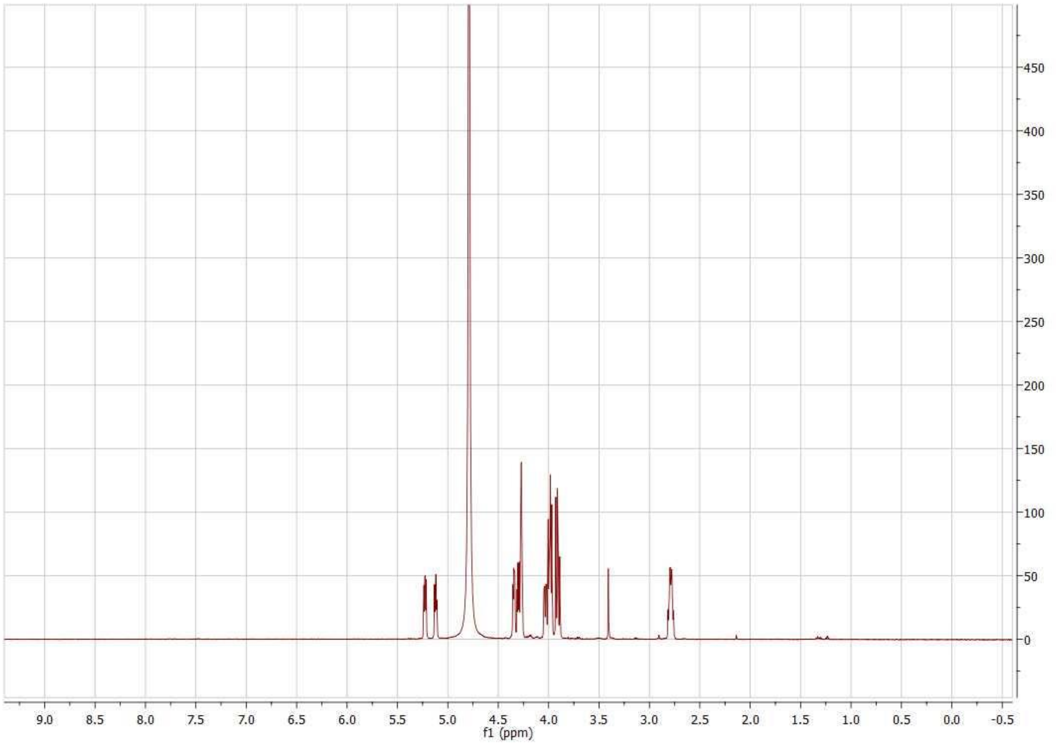

.

^{13}**C NMR** (75.5 MHz, D_2O): Compound **34**, free base.

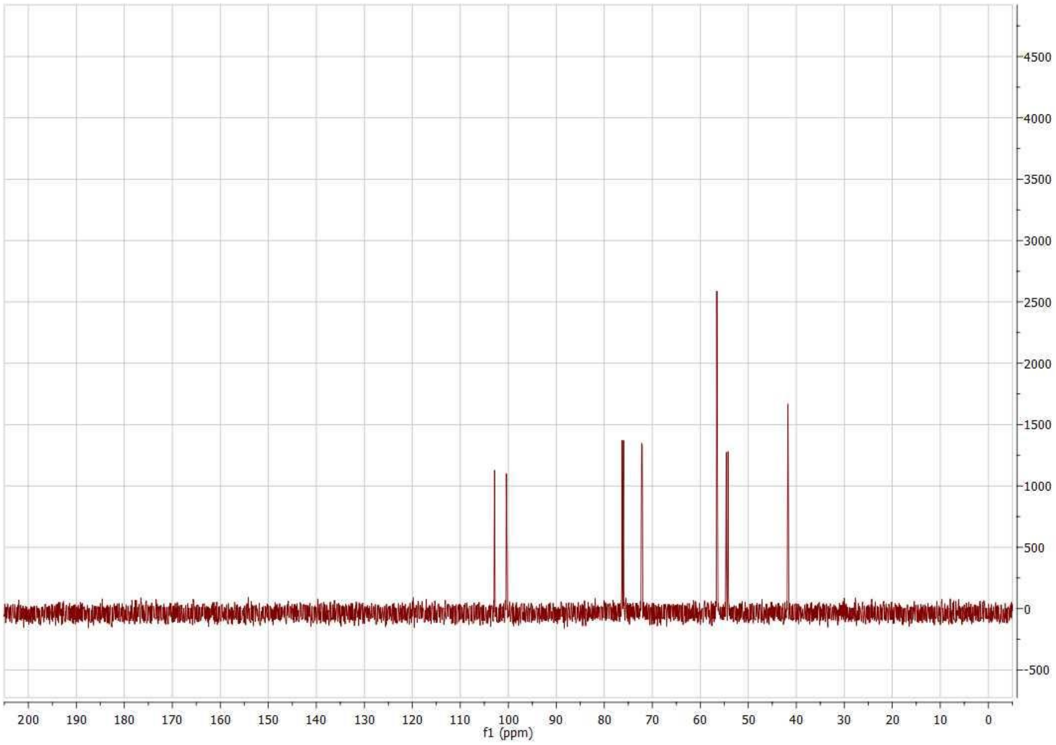

^{19}F NMR (470.3 MHz, D_2O): Compound **34**, free base.

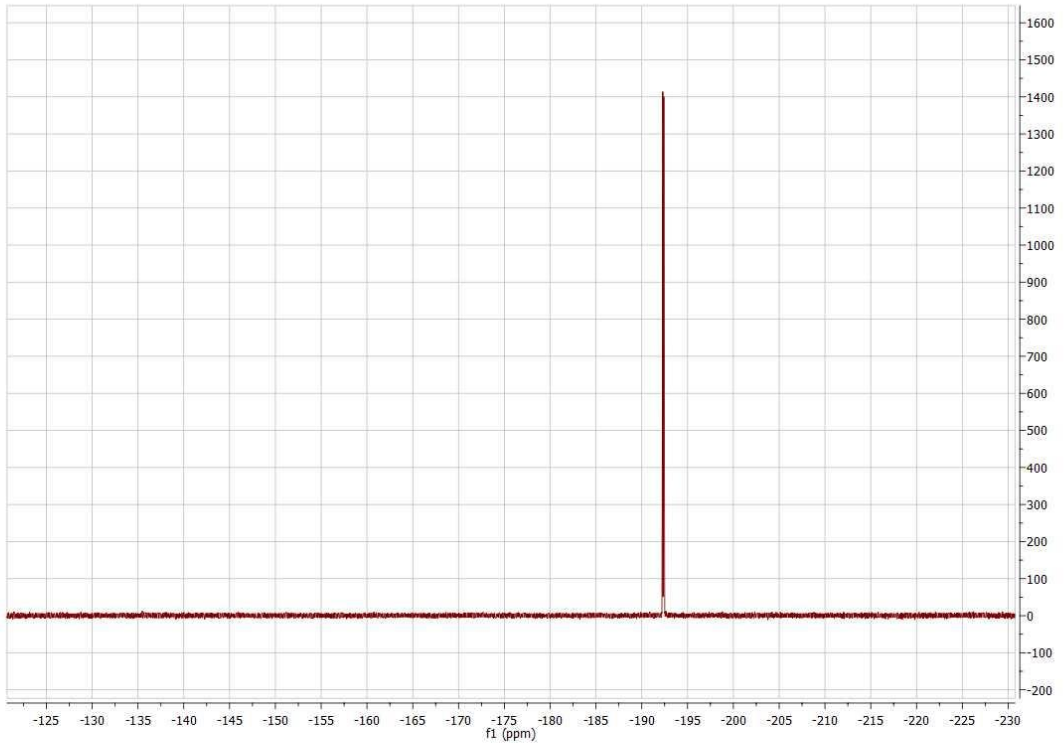

^{19}F NMR (470.3 MHz, D_2O): Compound **34**, free base.

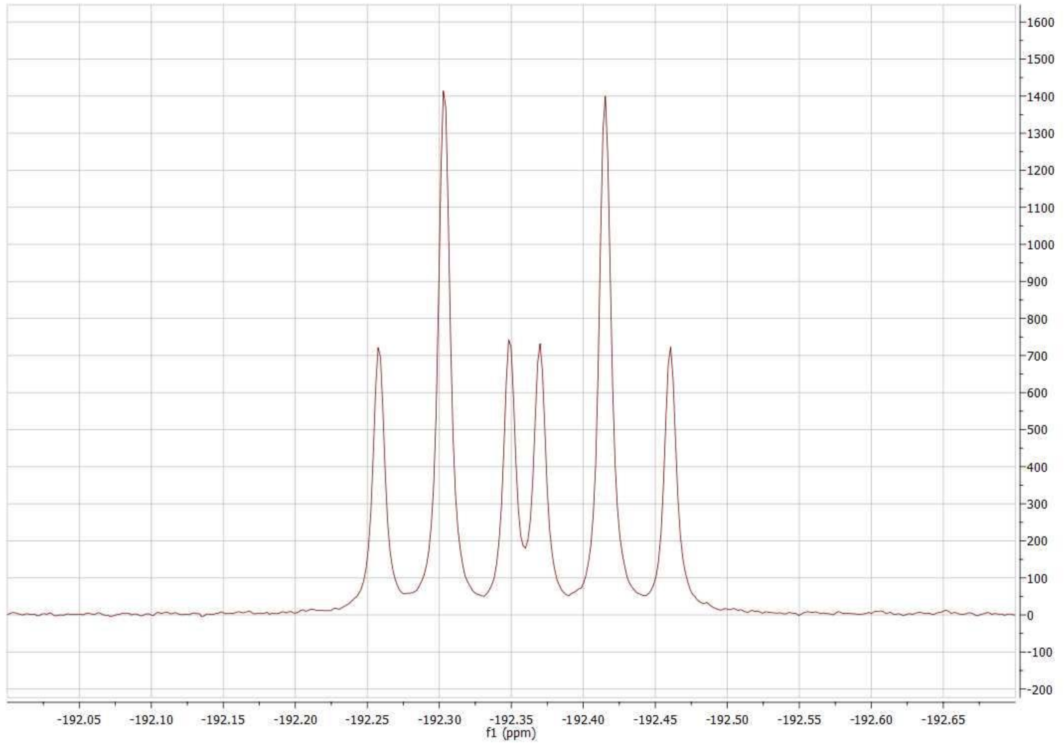

COSY (D_2O): Compound **34**, free base.

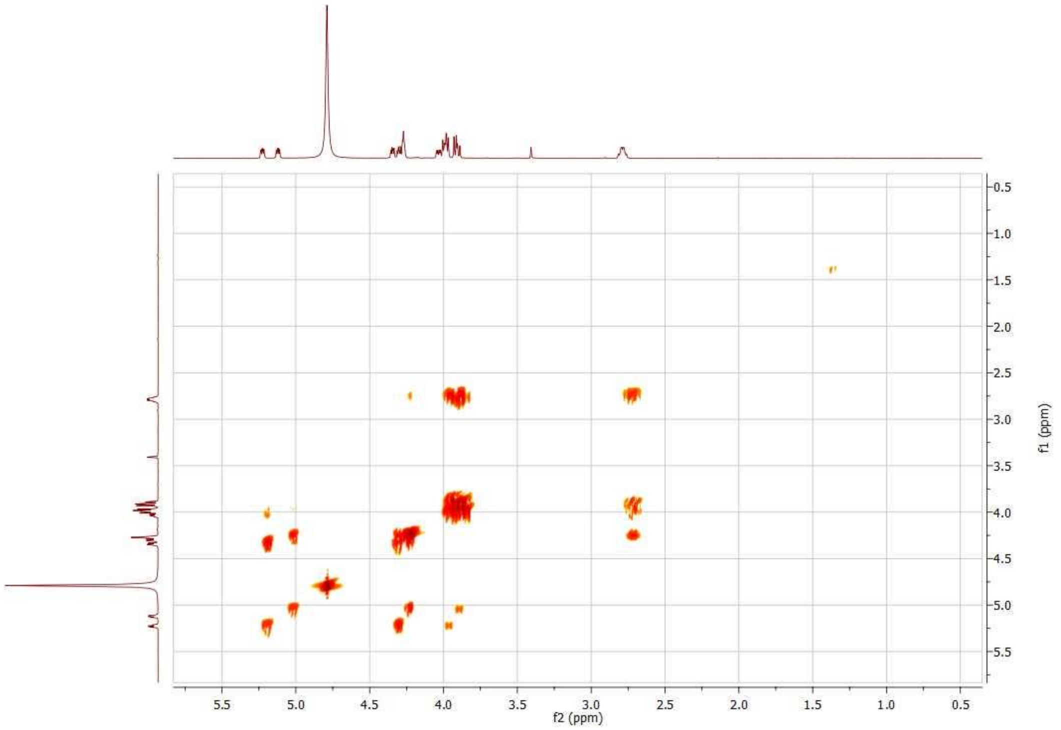

HSQC (D_2O): Compound **34**, free base.

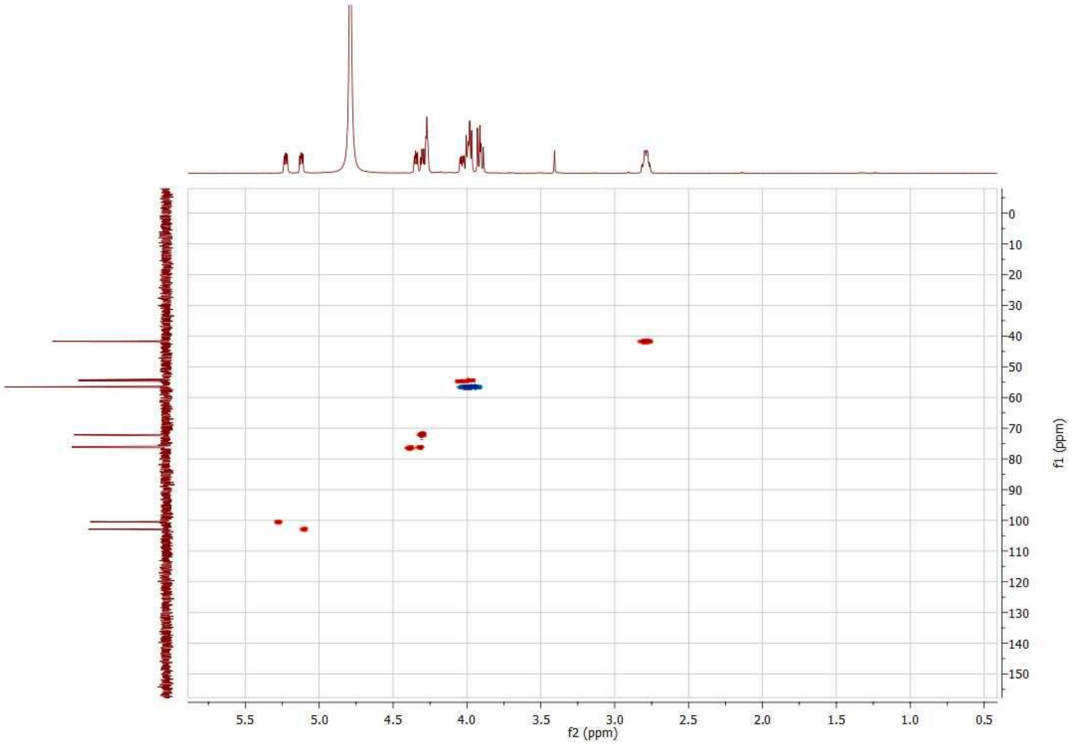

^{1}**H NMR** (500 MHz, D_2O): Compound **34**, hydrochloride.

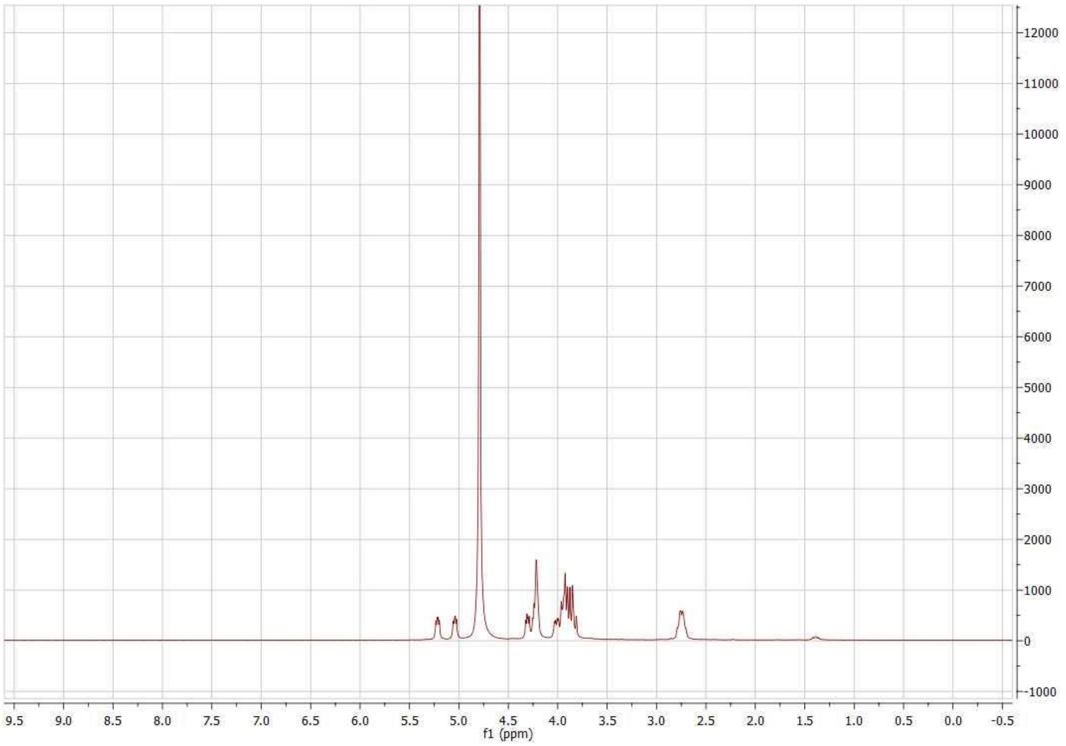

^{13}**C NMR** (75.5 MHz, D_2O): Compound **34**, hydrochloride.

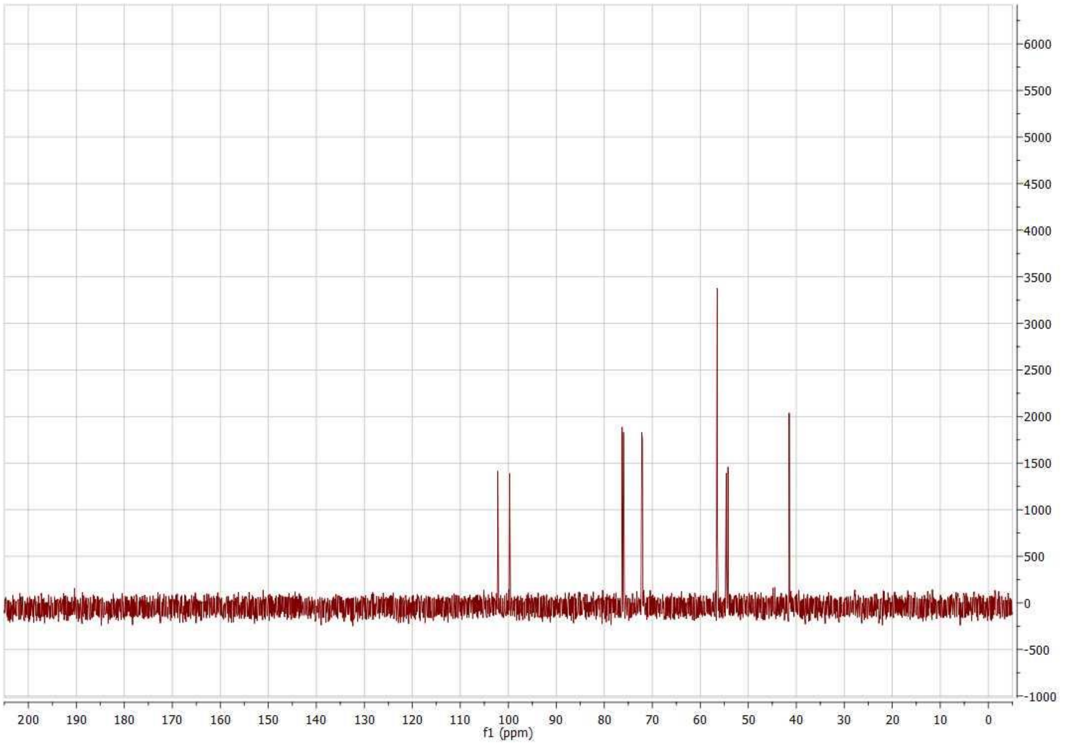

COSY (D_2O): Compound **34**, hydrochloride.

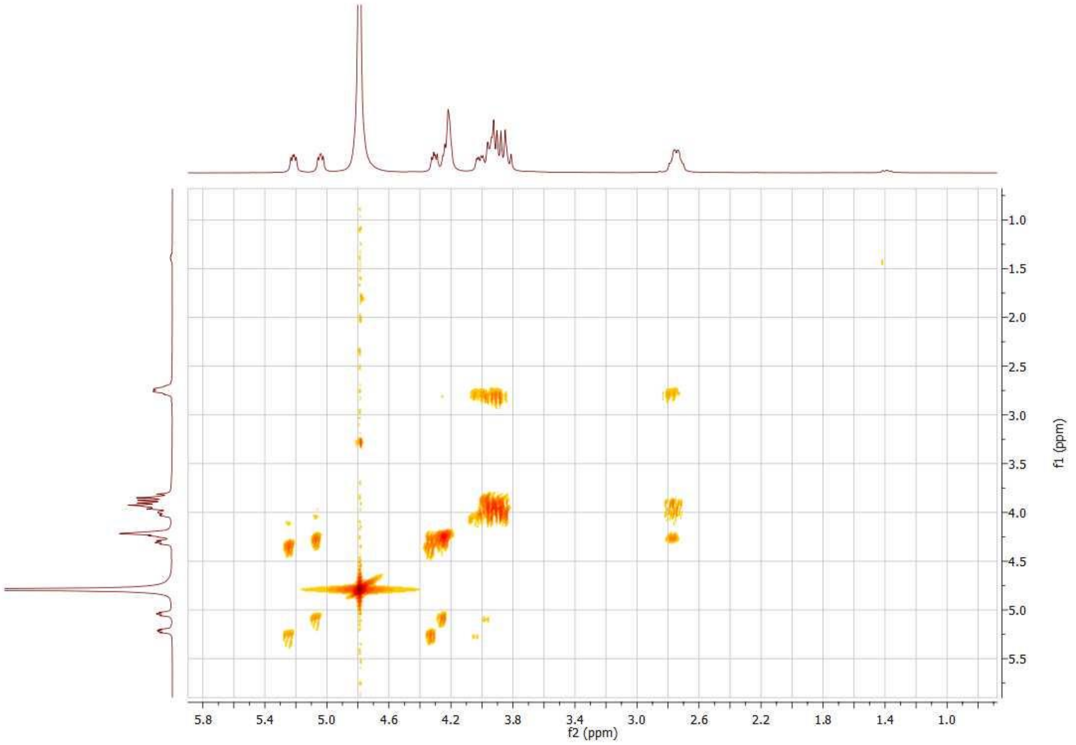

HSQC (D_2O): Compound **34**, hydrochloride.

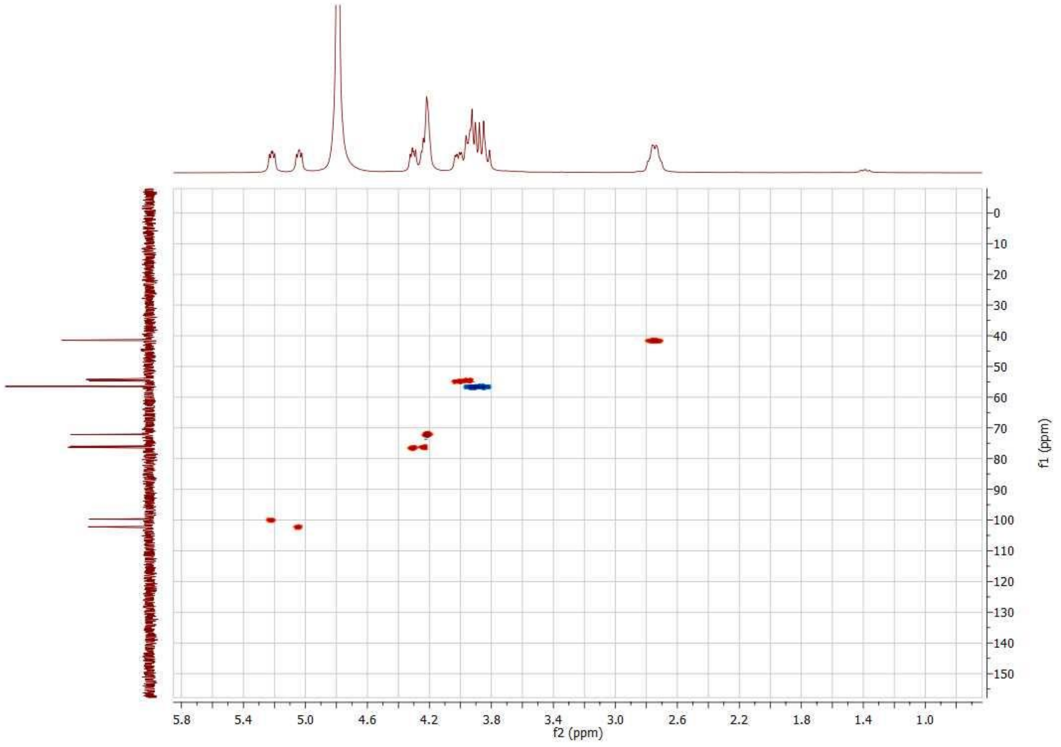

Benzyl 6-(1*R*,2*S*,3*S*,4*S*,5*R*)-2-fluoro-3,4-dihydroxy-5-(hydroxymethyl) cyclopentylamino hexylcarbamate (35)

[1]**H NMR** (500 MHz, CD_3OD): Compound **35**.

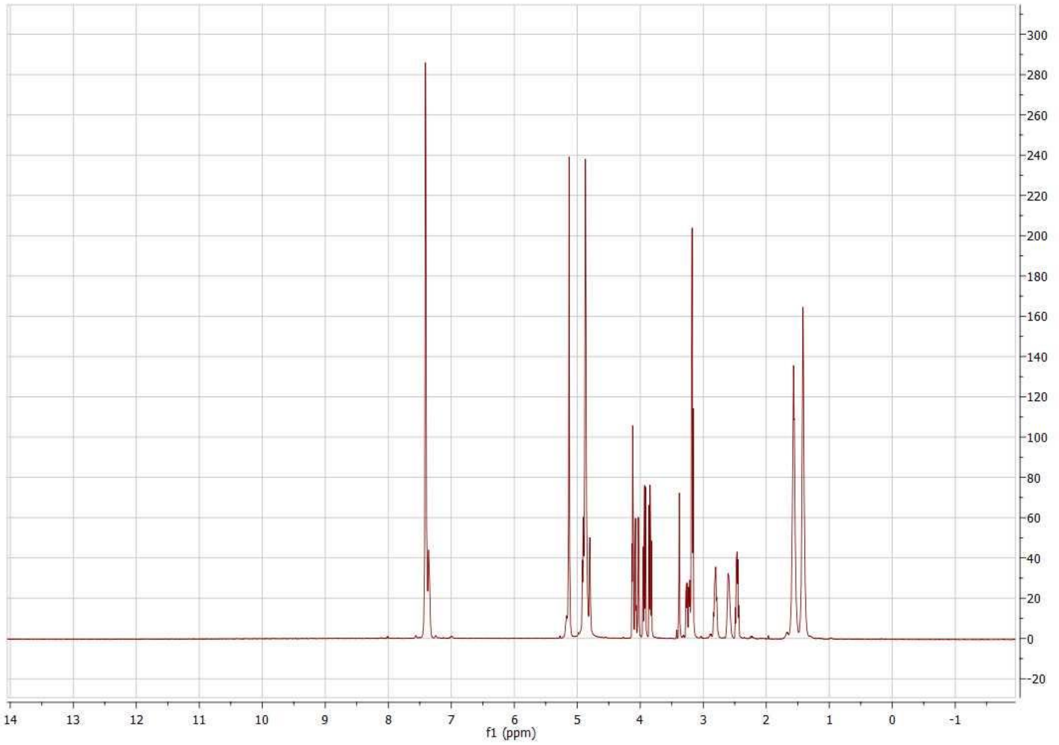

[13]**C NMR** (75.5 MHz, CD_3OD): Compound **35**.

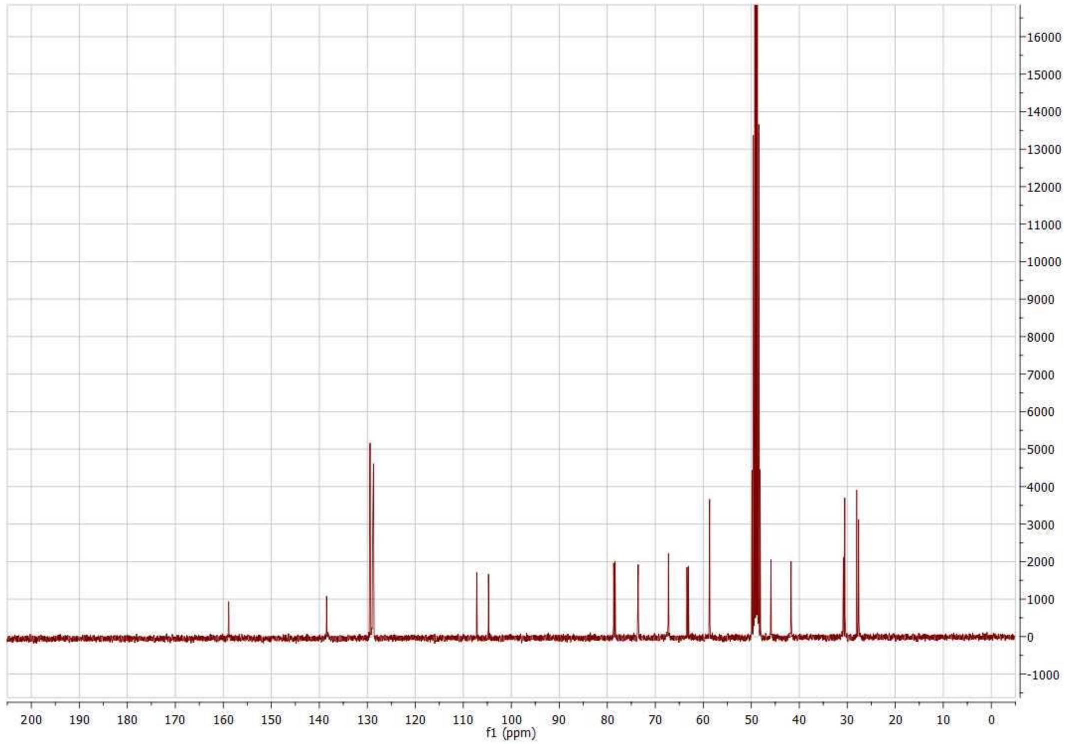

^{19}F NMR (470.3 MHz, CD_3OD): Compound **35**.

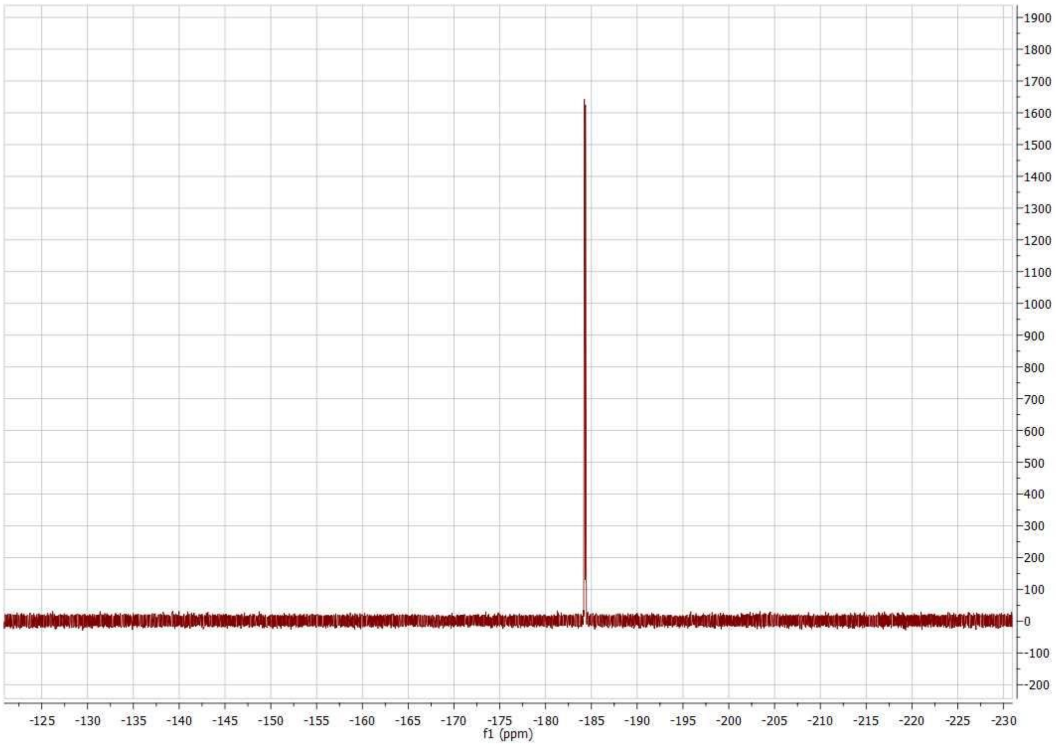

^{19}F NMR (470.3 MHz, CD_3OD): Compound **35**.

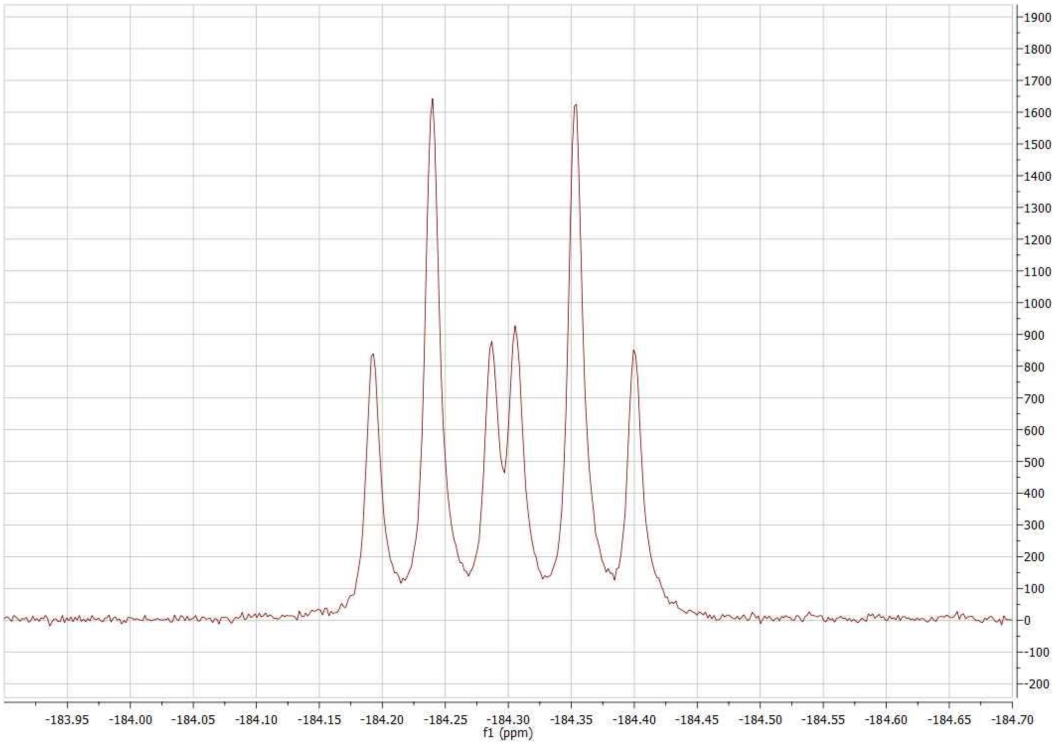

COSY (CD_3OD): Compound **35**.

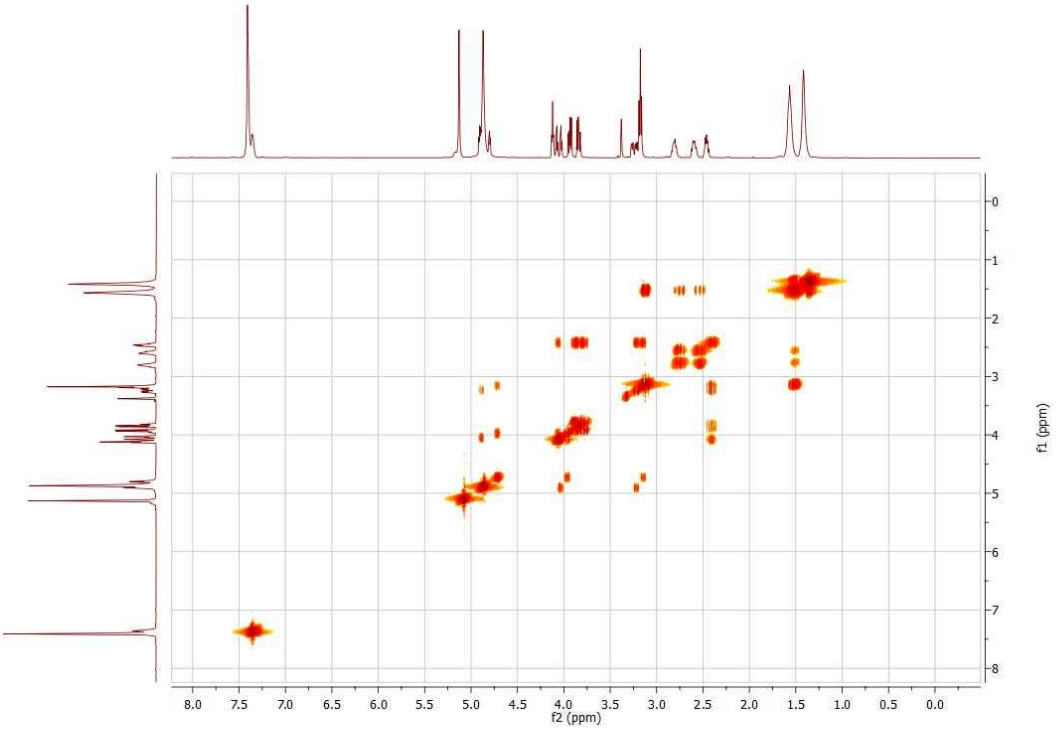

HSQC (CD_3OD): Compound **35**.

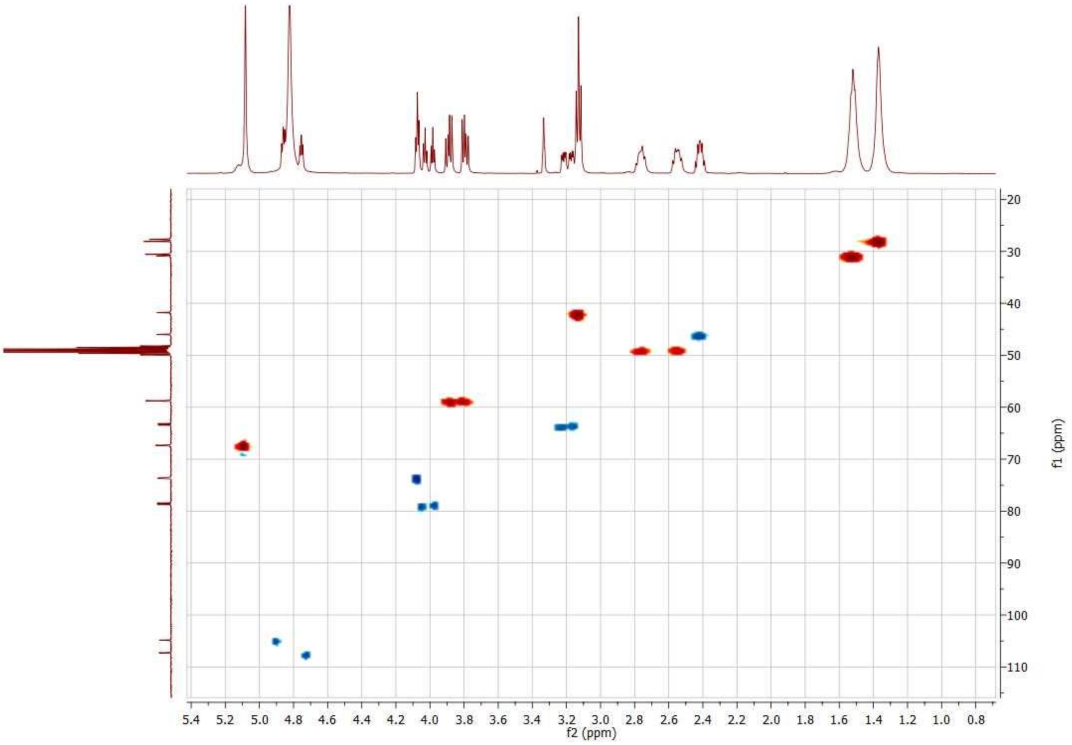

(1*S*,2*S*,3*S*,4*R*,5*R*)-4-(6′-Dansylaminohexyl)amino-3-fluoro-5-hydroxymethylcyclopentane-1,2-diol (37)

^{1}H NMR (500 MHz, CD_3OD): Compound **37**.

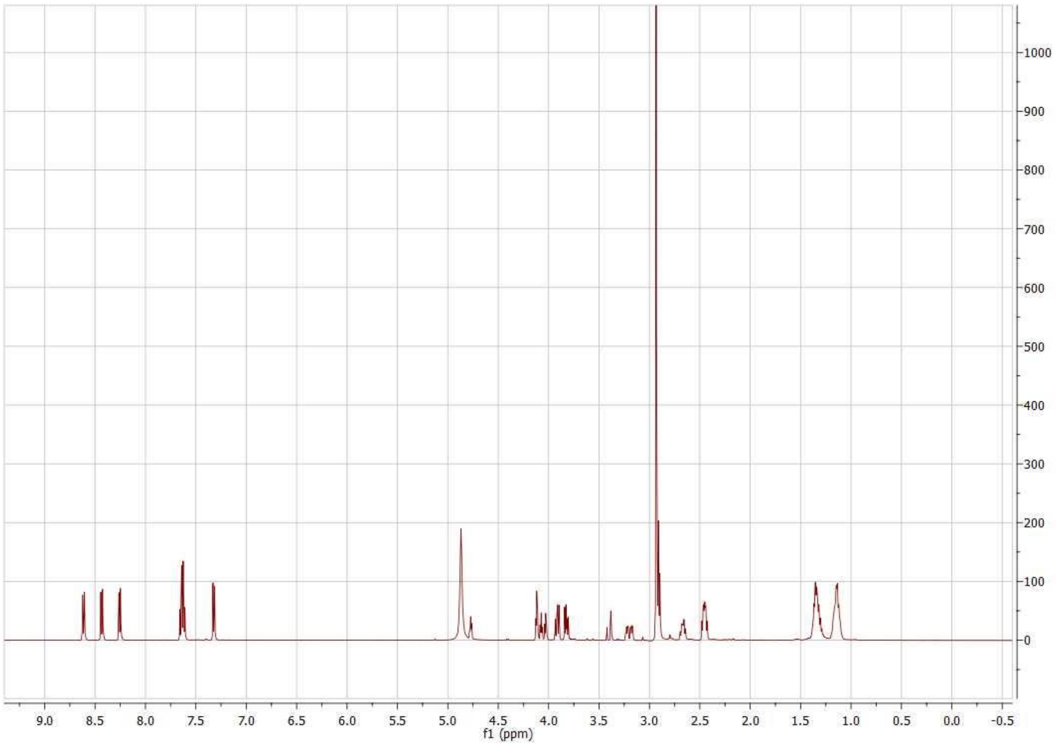

^{13}C NMR (75.5 MHz, CD_3OD): Compound **37**.

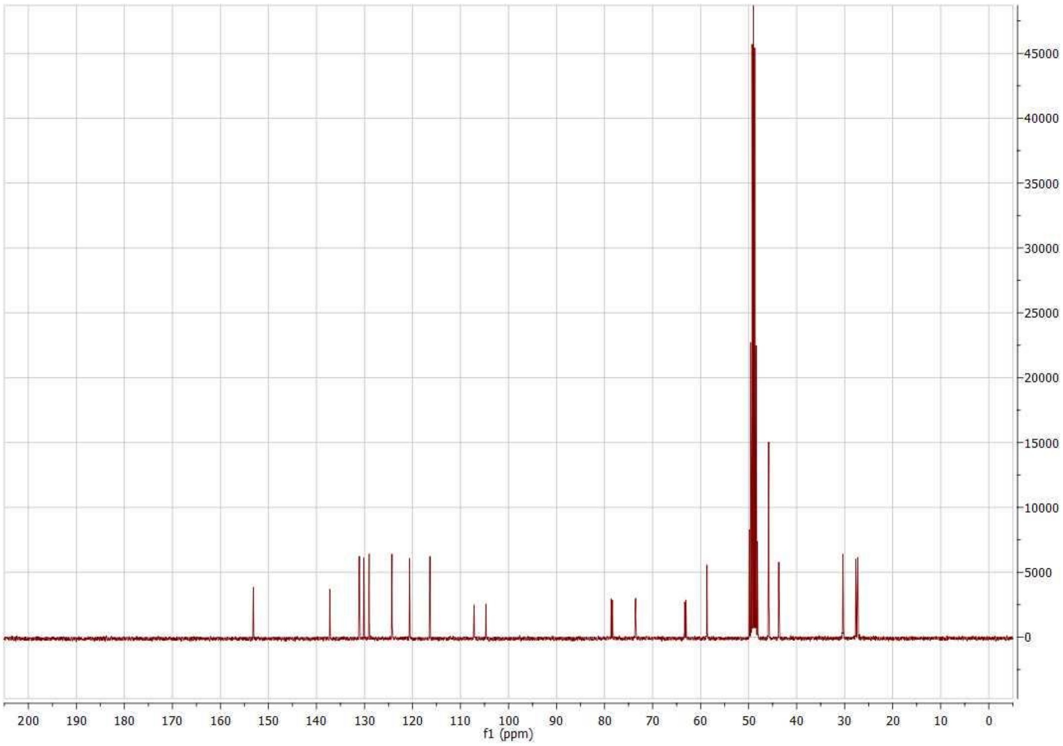

^{19}F NMR (470.3 MHz, CD_3OD): Compound **37**.

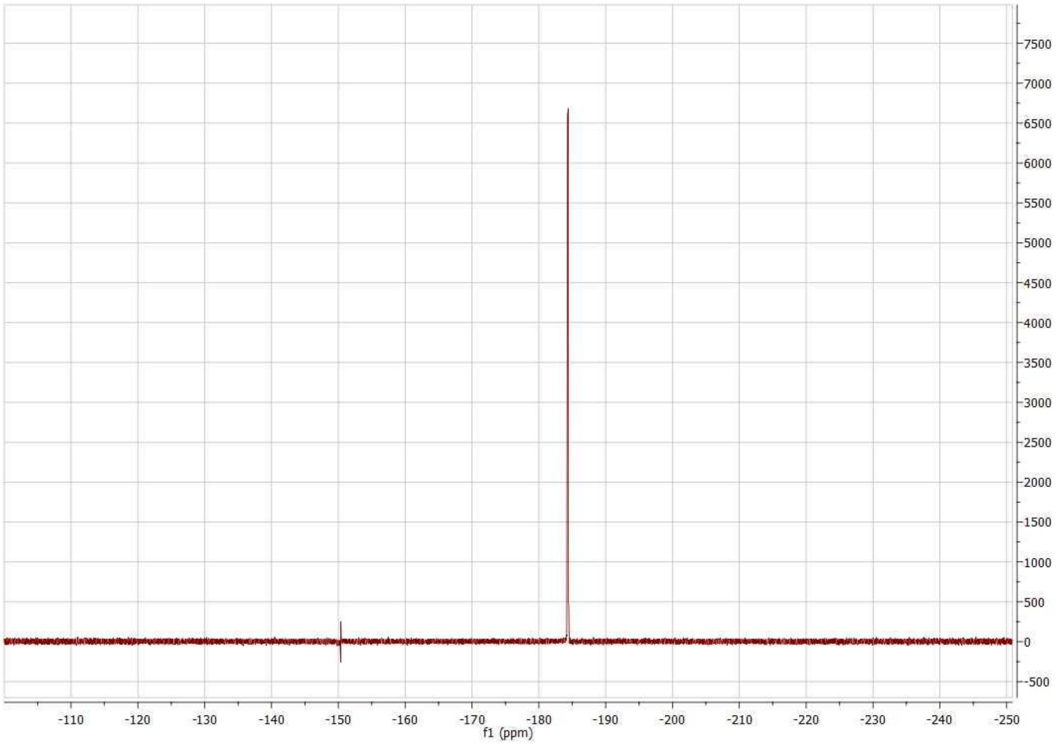

^{19}F NMR (470.3 MHz, CD_3OD): Compound **37**.

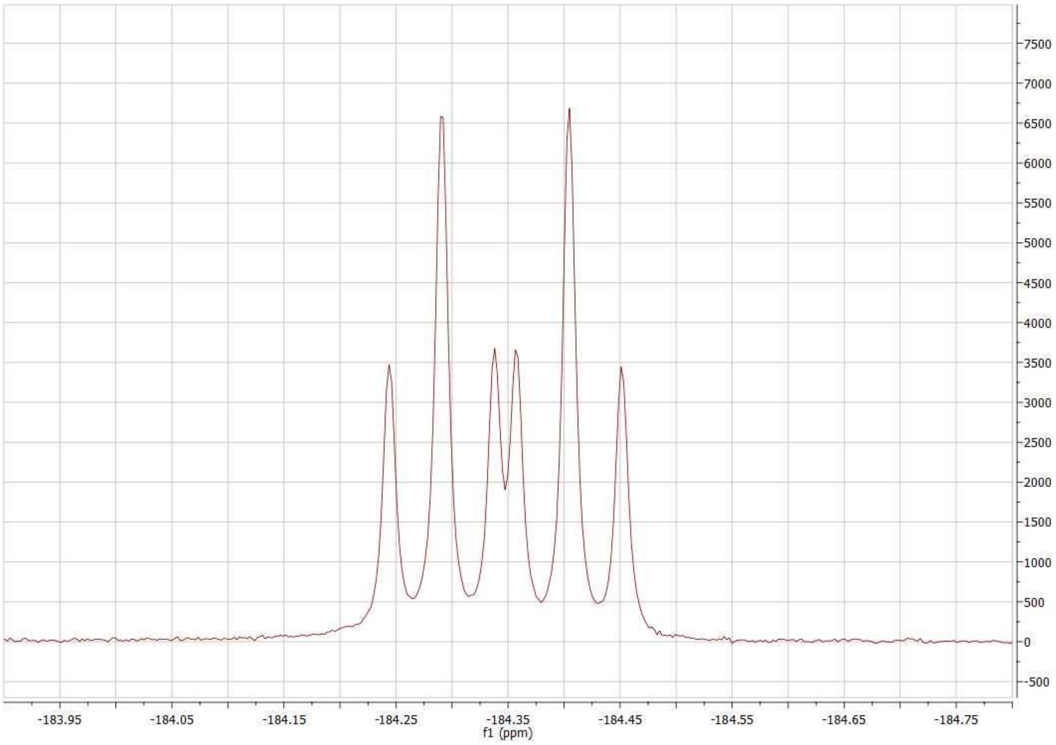

COSY (CD_3OD): Compound **37**.

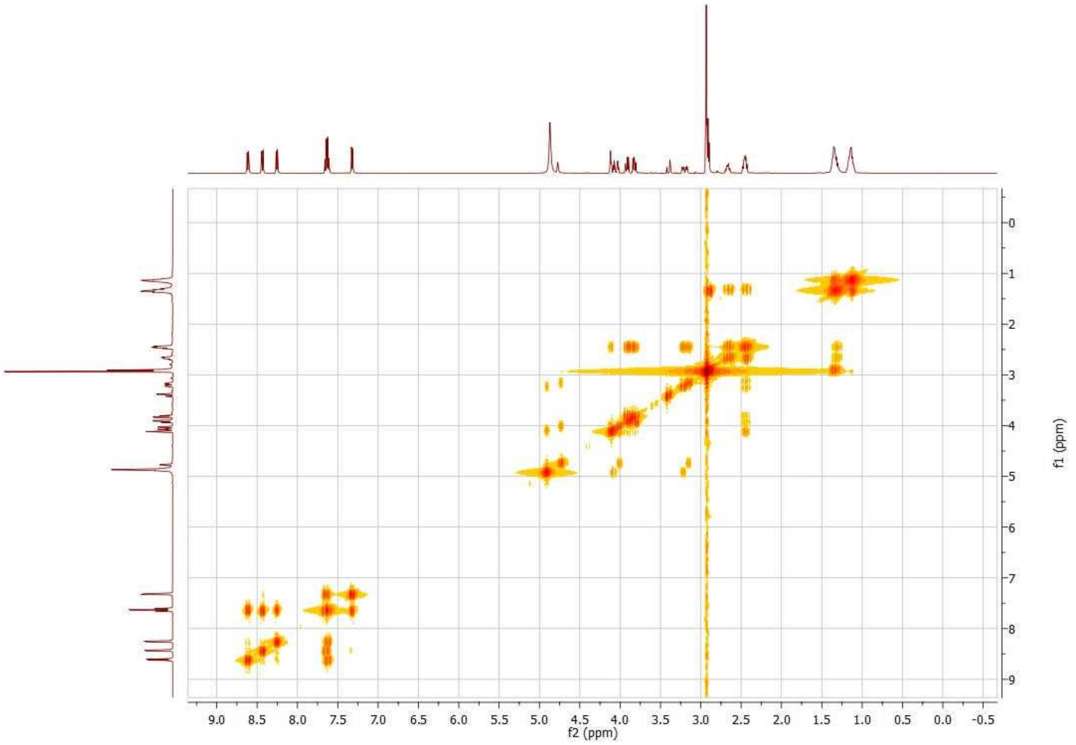

HSQC (CD_3OD): Compound **37**.

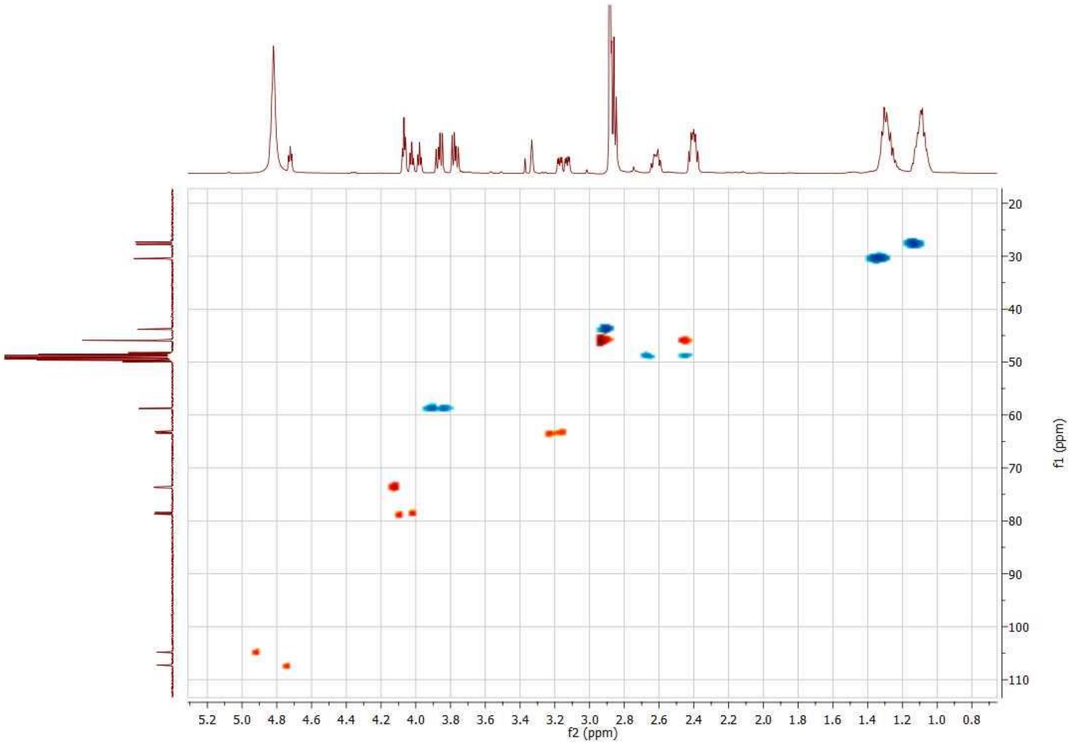

1. Bruker *APEX2 and SAINT*, Bruker AXS Inc.: Madison, Wisconsin, USA, 2012.
2. Blessing, R. H. An empirical correction for absorption anisotropy. *Acta Crystallogr., Sect. A: Found. Adv.* **1995,** *51* (1), 33-38. doi: 10.1107/S0108767394005726.
3. Sheldrick, G. M. *SADABS, Version 2.10, Siemens Area Detector Correction*, Universität Göttingen, Germany, **2003**
4. Sheldrick, G. M. SHELXT - Integrated space-group and crystal-structure determination. *Acta Crystallogr., Sect. A: Found. Adv.* **2015,** *71* (1), 3-8. doi: 10.1107/S2053273314026370.
5. Sheldrick, G. M. Phase annealing in SHELX-90: direct methods for larger structures. *Acta Crystallogr., Sect. A: Found. Adv.* **1990,** *46* (6), 467-473. doi: 10.1107/S0108767390000277.
6. Sheldrick, G. M. A short history of SHELX. *Acta Crystallogr., Sect. A: Found. Adv.* **2008,** *64* (1), 112-122. doi: 10.1107/S0108767307043930.
7. Sheldrick, G. M. Crystal structure refinement with SHELXL. *Acta Crystallogr., Sect. C: Struct. Chem.* **2015,** *71* (1), 3-8. doi: 10.1107/S2053229614024218.
8. Sheldrick, G. M. *SHELXS97*, Univ. Göttingen, Germany, **1997**.
9. Huebschle, C. B.; Sheldrick, G. M.; Dittrich, B. ShelXle: a Qt graphical user interface for SHELXL. *J. Appl. Crystallogr.* **2011,** *44* (Copyright (C) 2014 American Chemical Society (ACS). All Rights Reserved.), 1281-1284. doi: 10.1107/S0021889811043202.
10. Spek, A. L. Single-crystal structure validation with the program PLATON. *J. Appl. Crystallogr.* **2003,** *36* (1), 7-13. doi: 10.1107/S0021889802022112.
11. Spek, A. L. Structure validation in chemical crystallography. *Acta Crystallogr., Sect. D: Biol. Crystallogr.* **2009,** *65* (2), 148-155. doi: Structure validation in chemical crystallography.
12. Spek, A. L. PLATON SQUEEZE: a tool for the calculation of the disordered solvent contribution to the calculated structure factors. *Acta Crystallogr., Sect. C: Struct. Chem.* **2015,** *71* (1), 9-18. doi: 10.1107/S2053229614024929.
13. Putz, H. Brandenburg, K. *Diamond - Crystal and Molecular Structure Visualization*, 3.2i; Crystal Impact: Bonn, Germany.
14. Allen, F. H.; Johnson, O.; Shields, G. P.; Smith, B. R.; Towler, M. CIF applications. XV. enCIFer: a program for viewing, editing and visualizing CIFs. *J. Appl. Crystallogr.* **2004,** *37* (2), 335-338. doi: 10.1107/S0021889804003528.
15. Westrip, S. publCIF: software for editing, validating and formatting crystallographic information files. *J. Appl. Crystallogr.* **2010,** *43* (4), 920-925. doi: 10.1107/S0021889810022120.
